# Mobile Health Apps on COVID-19 Launched in the Early Days of the Pandemic: Content Analysis and Review

**DOI:** 10.2196/19796

**Published:** 2020-09-16

**Authors:** Long Chiau Ming, Noorazrina Untong, Nur Amalina Aliudin, Norliza Osili, Nurolaini Kifli, Ching Siang Tan, Khang Wen Goh, Pit Wei Ng, Yaser Mohammed Al-Worafi, Kah Seng Lee, Hui Poh Goh

**Affiliations:** 1 Pengiran Anak Puteri Rashidah Sa’adatul Bolkiah Institute of Health Sciences Universiti Brunei Darussalam Gadong Brunei Darussalam; 2 School of Pharmacy KPJ Healthcare University College Nilai Malaysia; 3 Faculty of Science and Technology Quest International University Perak Ipoh Malaysia; 4 Department of Pharmacy National University Health System Singapore Singapore; 5 College of Pharmacy University of Science and Technology of Fujairah Fujairah United Arab Emirates; 6 College of Pharmacy University of Science and Technology Sana’a Yemen; 7 Faculty of Pharmacy University of Cyberjaya Cyberjaya Malaysia

**Keywords:** coronavirus, mobile medical app, self-care, mHealth, health education, app, COVID-19, content analysis

## Abstract

**Background:**

Mobile health (mHealth) app use is a major concern because of the possible dissemination of misinformation that could harm the users. Particularly, it can be difficult for health care professionals to recommend a suitable app for coronavirus disease (COVID-19) education and self-monitoring purposes.

**Objective:**

This study aims to analyze and evaluate the contents as well as features of COVID-19 mobile apps. The findings are instrumental in helping health care professionals to identify suitable mobile apps for COVID-19 self-monitoring and education. The results of the mobile apps’ assessment could potentially help mobile app developers improve or modify their existing mobile app designs to achieve optimal outcomes.

**Methods:**

The search for the mHealth apps available in the android-based Play Store and the iOS-based App Store was conducted between April 18 and May 5, 2020. The region of the App Store where we performed the search was the United States, and a virtual private network app was used to locate and access COVID-19 mobile apps from all countries on the Google Play Store. The inclusion criteria were apps that are related to COVID-19 with no restriction in language type. The basic features assessment criteria used for comparison were the requirement for free subscription, internet connection, education or advisory content, size of the app, ability to export data, and automated data entry. The functionality of the apps was assessed according to knowledge (information on COVID-19), tracing or mapping of COVID-19 cases, home monitoring surveillance, online consultation with a health authority, and official apps run by health authorities.

**Results:**

Of the 223 COVID-19–related mobile apps, only 30 (19.9%) found in the App Store and 28 (44.4%) in the Play Store matched the inclusion criteria. In the basic features assessment, most App Store (10/30, 33.3%) and Play Store (10/28, 35.7%) apps scored 4 out of 7 points. Meanwhile, the outcome of the functionality assessment for most App Store apps (13/30, 43.3%) was a score of 3 compared to android-based apps (10/28, 35.7%), which scored 2 (out of the maximum 5 points). Evaluation of the basic functions showed that 75.0% (n=36) of the 48 included mobile apps do not require a subscription, 56.3% (n=27) provide symptom advice, and 41.7% (n=20) have educational content. In terms of the specific functions, more than half of the included mobile apps are official mobile apps maintained by a health authority for COVID-19 information provision. Around 37.5% (n=18) and 31.3% (n=15) of the mobile apps have tracing or mapping and home monitoring surveillance functions, respectively, with only 17% (n=8) of the mobile apps equipped with an online consultation function.

**Conclusions:**

Most iOS-based apps incorporate infographic mapping of COVID-19 cases, while most android-based apps incorporate home monitoring surveillance features instead of providing focused educational content on COVID-19. It is important to evaluate the contents and features of COVID-19 mobile apps to guide users in choosing a suitable mobile app based on their requirements.

## Introduction

The coronavirus disease (COVID-19) caused by severe acute respiratory syndrome coronavirus 2 (SARS-CoV-2) has received global attention. At the time of writing (April 2020), the number of confirmed cases across the world continues to rise. The World Health Organization announced COVID-19 as a pandemic on March 11, 2020 [1]. Although the SARS-CoV-2 pandemic has had a significant impact in terms of lockdown and mortality, the public has become more eager to obtain information about the spread of the infection [2]. Their efforts in keeping themselves up-to-date with the latest information about COVID-19 could involve existing technologies such as watching the national bulletin on the television or listening to the news on the radio. However, a majority of people may not like the idea of waiting for a live broadcast at a fixed schedule. Reading digital news articles and scrolling through reliable official websites seem to be the main option for tech-savvy individuals [3].

This opens up a golden opportunity for web or mobile medical app developers to create a platform for the public to provide them with the information they are looking for. With the advancement of mobile software and technology, mobile apps have become an important element in our daily life [4]. The use of mobile technology and devices has been found to be successful in the health care setting [5-8]. The term “mobile Health” (mHealth) has been used to describe any health care practice that is supported by mobile devices [9]. For instance, an mHealth app may help health care professionals in treating clinical diseases and educating patients on self-monitoring of the disease as well as reinforcing treatment adherence [10,11]. The use of mHealth apps has made health care and health information easily accessible [12]. Furthermore, the use of mHealth apps at the user’s convenience also helps to reduce the frequency of unnecessary hospital visits by stable patients, thus reducing the mobility of patients who are immunocompromised to high-risk areas [13,14].

The implementation of strategic features in mHealth that can help in diagnosis or symptom reporting has great potential in the management of infections. Additionally, the integration of relevant epidemiological data and geographical information of transmittable disease prevalence in a region will allow the tracing of cases, which can be used as an effective tool to control the spread of infection [15]. It is more effective to deliver health-related information through mHealth apps, as information can be exchanged rapidly and updated dynamically [10]. Mobile apps can potentially prevent the occurrence of a particular disease, as exchanged texts through a mobile app can promote communication, storage of information, and message delivery that drives users to make healthy lifestyle changes [11,16].

Recently the US Food and Drug Administration issued guidance and policy for mHealth apps to ensure their safety and effectiveness [17]. Meanwhile, other challenges revolve around information-sharing and transparency of services offered that could compromise the privacy of the app’s user [18,19]. mHealth app use is also a major concern among health care professionals because of the possible dissemination of misinformation that could harm the users or readers, as some information and services provided are not aligned with medical guidelines [20].

This study aims to analyze and evaluate the contents as well as features of COVID-19 mobile apps. The findings are instrumental in helping health care professionals to identify suitable mobile apps for COVID-19 self-monitoring and education. The results of the mobile apps assessment can potentially help mobile app developers improve or modify their existing mobile app designs to achieve optimal outcomes.

## Methods

We performed a content analysis, comparison, and functionality assessment of selected mobile apps for COVID-19. First, a search for COVID-19 mobile apps was performed in two digital platforms: the App Store on the Apple iPhone 8 Plus and the Google Play Store on Oppo R9s and Vivo V9 smartphones. The search was conducted from April 18, 2020, to May 5, 2020. The region of the App Store where we performed the search was the United States, while a virtual private network (VPN) app named Touch VPN was used to locate and access COVID-19 mobile apps from all countries on the Google Play Store. The inclusion criteria to obtain relevant mHealth apps included apps launched for smartphone users and apps that are related to COVID-19 with no restriction in language type. The exclusion criteria include mobile apps that are launched on other devices such as iPads, tablets, and laptops; apps designed to provide quarantined users with their grocery or pharmacy supplies in response to containing the virus; and entrepreneurship apps designed to collect funds in support of organizations affected by COVID-19.

**Figure 1:**
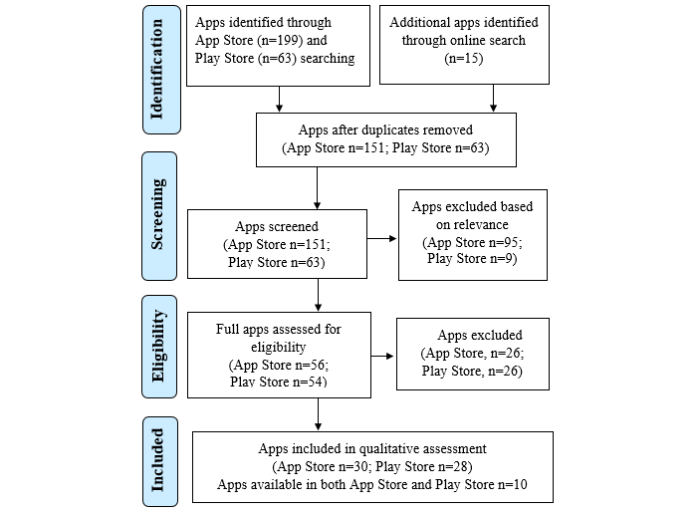
Selection process of mobile health apps in Apple's App Store and the Google Play Store.

The keywords “Covid19,” “Coronavirus,” “Corona,” and “COVID-19” were used to find COVID-19 mobile apps in the App Store and the Play Store. To ensure that all relevant mobile apps were included, an online search on Google using the key terms “mobile app,” “mHealth,” “Covid19,” “Coronavirus,” “Corona,” and “COVID-19” was also conducted. All mobile apps were then filtered according to the COVID-19 relevance and were further filtered according to the inclusion and exclusion criteria. The authors are mainly proficient in the English language, so only apps that support an English language user interface were assessed and reviewed. The summaries of the processes involved in selecting the relevant mobile apps from the App Store and Play Store are illustrated in Figure 1.

The included mobile apps were assessed based on their basic features and functionalities. The basic features were modified from the outline of developed classification of mHealth apps evaluation criteria proposed by Nouri et al [21] and in the literature [11,16,22]. The included seven basic features were (1) no internet requirement, (2) size of app less than 50 MB, (3) no subscription needed (ie, free), (4) educational content (COVID-19 teaching), (5) export data (sharing of user’s data with other platforms), (6) automated data entry (automatic update of data without user interference), and (7) advisory function. Once the assessment of basic features was completed, the researchers convened again to categorize the apps into different groups according to their purpose and functionality, by reading the summary and explanation given by the developers of each included app. The categorized five functionalities of mobile apps were (1) knowledge (information on COVID-19), (2) tracing or mapping of COVID-19 cases, (3) home monitoring surveillance, (4) online consultation with a health authority, and (5) official mobile apps run by a health authority.

The basic features of all included mobile apps were screened individually by three researchers. Any disagreement was discussed until consensus was achieved. The full content of the included mobile apps were then individually examined by the same researchers. Any ambiguity was resolved by two senior researchers to confirm the functionality classification of all included mobile apps. One point was assigned to items that were fully satisfied. No point was given for each item that was partly satisfied or did not apply. There was a maximum of 7 and 5 points for the basic features and functionalities, respectively. Descriptive statistics (frequencies) were used to describe the characteristics of the apps according to the basic features and functionalities.

## Results

The keywords used to search for mobile apps related to COVID-19 in Apple’s App Store and the Google Play Store gave a total of 151 and 63 apps, respectively. The apps were filtered according to the inclusion and exclusion criteria, giving a total of 30 mobile apps from the App Store and 28 mobile apps from the Play Store. The available mobile apps were grouped according to universal COVID-19 apps, country-specific apps, and non-English language apps, as illustrated in Multimedia Appendix 1 for the Apple App Store and Multimedia Appendix 2 for the Google Play Store.

Out of the total number of mobile apps available with relevance to COVID-19, only 30 out of 151 (19.9%) that were found in the Apple App Store and 28 out of 63 (44.4%) in the Google Play Store were assessed. Selected mobile apps were assessed according to their basic features (Tables 1-3) and functionality (Tables 4-6). The results of the assessment follow a scoring system whereby the app was given a score of one for every criterion that it satisfied.

**Table 1:** Basic features assessment of mobile medical apps (iOS and android-based).

No		Name of mobile apps	No internet requirement	Size of app <50 MB	No subscription requirement (ie, free)	Educational content	Export data	Automated data entry	Advisory	Total score
**Universal COVID-19^a^ apps**										
	1	COVID Symptom Tracker	N/A^b^	✓	✓	N/A	✓	✓	✓	5
**Country-specific apps**										
	2	BC COVID-19	N/A	✓	✓	✓	N/A	✓	✓	5
	3	Canada COVID-19	N/A	✓	✓	✓	N/A	✓	✓	5
	4	Coronavirus Australia	N/A	✓	✓	✓	✓	N/A	✓	5
	5	COVA^c^ Punjab	N/A	✓	N/A	—^d^	✓	✓	✓	4
	6	HSE^e^ COVID-19	N/A	✓	N/A	N/A	✓	✓	N/A	3
	7	NCOVI	—	✓	✓	✓	—	—	✓	4
	8	TraceTogether	N/A	✓	✓	N/A	✓	✓	✓	5
	9	자가격리자 안전보호 (Self-Isolator Safety & Protection)	—	✓	✓	✓	—	—	✓	4
	10	자가격리자 전담곤무원 (Self-isolating Government Officials)	—	✓	✓	✓	—	—	✓	4

^a^COVID-19: coronavirus disease.

^b^N/A: not applicable.

^c^COVA: Corona Virus Alert.

^d^Absence of information regarding the feature in the app.

^e^HSE: Health Service Executive.

**Table 2:** Basic features assessment of mobile medical apps (iOS-based only).

No		Name of mobile apps	No internet requirement	Size of app <50 MB	No subscription requirement (ie, free)	Educational content	Export data	Automated data entry	Advisory	Total score
**Universal COVID-19^a^ apps**										
	1	APPLE COVID-19	✓	✓	✓	✓	N/A^b^	N/A	N/A	4
	2	CDC^c^	N/A	✓	✓	✓	N/A	N/A	N/A	3
	3	CoronaFACTS	N/A	✓	✓	✓	N/A	✓	N/A	4
	4	Corona Checker	N/A	✓	✓	N/A	N/A	N/A	✓	3
	5	COVID-19!	N/A	N/A	✓	✓	N/A	N/A	✓	3
	6	HEALTHLYNKED COVID-19 TRACKER	N/A	✓	✓	✓	N/A	N/A	✓	4
	7	RELIEF CENTRAL	N/A	✓	✓	✓	N/A	✓	✓	5
	8	Patient Sphere COVID-19	N/A	✓	✓	N/A	✓	N/A	N/A	3
	9	PreMedicus	—^d^	N/A	N/A	N/A	✓	N/A	✓	2
	10	Mobile Angel Cancer Telemed	N/A	N/A	✓	N/A	✓	N/A	N/A	2
**Country-specific apps**										
	11	BMC Combat Covid19	N/A	✓	✓	N/A	N/A	N/A	N/A	2
	12	Corona-Care	N/A	N/A	N/A	N/A	✓	✓	✓	3
	13	COVID-19 Gov PK^e^	N/A	✓	✓	✓	N/A	✓	✓	5
	14	Covidom Patient	N/A	N/A	N/A	N/A	✓	N/A	✓	2
	15	COVI QATAR	N/A	✓	✓	✓	N/A	N/A	✓	4
	16	COVID-19 UAE^f^	N/A	N/A	✓	✓	N/A	✓	✓	4
	17	CUREiTT	N/A	N/A	N/A	N/A	✓	✓	N/A	2
	18	NJ COVID 19	N/A	✓	✓	✓	N/A	N/A	✓	4
	19	STOP COVID19 CAT	N/A	✓	N/A	—	✓	—	—	2
	20	Tarussud	N/A	✓	✓	✓	N/A	✓	✓	5

^a^COVID-19: coronavirus disease.

^b^N/A: not applicable.

^c^CDC: Centers for Disease Control and Prevention.

^d^Absence of information regarding the feature in the app.

^e^PK: Pakistan.

^f^UAE: United Arab Emirates.

**Table 3:** Basic features assessment of mobile medical apps (android-based only)

No		Name of mobile apps	No internet requirement	Size of app <50 MB	No subscription requirement (ie, free)	Educational content	Exported data	Automated data entry	Advisory	Total score
**Universal COVID-19^a^ apps**										
	1	Test Yourself Goa	N/A^b^	✓	✓	N/A	✓	N/A	✓	4
**Country-specific apps**										
	2	Aarogya Setu	N/A	✓	✓	N/A	✓	✓	✓	5
	3	CoBuddy – Covid19 Tool	N/A	✓	N/A	N/A	✓	✓	N/A	3
	4	Corona Watch	N/A	✓	✓	N/A	N/A	✓	✓	4
	5	COVI	N/A	✓	✓	✓	N/A	✓	✓	5
	6	Covid-19	N/A	✓	N/A	N/A	✓	✓	N/A	3
	7	COVID-19 NI^c^	✓	✓	✓	✓	N/A	✓	✓	6
	8	COVID19 Feedback	N/A	✓	✓	N/A	✓	✓	N/A	4
	9	COVID-19 Quarantine Monitor Tamil Nadu (official)	N/A	✓	✓	N/A	✓	✓	N/A	4
	10	COVID-19 West Bengal Government	N/A	✓	✓	N/A	✓	✓	N/A	4
	11	GCC^d^ -Corona Monitoring	N/A	✓	✓	N/A	N/A	✓	N/A	3
	12	GoK – Direct Kerala	N/A	✓	✓	N/A	N/A	N/A	N/A	2
	13	Home Quarantine (Kwarantanna domowa)	N/A	N/A	✓	N/A	✓	✓	N/A	3
	14	Mahakavach	N/A	✓	N/A	N/A	✓	✓	—^e^	3
	15	MP^f^ COVID RESPONSE APP	N/A	✓	✓	—	✓	—	—	3
	16	Quarantine Watch	N/A	✓	N/A	—	✓	—	—	2
	17	StayHomeSafe	✓	✓	N/A	—	✓	✓	—	4
	18	Test Yourself Puducherry	N/A	✓	✓	✓	✓	✓	✓	6

^a^COVID-19: coronavirus disease.

^b^N/A: not applicable.

^c^NI: North Ireland.

^d^GCC: Greater Chennai Corporation.

^e^Absence of information regarding the feature in the app.

^f^MP: National Health Mission.

**Table 4:** Functionality assessment of mobile medical apps (iOS and android-based).

No		Name of mobile apps	Knowledge	Tracing/mapping of COVID-19^a^ cases	Home monitoring surveillance	Online consultation with health authority	Official mobile app maintained by health authority	Total score
**Universal COVID-19 apps**								
	1	COVID Symptom Tracker	✓	✓	N/A^b^	N/A	✓	3
**Country-specific apps**								
	2	BC COVID-19	✓	N/A	N/A	N/A	✓	2
	3	Canada COVID-19	✓	✓	N/A	N/A	✓	3
	4	Coronavirus Australia	✓	✓	N/A	N/A	✓	3
	5	COVA^c^ Punjab	✓	✓	—^d^	✓	✓	4
	6	HSE^e^ COVID-19	✓	N/A	✓	N/A	✓	3
	7	NCOVI	✓	✓	—	—	✓	3
	8	TraceTogether	✓	✓	N/A	✓	✓	4
	9	자가격리자 안전보호 (Self-Isolator Safety & Protection)	✓	—	✓	—	✓	3
	10	자가격리자 전담곤무원 (Self-isolating Government Officials)	✓	—	✓	—	✓	3

^a^COVID-19: coronavirus disease.

^b^N/A: not applicable.

^c^COVA: Corona Virus Alert.

^d^Absence of information regarding the feature in the app.

^e^HSE: Health Service Executive.

**Table 5:** Functionality assessment of mobile medical apps (iOS-based only).

No		Name of mobile apps	Knowledge	Tracing/mapping of COVID-19^a^ cases	Home monitoring surveillance	Online consultation with health authority	Official mobile app maintained by health authority	Total score
**Universal COVID-19 apps**								
	1	Apple COVID-19	✓	N/A^b^	N/A	N/A	✓	2
	2	CDC^c^	✓	N/A	N/A	N/A	✓	2
	3	CoronaFACTS	✓	✓	N/A	N/A	✓	3
	4	Corona Checker	N/A	N/A	N/A	N/A	N/A	0
	5	COVID-19!	✓	✓	N/A	N/A	N/A	2
	6	HEALTHLYNKED COVID-19 TRACKER	✓	✓	N/A	N/A	✓	3
	7	RELIEF CENTRAL	✓	✓	N/A	N/A	N/A	2
	8	Patient Sphere for COVID-19	N/A	N/A	✓	N/A	N/A	1
	9	PreMedicus ER	✓	N/A	N/A	✓	N/A	2
	10	Mobile Angel Telemed	N/A	N/A	✓	✓	✓	3
**Country-specific apps**								
	11	BMC Combat Covid19	N/A	N/A	✓	✓	✓	3
	12	Corona-Care	✓	✓	N/A	N/A	✓	3
	13	COVID-19 Gov PK^d^	✓	N/A	N/A	N/A	✓	2
	14	Covidom Patient	N/A	N/A	N/A	✓	✓	2
	15	COVI QATAR	✓	N/A	N/A	N/A	✓	2
	16	COVID-19 UAE^e^	✓	N/A	N/A	N/A	✓	2
	17	CUREiTT	✓	N/A	N/A	N/A	N/A	1
	18	NJ COVID 19	✓	✓	N/A	N/A	✓	3
	19	STOP COVID19 CAT	—^f^	✓	—	—	✓	2
	20	Tarassud	✓	✓	N/A	✓	✓	4

^a^COVID-19: coronavirus disease.

^b^N/A: not applicable.

^c^CDC: Centers for Disease Control and Prevention.

^d^PK: Pakistan.

^e^UAE: United Arab Emirates.

^f^Absence of information regarding the feature in the app.

**Table 6:** Functionality assessment of mobile medical apps (android-based only).

No			Name of mobile apps		Knowledge		Tracing/mapping of COVID-19^a^ cases		Home monitoring surveillance		Online consultation with health authority		Official mobile app maintained by health authority		Total score
**Universal COVID-19 apps**															
	1	Test Yourself Goa		✓		N/A^b^		N/A		N/A		✓		2	
**Country-specific apps**															
	2	Aarogya Setu		N/A		✓		N/A		N/A		✓		2	
	3	CoBuddy – Covid19 Tool		N/A		N/A		✓		N/A		N/A		1	
	4	Corona Watch		✓		✓		N/A		N/A		N/A		2	
	5	COVI		✓		N/A		N/A		N/A		✓		2	
	6	Covid-19		N/A		N/A		✓		✓		✓		3	
	7	COVID-19 NI^c^		✓		N/A		N/A		N/A		✓		2	
	8	COVID19 Feedback		N/A		N/A		N/A		N/A		N/A		0	
	9	COVID-19 Quarantine Monitor Tamil Nadu (official)		N/A		N/A		✓		N/A		N/A		1	
	10	COVID-19 West Bengal Government		N/A		N/A		✓		N/A		N/A		1	
	11	GCC^d^ -Corona Monitoring		N/A		✓		✓		N/A		N/A		2	
	12	GoK – Direct Kerala		✓		N/A		N/A		N/A		✓		2	
	13	Home Quarantine (Kwarantanna domowa)		✓		N/A		✓		N/A		✓		3	
	14	Mahakavach		✓		✓		✓		—^e^		✓		4	
	15	MP^f^ COVID RESPONSE APP		—		—		—		—		—		—	
	16	Quarantine Watch		—		—		✓		—		✓		2	
	17	StayHomeSafe		—		—		✓		—		✓		2	
	18	Test Yourself Puducherry		✓		N/A		N/A		N/A		✓		2	

^a^COVID-19: coronavirus disease.

^b^N/A: not applicable.

^c^NI: North Ireland.

^d^GCC: Greater Chennai Corporation.

^e^Absence of information regarding the feature in the app.

^f^MP: National Health Mission.

**Figure 2:**
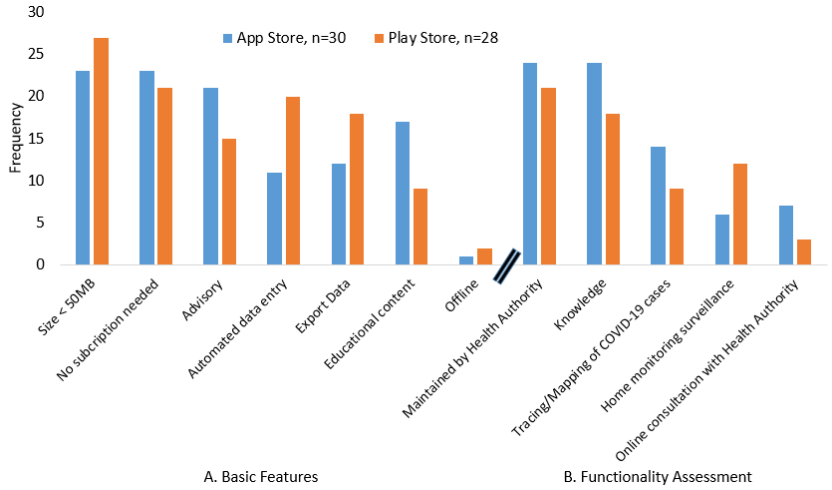
Assessment of iOS and android-based mobile apps (A: Basic features; B: Functionality assessment). COVID-19: coronavirus disease.

The criteria assessed under basic features include the requirement of internet connectivity to use the app, storage capacity, subscription requirement, educational content, ability to export data, automated data entry support, and an advisory feature. According to the results obtained from the assessment of basic features of mobile apps as illustrated in Figure 2A, a majority of the apps from the App Store (29/30, 96.7%) and Google Play (26/28, 92.9%) require internet connectivity to be accessed. There is a higher proportion of mobile apps from Apple (17-23/30, 56.7%-76.7%) that can be accessed without any subscription while providing educational content and advice than Android mobile apps (9-21/28, 32.1%-75.0%). Meanwhile, there are slightly more COVID-19 mobile apps (18-27/28, 64.3%-96.4%) from Google that are less than 50MB in capacity with the ability to export data and allow automated data entry in comparison to Apple (11-23/30, 36.7%-76.7%).

Apart from assessing the basic features, the mobile apps were also assessed based on their functionality as illustrated in Figure 2B. The criteria assessed under functionality includes the availability of COVID-19–related information, tracing or mapping of COVID-19 cases, home monitoring surveillance, online consultation with a health authority, and whether or not the mobile apps are maintained by a health authority. Most of the mHealth apps (7-24/30, 23.3%-80.0%) in the App Store on an iPhone provide better functionality than mHealth apps in the Play Store on an Android smartphone. However, a higher proportion of Android mobile apps (12/28, 42.9%) offer home monitoring surveillance related to COVID-19 than Apple (6/30, 20.0%).

**Figure 3:**
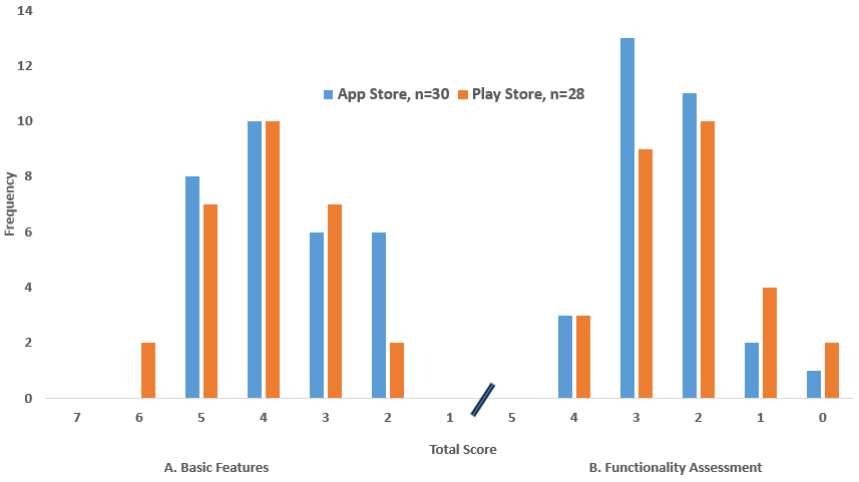
Total assessment score of iOS and android-based mobile apps (A: Basic features; B: Functionality assessment).

When assessing the basic features of the mobile apps, none of the apps from the Apple App Store scored 1, 6, or 7, as shown in Figure 3A. Out of the total 30 apps, there were 6 apps (20.0%) that scored two and three, 10 apps (33.3%) that scored four, and 8 apps (26.7%) that scored five. Meanwhile, for apps downloaded from the Google Play Store, none of the apps scored 1 or 7. Out of the total 28 apps, there were 2 apps (7.1%) that scored two, 7 apps (25.0%) that scored three, 10 apps (35.7%) that scored four, 7 apps (25.0%) that scored five, and the remaining 2 apps (7.1%) scored six. In Figure 3B, when assessing the functionality of the mobile apps, none of the apps from the Apple App Store scored 5. There was 1 app (3.3%) that scored zero, 2 apps (6.7%) that scored one, 11 apps (36.7%) scored two, 3 apps (10%) that scored four, and the majority (n=13, 43.3%) scored three. From the Google Play Store, none of the apps scored 5. There were 2 apps (7.1%) that scored zero, 4 apps (14.3%) that scored one, 9 apps (32.1%) that scored three, 3 apps (10.7%) that scored four, and the majority (n=10, 35.7%) scored two.

## Discussion

### Principal Findings

According to the assessment that has been conducted, the mobile apps vary in terms of basic features and functionality. Basic features consist of trivial characteristics that may or may not be of significant importance to users who would like to use COVID-19 mobile apps for COVID-19–related education or self-monitoring purposes with no existing issue for internet access or low mobile storage capacity. Another assessment was conducted based on the advanced features found in the apps. The advanced features touch on the type of content that the app offers on COVID-19, which were used as a measure of the quality of the mobile app. In terms of the basic features, most of the apps from the App Store and the Play Store require no subscriptions and have a storage size of less than 50 MB. There are some apps that need to be paid for, as they need the revenue for advertisements and data mining [23].

People could mistakenly assume that paid mHealth apps have better content or app design. Their value should be assessed in relation to their overall strengths and limitations [21,24]. It is recommended for mHealth apps to have a small storage size, as taking up a lot of phone storage space can result in reduced performance of the mobile phone [25]. Moreover, the App Store offers more apps that provide educational content and advice on COVID-19 than Google Play. This shows the potential of mHealth apps in transforming the delivery of health care services [26]. On the other hand, there are more mobile apps from the Google Play Store that enable data to be exported and offer automated data entry in comparison to the App Store. The availability of data export will allow users to share health reports with their health care providers. Automated data entry can provide greater efficiency in inputting data to the app system while streamlining the user’s experience [27,28].

There were 2 COVID-19 mobile apps from that Play Store that had the highest score of six in the assessment of basic features (COVID-19 NI [Northern Ireland] and Test Yourself Puducherry) and 8 COVID-19 mobile apps from the App Store that had the highest score of five (BC COVID-19, COVID Symptom Tracker, Canada COVID-19, Coronavirus Australia, COVID-19 Gov PK [Pakistan], Relief Central, Tarassud, and TraceTogether).

When assessing the functionality of the mobile apps, there were 3 COVID-19 mobile apps from the App Store and the Play Store that had the highest score of four: COVA (coronavirus alert) Punjab and TraceTogether from both the App Store and the Play Store, Tarassud from the App Store, and Mavakavach from the Play Store.

### Detailed Review of Mobile Apps in the Apple App Store

Among the apps meant for universal use with an Apple device, APPLE COVID-19 app ranked first in the category of health and fitness with the second-highest user rating of 4.4, and HEALTHLYNKED COVID-19 TRACKER ranked second in the medical category with a slightly higher user rating of 4.6 (rated by thousands of mobile users). Though other apps rated more than 4.5, including CoronaFACTS, Corona Checker, and COVID-19!, there is no strong support that the operating performance of these apps is better than APPLE COVID-19 or HEALTHLYNKED COVID-19 TRACKER because of the low number of users who rated the apps.

APPLE COVID-19 and HEALTHLYNKED COVID-19 TRACKER achieved a score of 4 in the basic feature assessment. One main difference is the ability to access APPLE COVID-19 without requiring any internet or data use, unlike HEALTHYLYNKED COVID-19 TRACKER. However, HEALTHLYNKED COVID-19 TRACKER allows mapping of COVID-19 cases to provide users with an up-to-date statistic on the number of COVID-19 infections worldwide; a feature that APPLE COVID-19 does not offer. Both apps are run by the health authorities in the United States as APPLE COVID-19 was developed in collaboration with the Centers for Disease Control and Prevention (CDC), while HEALTHLYNKED COVID-19 TRACKER was developed by a company of medical professionals. It is recommended for a developer of health-related apps to collaborate with health care professionals to provide up-to-date content that can be easily trusted by the public [29].

Both APPLE COVID-19 and Relief Central apps scored 2 in the functionality assessment but Relief Central scored an extra point (5) than APPLE COVID-19 (4) in the basic feature assessment, as it supports automated data entry. However, disseminated information in the Relief Central app is not aimed for the general public but those with a background in the medical field, such as health care professionals and students (eg, pharmacy, medical, nursing, and allied health), as the profession of the user is inquired before they can proceed further in using the app. Thus, the content in this app cannot be accessed for the public’s perusal.

Both CoronaFACTS and HEALTHLYNKED COVID-19 TRACKER scored the same in both assessments: 4 points in the basic feature assessment and 3 points in the functionality assessment. Both offer the same advanced features of providing information on COVID-19 and mapping of COVID-19 cases, and are maintained by the health authority of their country. The differences between the two apps is that there is no advisory content in CoronaFACTS, unlike HEALTHLYNKED COVID-19 TRACKER, and CoronaFACTS is another app that supports automated data entry, which HEALTHYLYNKED COVID-19 does not support. CoronaFACTS functions by collecting COVID-19–related newspaper articles from trusted sources based on the region chosen by the user, and directs the user to the official site of the article.

Country-specific apps from both stores were also assessed. From the Apple App Store, apps named COVID-19 PK Gov and Tarassud scored the highest (5) in their basic features assessment, with limitations including that users have to be online to operate the app and the absence of a data exportation feature. Other limiting factors of COVID-19 Gov PK in its content are the absence of mapping of COVID-19 cases, home monitoring surveillance, and online consultation with a health authority. Tarassud has better functionality, as it scored 4 out of 5 in the functionality assessment, with only one limitation whereby there is an absence of a home monitoring surveillance feature. Tarassud also includes information on the updated number of cases around the world with guidelines on COVID-19 for those in isolation and quarantine. Of note, using electronically collected influenza data at a Swedish county as an example, the prediction of the influenza virus activity using retrospective surveillance data was successfully integrated into the local health care management system [30].

### Detailed Review of Mobile Apps in the Google Play Store

An app named Test Yourself Goa is available for universal use on an Android device. It is an app used to determine if an individual is at high risk of getting COVID-19 based on the symptom-checking feature. After the completion of the symptom-checking test, a piece of advice is given that is aligned with the guidelines proposed by the CDC. For patients who are just learning to manage their disease, mHealth can be of help thanks to its ability to provide advice based on the aggregation of data [31]. In our assessment, it satisfied almost all of the favorable basic features except that it requires an internet connection, has no educational content on the disease, and no automated data entry feature. Test Yourself Goa also allows the health authorities to collect the user’s details such as full name, mobile number, zip code, and address. In the functionality assessment, this mobile app only scored 2 out of 5, as it provides information on COVID-19 and is an official app maintained by health authorities.

Meanwhile, among country-specific apps from the Play Store, COVID-19 NI and Test Yourself Puducherry scored the highest in their basic feature assessment with scores of 6 each. The differences between the two apps are that COVID-19 NI does not require internet connectivity, and there is an absence of data exportation in comparison to Test Yourself Puducherry. Both apps also got the same score in their functionality assessment with a considerably low score of 2, proving that they contain COVID-19 information and are maintained by a health authority. The mobile apps can provide disease-specific information to patients, the general public, or health care professionals, which can raise the awareness of users to the signs, causes, and effects of the disease with a view of containing the disease [32].

### Detailed Review of Mobile Apps Available in Both Apple App Store and Google Play Store

The only app that is available in both the App Store and the Play Store for universal use is COVID Symptom Tracker. This COVID-19 mobile app enables users to report any symptoms daily. The users need to fill in their personal information such as age, sex, height, weight, and postcode. Additionally, the user’s data will be collected by the health authority. Based on the findings in the basic feature assessment, COVID Symptom Tracker requires internet access to operate the app, and it does not provide any educational content. In the functionality assessment, the app scored 3 out of 5 due to the unavailability of several features including home monitoring surveillance and online consultation with a health care professional.

An app named COVA Punjab scored the second-highest in the basic feature assessment with a restriction of the app requiring the internet to work. Most of the mHealth apps in the Apple App Store and the Google Play Store require an internet connection, as it is an important feature for the developers to enable real time data synchronization with the app to prevent the display of outdated information [33,34].

However, the reviewer was not able to access the app, as user registration requires a local phone number. Hence, there is no information about whether or not the app has any educational content on COVID-19. In the functionality assessment, both COVA Punjab and TraceTogether scored 4 out of 5. TraceTogether does not have a home monitoring surveillance feature, and there is no information on the feature in the COVA Punjab app. However, TraceTogether scored more (5) than COVA Punjab (4) in its basic feature assessment with similar limitations. Although TraceTogether is confirmed to have no educational content, there is a lack of information regarding this feature in COVA Punjab.

TraceTogether has not only the highest score in the basic feature assessment but also has the highest score in the functionality assessment in both the App Store and the Play Store. It is an app used mainly for contact tracing of COVID-19 cases in Singapore to enable users to be notified of people who had close contacts with COVID-19 positive cases. Although contact tracing initially can be done manually, the number of confirmed cases of COVID-19 continues to rise across the world, and it has become difficult to do. Therefore, there are a lot of countries that have used different methods of contact tracing [35]. For example, Taiwan has given health institutions access to patients’ travel history and allows relevant authorities to monitor anyone under quarantine by tracking the location of their mobile phone [35]. Meanwhile, in South Korea, the government has maintained a public database of the personal data of patients with COVID-19 including their age, gender, profession, and travel routes [35]. In the case of TraceTogether, if the user is suspected to be infected with COVID-19, the user’s data from the app will also be collected by the health authority for contact tracing purposes. TraceTogether also gives information on the functions of the app for a first time user. As the app is being maintained by the health authority, it also allows users to interact with the health authority in addition to allowing users to upload relevant files such as images and documents.

There are also a number of apps that scored low in the functionality assessment. In the Apple App Store, CoronaChecker was identified as a low-scoring app with a score of 0. CoronaChecker is an app intended to provide suggestions on the requirement of a COVID-19 test for the user. Using artificial intelligence, a conversation is initiated with the user upon opening the app. During the interaction, closed questions are generated to ask the user about any clinical symptoms that they may be manifesting to check for any possible viral infection. Despite the low-functionality offered by this app, it was rated 5 out of 5 by 298 users. This could be an indication that the users were satisfied by the operating performance of the app, as CoronaChecker is only designed for users to confirm their health status and not as an educational tool for COVID-19.

COVID19 Feedback obtained the lowest score (0) in the functionality assessment from the Google Play Store. The mobile app only requests feedback from individuals who have done the coronavirus test in India. The feedback received from the public will be used to improve the efficiency and processes related to the coronavirus test. Hence, COVID19 Feedback does not support any of the advanced features related to COVID-19.

In the Apple App Store, CUREiTT scored the lowest in both assessments. It scored two in the basic assessment, as it is only available in exporting data and it supports automated data entry. The app also collects the user’s personal information such as phone number, year of birth, and gender. Although CUREiTT only scored one in the functionality assessment, it has a unique function of displaying appropriate clinical trials in the geographical area of individuals diagnosed with cancer or coronavirus.

GoK – Direct Kerala app had the lowest score in both assessments for the Google Play Store. The two scores from the basic feature assessment were due to the storage size of less than 50 MB and the absence of a subscription requirement. However, the app does provide national news reports about the government’s actions to manage COVID-19 cases. Moreover, it is an official mobile app maintained by a health authority. Quarantine Watch also has the same score as GoK – Direct Kerala but most of the assessments were not measured, as a national phone number was required for mandatory registration.

### Recommendation on COVID-19 Mobile Apps

Every app has a specific goal and a target audience. We have carefully assessed the available apps, which has shown that only a few apps can be used globally for COVID-19 education and self-monitoring. From the previously mentioned results, a high-quality mobile app that can be globally used is COVID Symptom Tracker, which can be installed on both Apple and Android devices. With the aim of slowing the outbreak of the virus, a symptom tracker is useful in identifying high-risk areas in the country, the speed of viral transmission in an area, and individuals who are at the most risk in relation to their health conditions [36].

Based on our study, Mahakavasch and Home Quarantine are the only two apps from the Google Play Store that contain both important features for users: provision of information related to COVID-19 and home-monitoring features. The home-monitoring features are only available for the purpose of monitoring those in quarantine. Home Quarantine, as the name suggests, is designed for those who were assigned to be quarantined at their homes for 14 days. If the users suspect they have been exposed to COVID-19 during their quarantine, they can simply contact the number stated in the app. Mahakavasch has a similar concept as Home Quarantine but it differs slightly, as the users of Mahakavasch will need to take a self-portrait and upload it in the app to allow their location during their quarantine period to be detected.

### Self-Monitoring Apps

The use of apps on smartphones to conduct self-assessments for COVID-19 can help to notify the user of their health status. It is also known that technology can promote rapid identification of potential COVID-19 cases for timely interventions to be carried out [37]. This is because COVID-19 is a communicable viral disease in which infected people may appear asymptomatic [36]. With the availability of self-monitoring apps, the user can continue to perform their daily activities at home without going to the hospital for a check-up [21]. Other advantages of self-monitoring apps are the ability to observe a patient’s condition at any time, increased efficiency of the health care services with the use of modern technology, and reduced burden of patients who are immobilized who cannot regularly visit a hospital [38].

Altogether from both stores, there were 6 apps that offer COVID-19 home-monitoring features. The 3 apps from the App Store were BMC Combat COVID19, Patient Sphere for Covid-19, and Mobile Angel. BMC Combat Covid19 is the only app that is specific to citizens of British Columbia, while the other 2 apps are aimed at universal use. BMC Combat Covid19 and Mobile Angel Cancer Telemed scored 2, while Patient Sphere for COVID-19 scored 3 in the basic feature assessment. Patient Sphere for Covid-19 functions similarly as a health record system for a registered individual. Registration can be made through email or phone number. It allows users to manually enter their data in the symptom diary after monitoring themselves. The monitoring parameters that can be recorded are related to common symptoms of COVID-19, such as a stuffy nose, cough, shortness of breath, and chills. However, the Mobile Angel app is restricted to patients from a health care facility that have been registered in the app.

The other 3 apps that offer home-monitoring features are from the Play Store: CoBuddy - Covid19 Tool, COVID-19 Quarantine Monitor Tamil Nadu (official), and COVID-19 West Bengal Government. Unfortunately, the apps are only available for use in specific regions. COVID-19 West Bengal Government and COVID-19 Quarantine Monitor Tamil Nadu (official) scored 4 and CoBuddy - Covid19 Tool scored 3 in basic features. COVID-19 Quarantine Monitor Tamil Nadu (official) and COVID-19 West Bengal Government provide similar contents. A toggle switch should be enabled in the apps to allow the user’s daily condition to be monitored.

### Quality Improvement of Mobile Apps

Based on the research, there are several recommendations that mobile app developers can consider to improve their existing COVID-19 apps or create a high-quality COVID-19 mobile app in the future.

First, it is recommended that a health-related app be maintained by a health authority to avoid the spread of misleading information to the public. A collaboration with the health authorities to create a mHealth app can increase the reliability of the app, which will encourage more users to be engaged in its use. Otherwise, the user can be informed of the shared information source provided in the app. Second, to increase the engagement rate of the public with the mobile app, it should also contain background information on COVID-19, guidelines, and preventive measures instead of only a focused feature related to COVID-19 (eg, symptom-tracking feature). Moreover, the app should be available for universal use instead of only for residents in a specific country. Third, it is suggested that the apps should be made available without requiring any payment in both the Apple App Store and the Google Play Store to make them more accessible.

Fourth, including real time or near real time updates of statistical analytics with geographical information of positive cases, recovered cases, and a death toll is highly recommended to allow users to be readily informed about the COVID-19 situation worldwide. Fifth, to ensure that users can safely share their personal details, the app should be secure and able to provide assurance to the user that all shared information is kept confidential.

Furthermore, there are other advanced features that can greatly improve the quality of an mobile app including the addition of a feature that can report crowded places to alert users about areas to be avoided to allow them to practice social distancing, as well as a quarantine attendance status, online consultation with health care professionals, and tracing of the whereabouts of positively infected app users to alert their close contacts to undergo contact tracing for COVID-19. It is also crucial to categorize mobile apps into appropriate categories to enable users to find an app easily and thus improve its user engagement rate.

Many COVID-19 mobile apps are appropriately placed under Health and Fitness and Medical. Examples of categories that do not correspond to COVID-19 mobile apps are reference, news, utilities, and tools, as these terms lack specificity in the content they display. Last, an advanced integration of an mHealth app with a health device that can monitor a user’s health, such as a digital thermometer that can automatically record the user’s body temperature reading in the app, will also enhance the self-monitoring feature of the app.

### Limitations

Several limitations were found throughout the study conducted. First, our findings on the available mobile apps in the Google Play Store were limited, as the search for any COVID-19–related keyword has been disabled by Google to avoid any misinformation on the disease. Second, after this research was completed, it is likely that there will be more updated features in the assessed mobile apps. Moreover, new COVID-19 mobile apps may be launched that could not be included in this review. Third, there are gaps in our research, as some apps were inaccessible to the reviewers. This is due to the strict verification process using either a local phone number, especially for apps designed with country-specific functionalities or restricted access for specific users only. There was also 1 app that required payment before the installation of the app could begin. Therefore, some apps were marked with “—” in the assessment results, which indicated the absence of information regarding a certain feature in the app. Fourth, no usability study has been conducted to test for users’ responses to COVID-19 mobile apps, so the authors could not conduct a systematic review of literature regarding COVID-19 mobile apps.

### Conclusions

It is important to evaluate the contents and features of COVID-19 mobile apps to guide users in choosing a suitable mobile app based on their requirements and help developers to improve the designs of their existing or future mobile apps to further enhance quality. Evaluation of basic functions showed that 75.0% (n=36) of the included 48 mobile apps do not require a subscription, 56.3% (n=27) provide symptom advice, and 41.7% (n=20) have educational content. In terms of the specific functions, more than half of the included mobile apps are official mobile apps maintained by a health authority for COVID-19 information provision. Around 37.5% (n=18) and 31.3% (n=15) of the mobile apps have tracing or mapping and home monitoring surveillance functions, respectively, with only 17% (n=8) of mobile apps equipped with an online consultation function. Quality-wise, 58.3% (n=28) of the included mobile apps scored 4 points and above out of the maximum 7, proving that during the time constraint of a few weeks, the mobile app developers did not manage to create a fully comprehensive mobile app. Our study paves the way for future work to determine the role of mobile apps in controlling the rate of COVID-19 transmission.

## Appendix

Multimedia Appendix 1 Characteristic of mobile medical apps (iOS-based).

Multimedia Appendix 2 Characteristic of mobile medical apps (android-based).

## Abbreviations

CDC: Centers for Disease Control and Prevention
COVA: coronavirus alert
COVID-19: coronavirus disease
mHealth: mobile health
NI: Northern Ireland
PK: Pakistan
SARS-CoV-2: severe acute respiratory syndrome coronavirus 2
VPN: virtual private network
