# Supplementary material for: Mobile Health Apps on COVID-19 Launched in the Early Days of the Pandemic: Content Analysis and Review
Source: JMIR Mhealth Uhealth. 2020 Sep 16;8(9):e19796. doi: 10.2196/19796 (PMC7505686; doi:10.2196/19796)
Supplement: Multimedia Appendix 1 [file mhealth_v8i9e19796_app1.docx]

*Research paper*

**Content Analysis and Review of Mobile Health Applications on COVID-19**

Long Chiau Ming*^§1^, Noorazrina Untong^§1^, Nur Amalina Aliudin^§1^, Norliza Osili^§1^, Nurolaini Kifli^1^, Ching Siang Tan^2^, Khang Wen Goh^3^, Pit Wei Ng^4^, Yaser Mohammed Al-Worafi^5,6^, Kah Seng Lee^7^, Poh Hui Goh*^1^

^1^PAPRSB Institute of Health Sciences, Universiti Brunei Darussalam, Gadong, Brunei Darussalam

^2^School of Pharmacy, KPJ Healthcare University College, Nilai, Negeri Sembilan, Malaysia

^3^Faculty of Science and Technology, Quest International University Perak, Ipoh, Perak, Malaysia

^4^Department of Pharmacy, National University Health System, City of Singapore, Singapore

^5^College of Pharmacy, University of Science and Technology, Sana’a, Yemen

^6^College of Pharmacy, University of Science and Technology of Fujairah, Fujairah, UAE

^7^Faculty of Pharmacy, University of Cyberjaya, Cyberjaya, Selangor, Malaysia

^§^equal contribution

*Corresponding authors

Long Chiau Ming, PhD; Poh Hui Goh, PhD

PAPRSB Institute of Health Sciences, Universiti Brunei Darussalam, Jalan Tungku Link Gadong BE1410, Gadong, Brunei Darussalam

long.ming@ubd.edu.bn; pohhui.goh@ubd.edu.bn

**Table 1. Characteristic of mobile medical apps (iOS-based)**

| **Table 1a. Universal COVID-19 apps** | | | | | | | | | | | | | | | | | | | |
| --- | --- | --- | --- | --- | --- | --- | --- | --- | --- | --- | --- | --- | --- | --- | --- | --- | --- | --- | --- |
| **No.** | | **Name of**  **mobile**  **applications** | **Name of developer (Company/**  **Organisation)** | | **Logo** | | **Country** | | **Size** | | **User rating** | **Classification** | | **Category** | |  | | **No. of downloads** | |
| **1** | | **Apple COVID-19** | Apple Inc, Federal Emergency  Management & Centers for Disease Control and Prevention | | 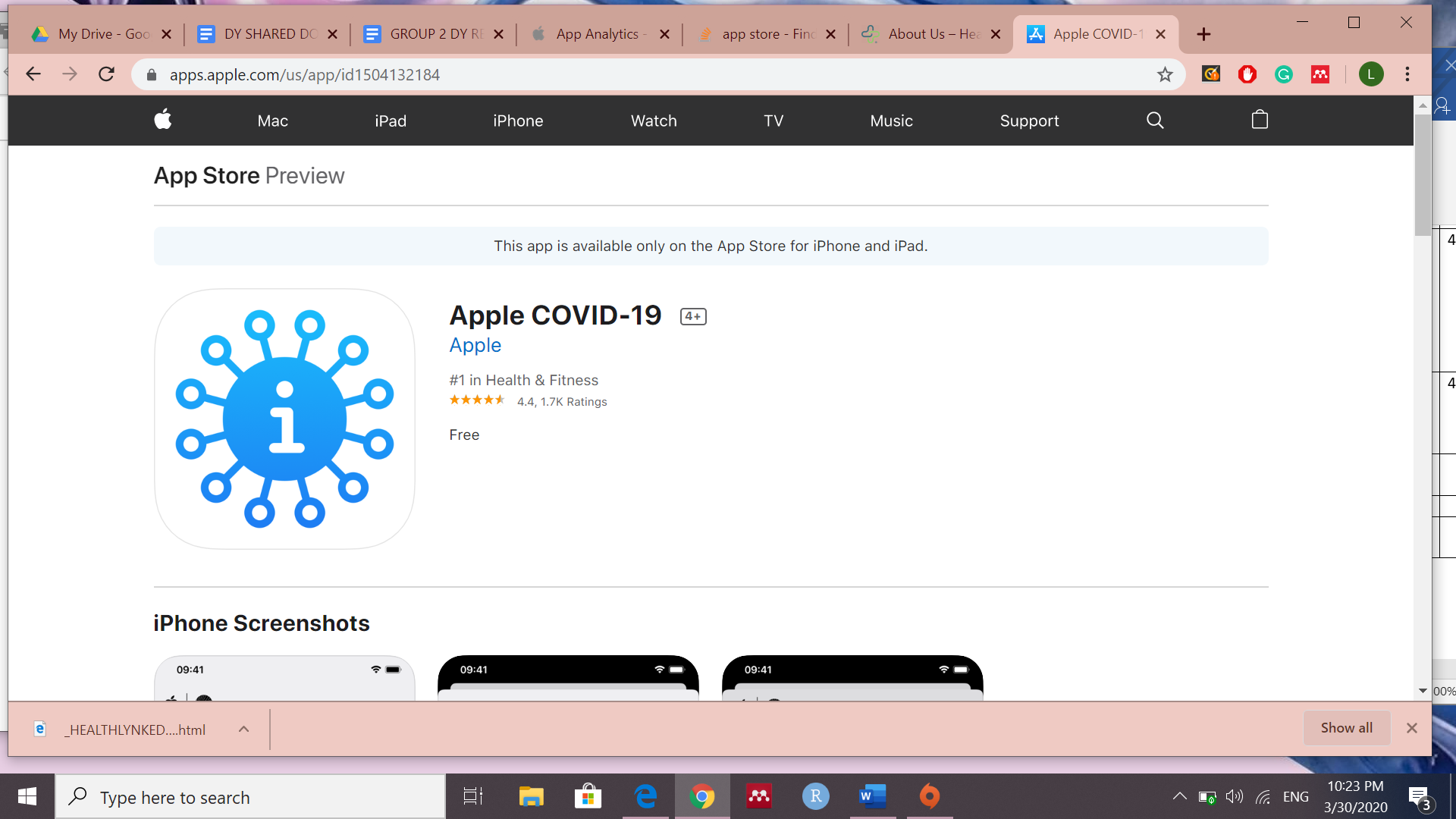 | | United States | | 6.1 MB | | 4.4  (n= 1.7K) | 4+ | | #1  Health & Fitness | |  | | N/A | |
| **2** | | **CDC** | The Centers for Disease Control and Prevention | | 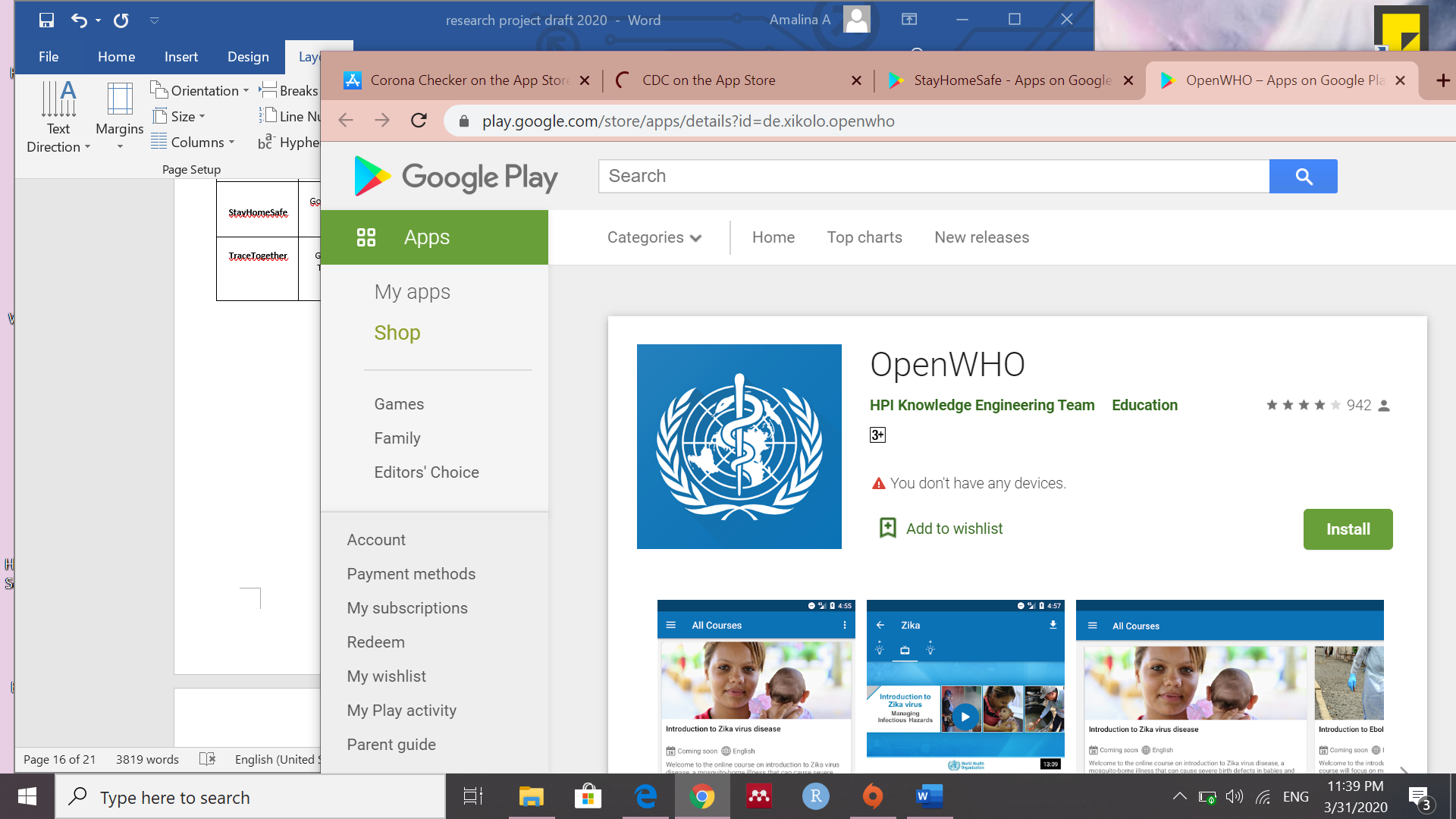 | | United States | | 45.6 MB | | 2.9  (n= 169) | 12+ | | #35 Health & Fitness | |  | | N/A | |
| **3** | | **CoronaFACTS** | Trusted Medical LLC & National Coordination Centre | | 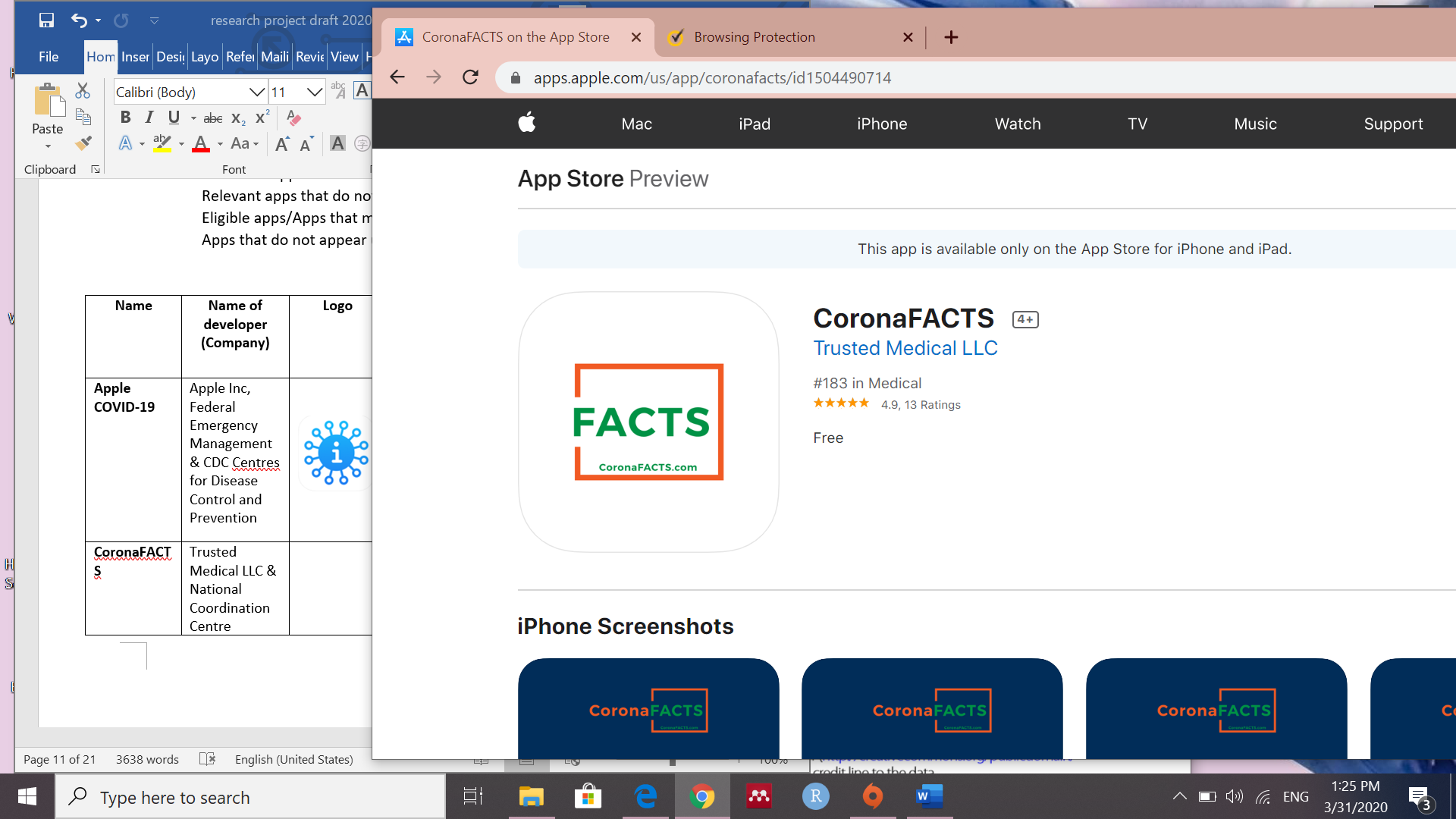 | | United States | | 31.2 MB | | 4.9  (n= 10) | 4+ | | #183 Medical | |  | | N/A | |
| **4** | | **Corona Checker** | Open Med Inc. | | 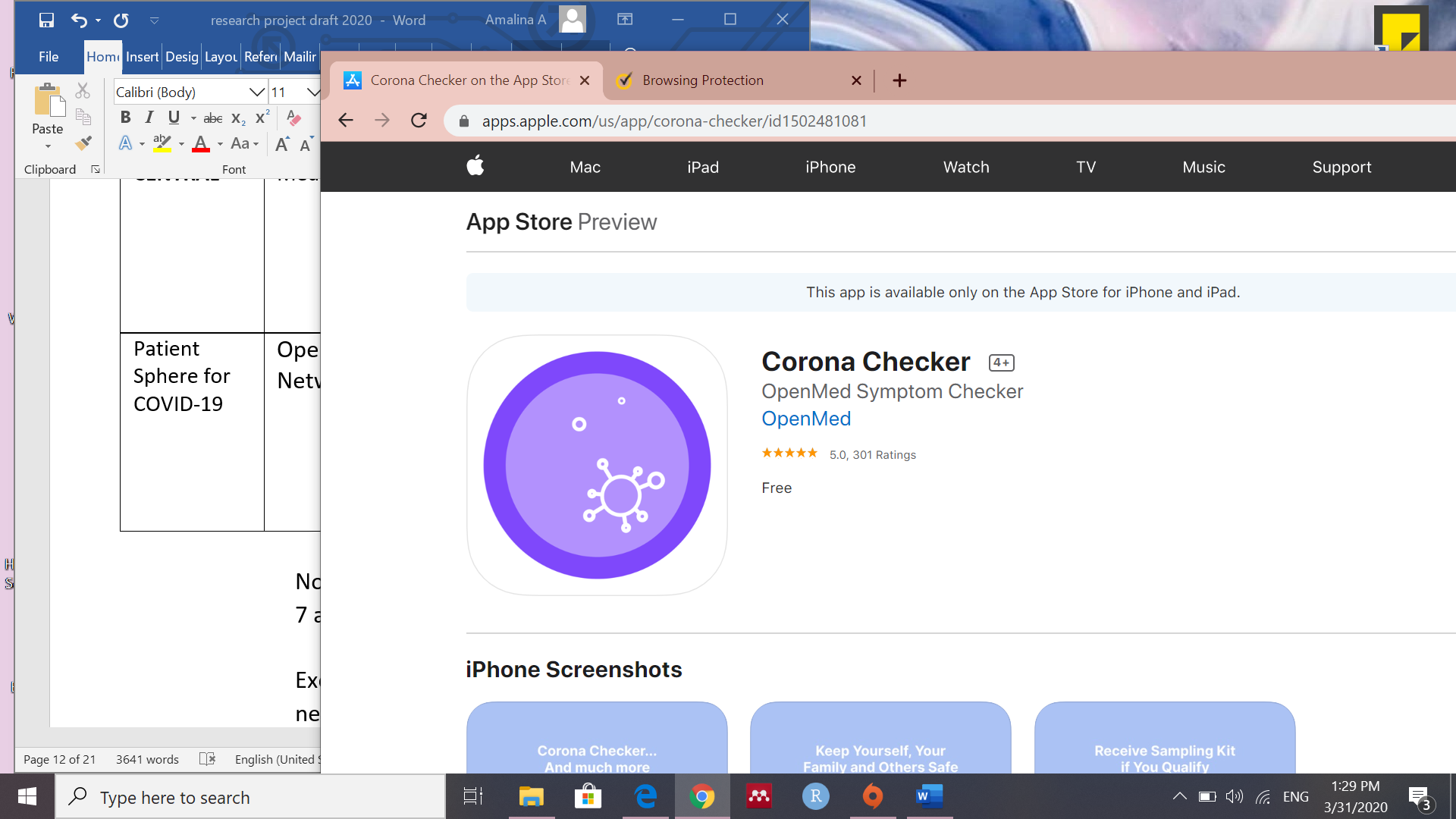 | | United States | | 9.9 MB | | 5.0  (n= 298) | 4+ | | Medical | |  | | N/A | |
| **5** | | **COVID-19!** | Nemocnice Milosrdnych bratri, p.o. | | 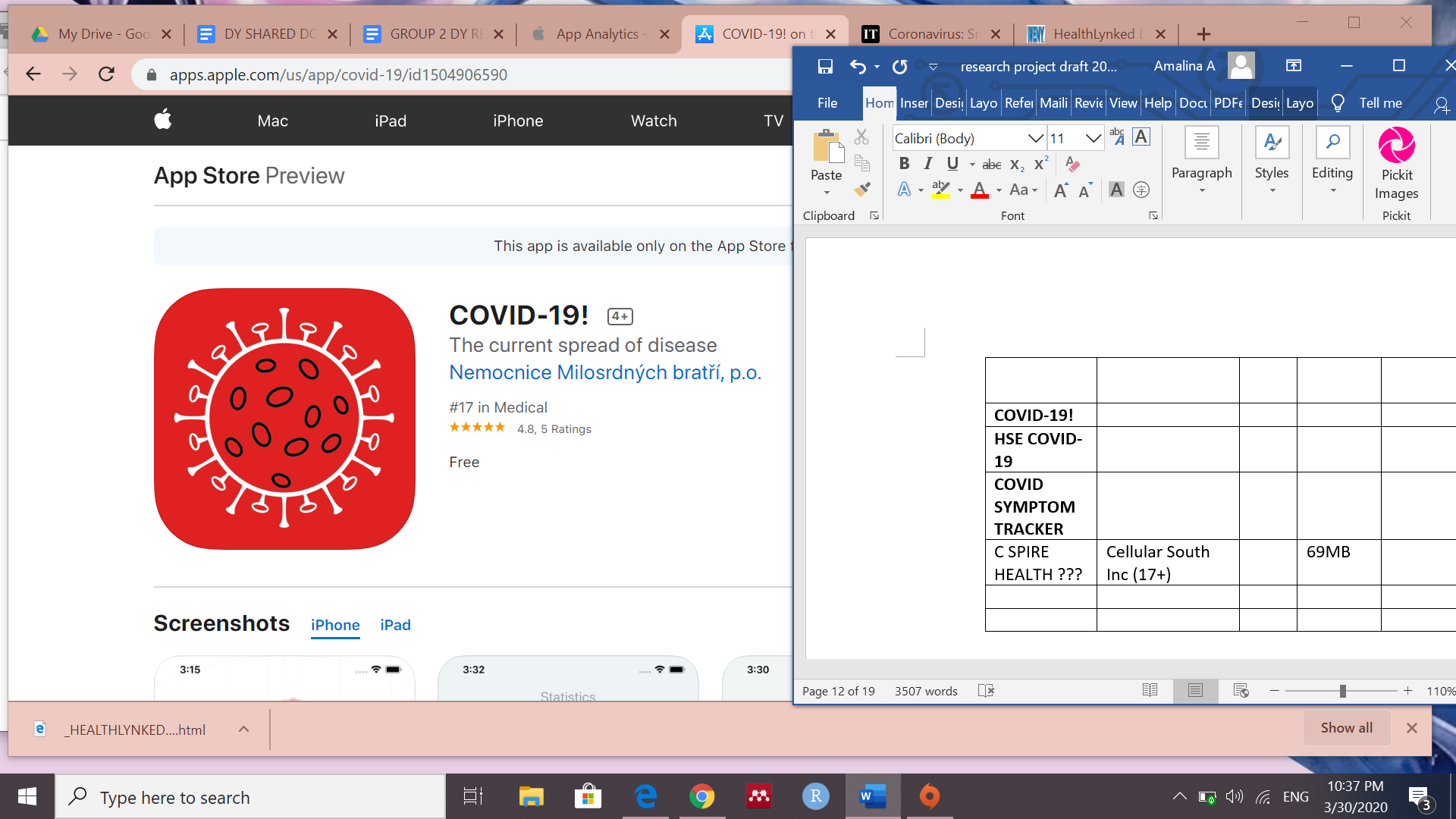 | | Czech Republic | | 103.3 MB | | 4.8  (n= 5) | 4+ | | #17 Medical #40, Health & Fitness | |  | | N/A | |
| **6** | | **COVID Symptom Tracker** | King’s College London and Zoe Global Limited | | 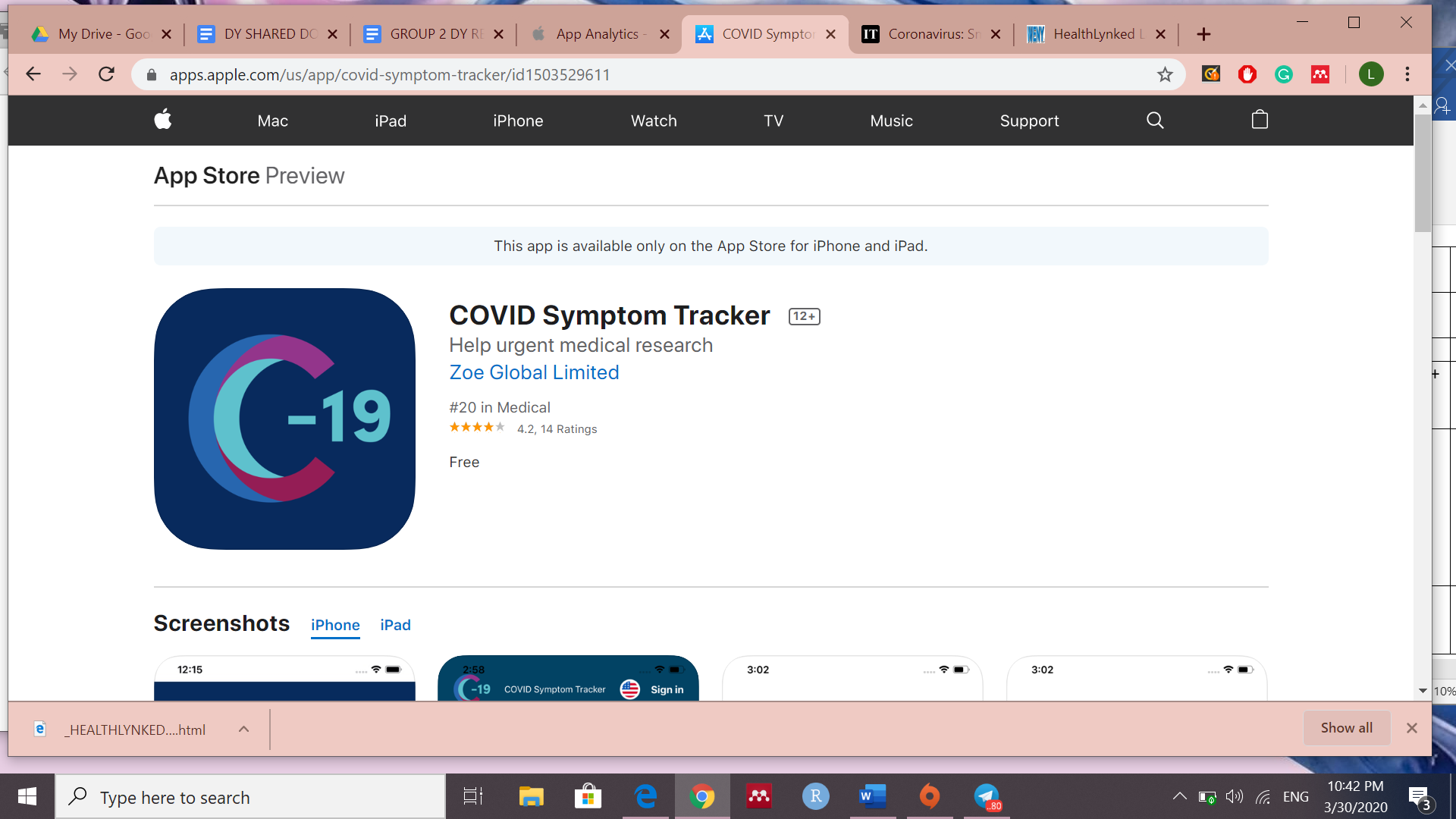 | | United Kingdom | | 88.0 MB | | 4.2  (n= 14) | 12+ | | #20 Medical | |  | | N/A | |
| **7** | | **HEALTHLYNKED COVID-19 TRACKER** | HealthLynked Corp. | | 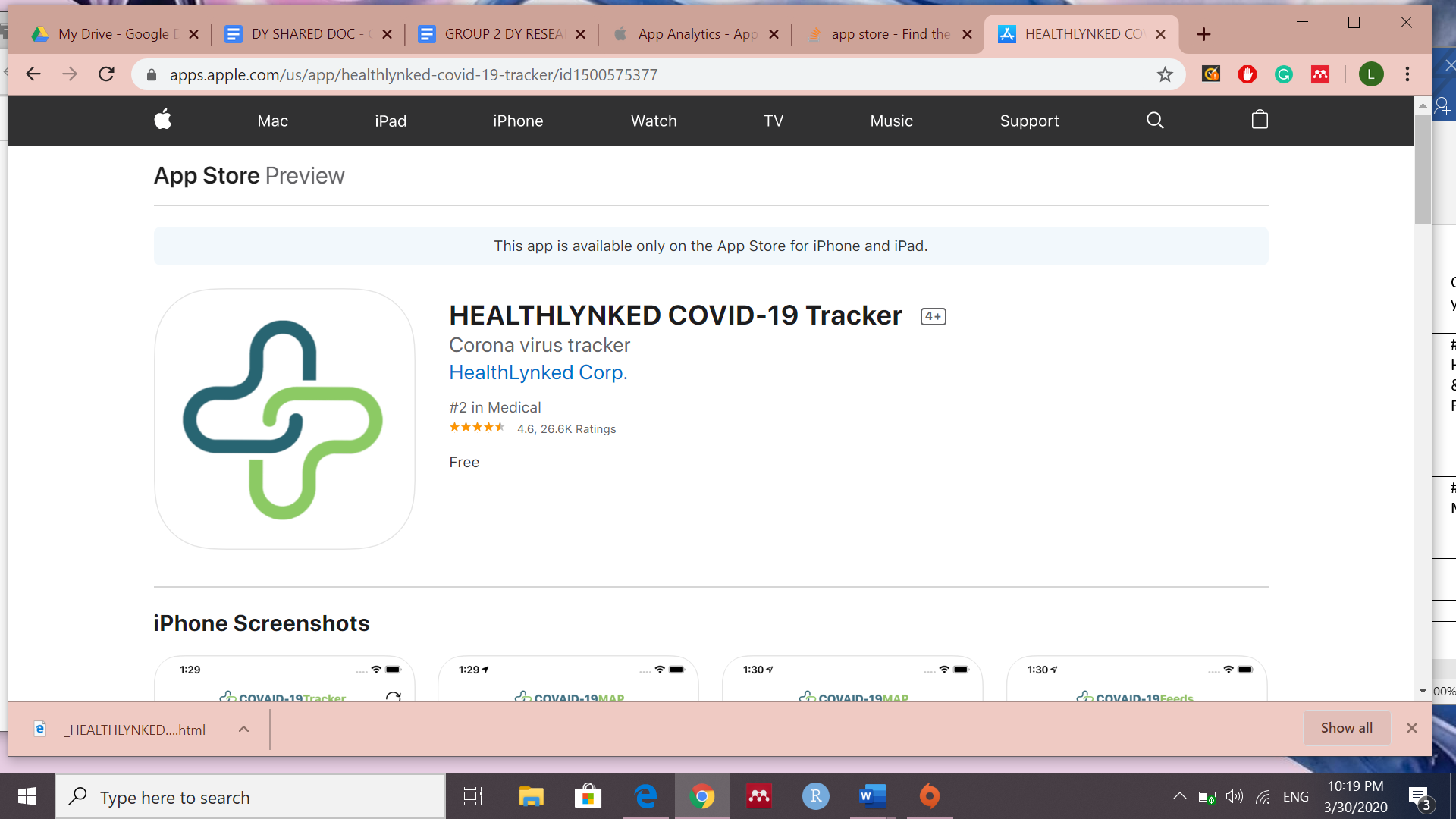 | | United States | | 34.6 MB | | 4.6  (n= 26.6K) | 4+ | | #2 Medical | |  | | N/A | |
| **8** | | **RELIEF CENTRAL** | Unbound Medicine | | 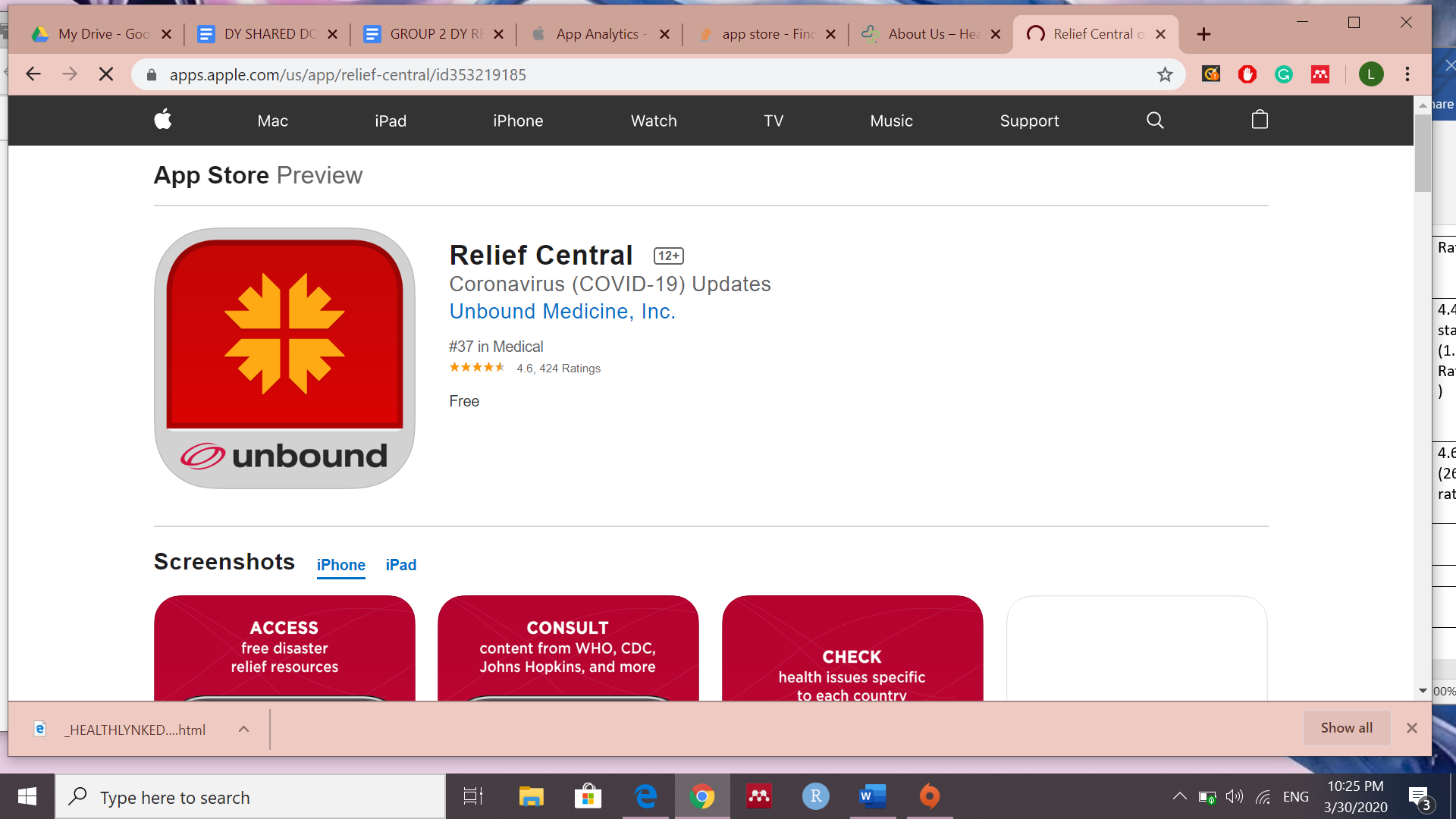 | | United States | | 16.3 MB | | 4.6  (n= 424) | 12+ | | #37 Medical | |  | | N/A | |
| **9** | | **Patient Sphere for COVID-19** | Open Cancer Network | | 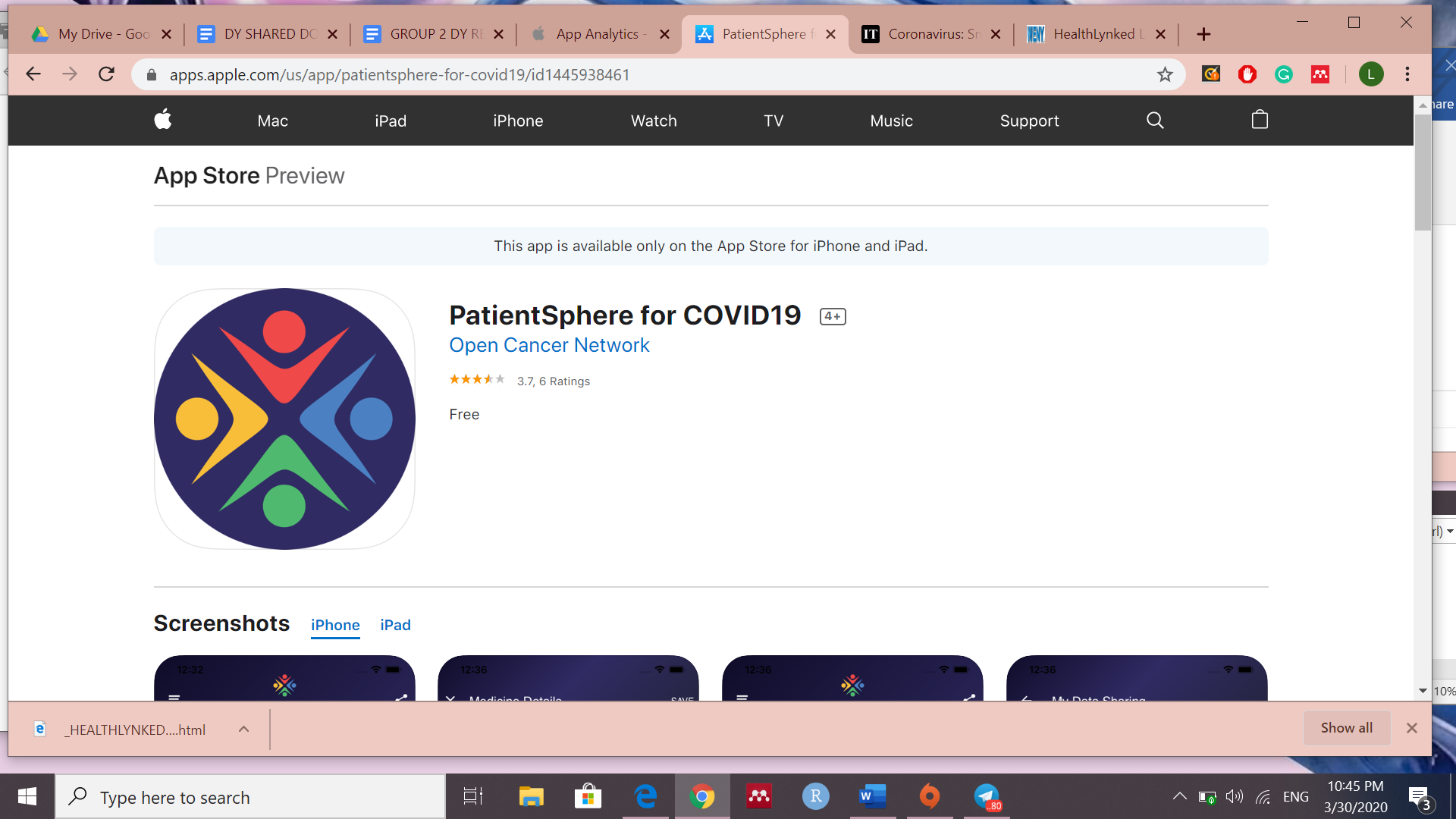 | | United States | | 22.8 MB | | 3.7  (n= 6) | 4+ | | Health & Fitness | |  | | N/A | |
| **10** | | **PreMedicus® ER** | PreMedicus LLC | | 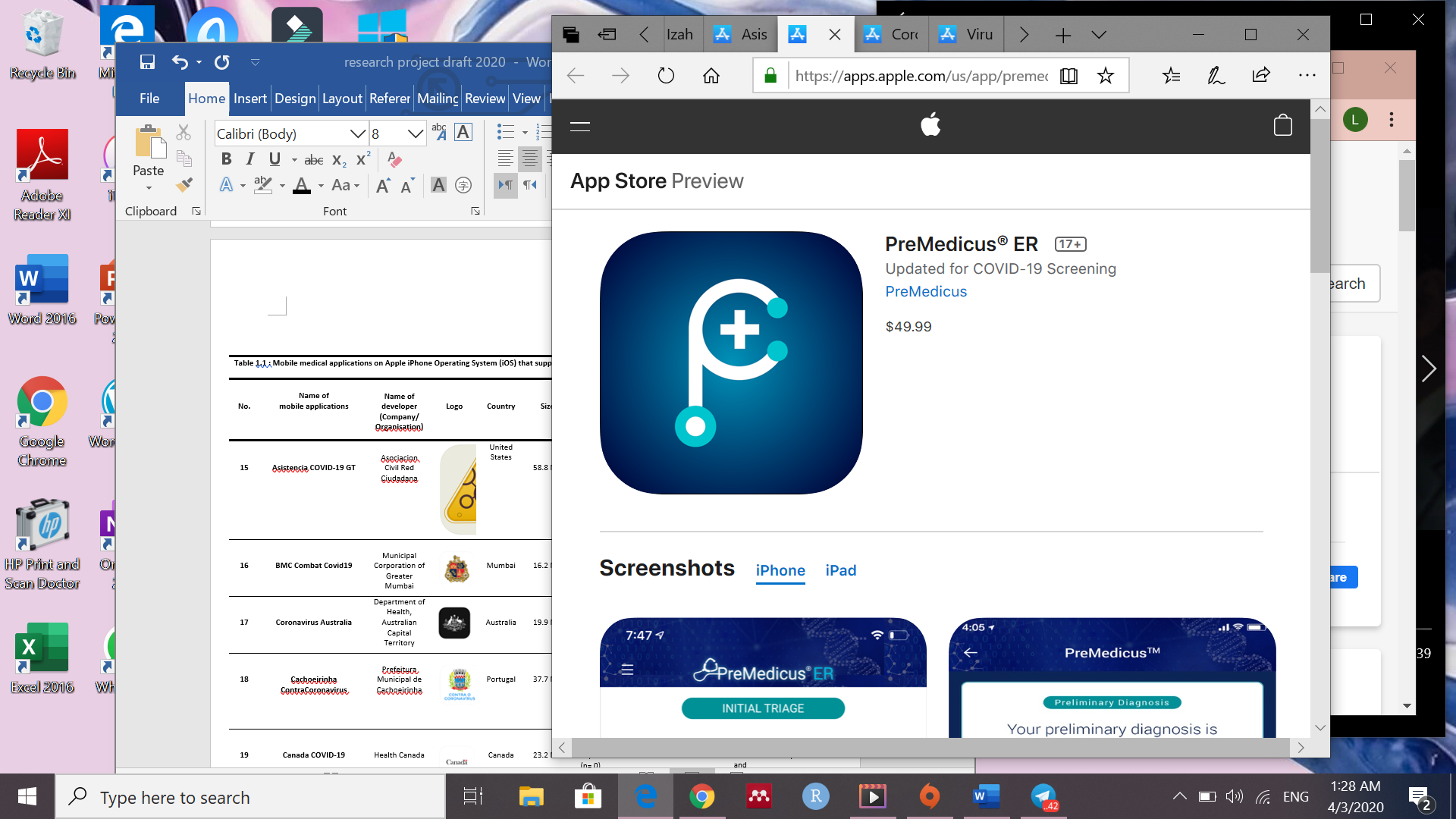 | | United States | | 62 MB | | N/A  (n= 0) | 17+ | | Medical | |  | | N/A | |
| **11** | | **Mobile Angel Cancer Telemed** | Mobile Angel Inc | | 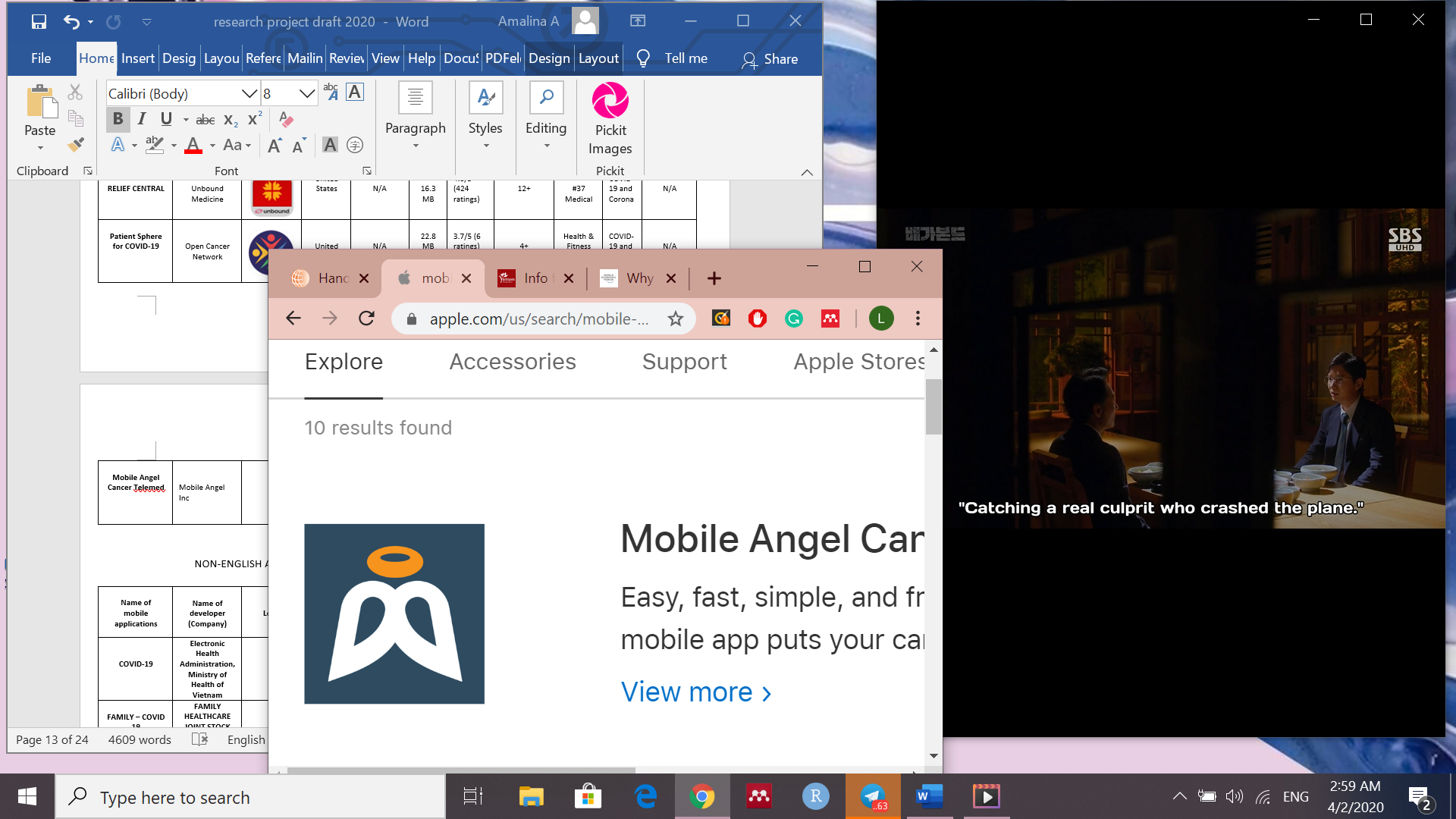 | | United States | | 140.4MB | | 5.0  (n= 12) | 12+ | | Medical | |  | | N/A | |
| **Table 1b. Country-specific apps** | | | | | | | | | | | | | | | | | | |  |
| **No.** | **Name of**  **mobile**  **applications** | | | **Name of developer (Company/**  **Organisation)** | | **Logo** | | **Country** | | **Size** | **User rating** | **Classification** | **Category** | |  | | **No. of downloads** | |  |
| **12** | **BC COVID-19 Support** | | | Province of British Columbia | | 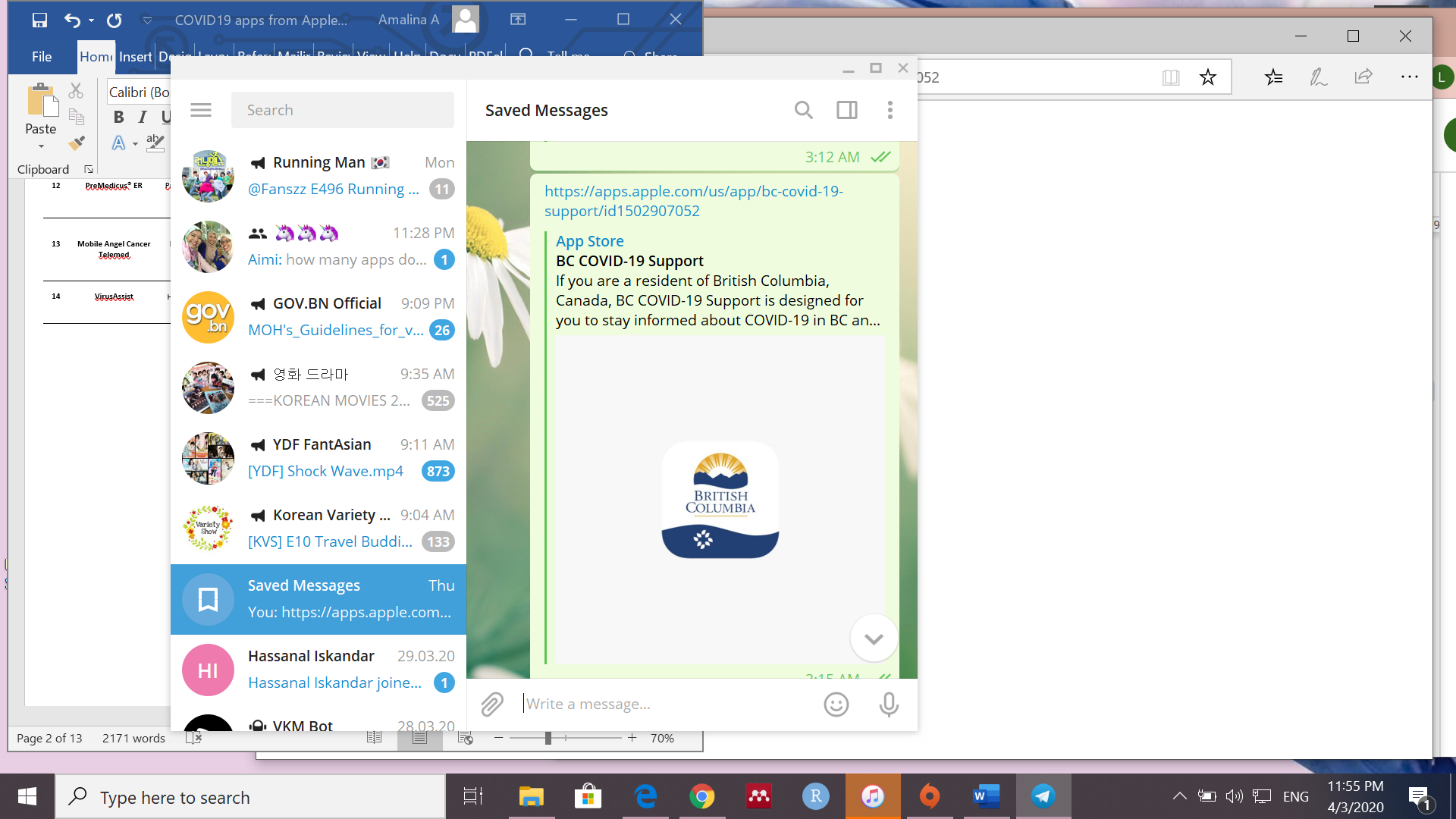 | | British Columbia | | 22.6 MB | 5.0  (n= 3) | 12+ | Medical | |  | | N/A | |  |
| **13** | **BMC Combat Covid19** | | | Municipal Corporation of Greater Mumbai | | 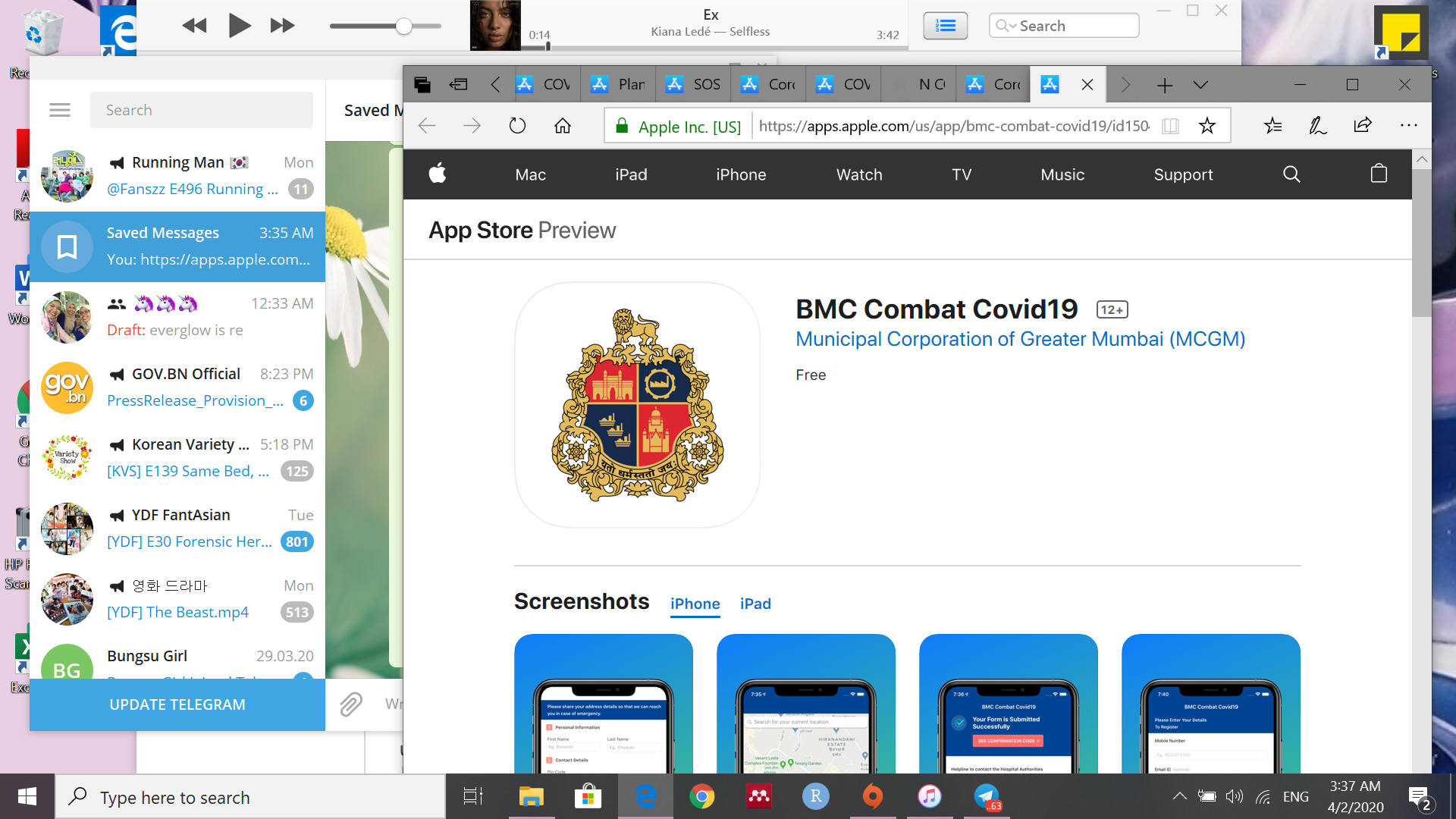 | | Mumbai | | 16.2 MB | N/A | 12+ | Health & Fitness | |  | | N/A | |  |
| **14** | **Canada COVID-19** | | | Health Canada | | 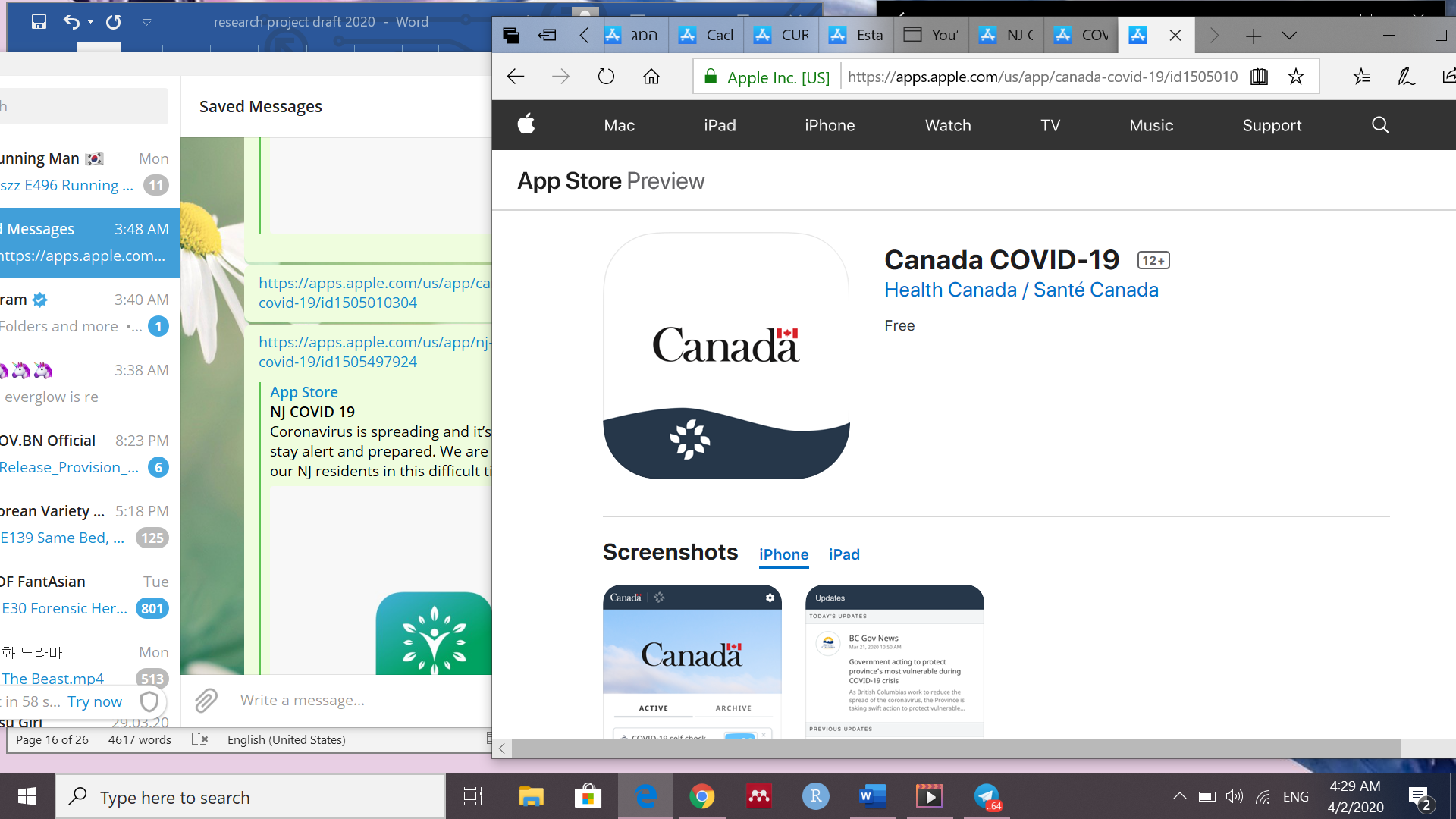 | | Canada | | 23.2 MB | N/A  (n= 0) | 12+ | Medical | |  | | N/A | |  |
| **15** | **Coronavirus Australia** | | | Department of Health, Australian Capital Territory | | 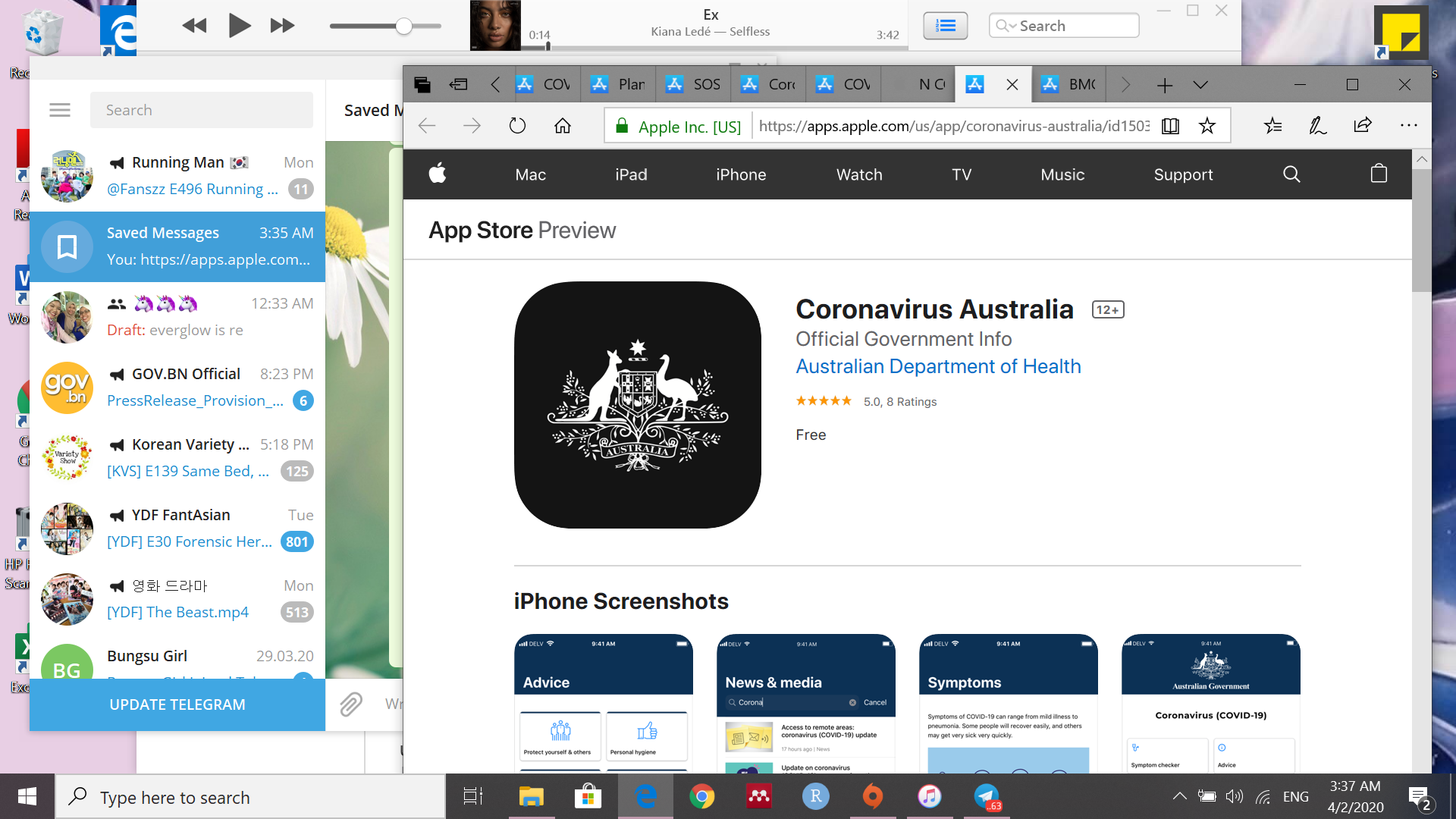 | | Australia | | 19.9 MB | 5.0  (n= 8) | 12+ | Medical | |  | | N/A | |  |
| **16** | **Corona-Care** | | | PaxeraHealth Corp | | 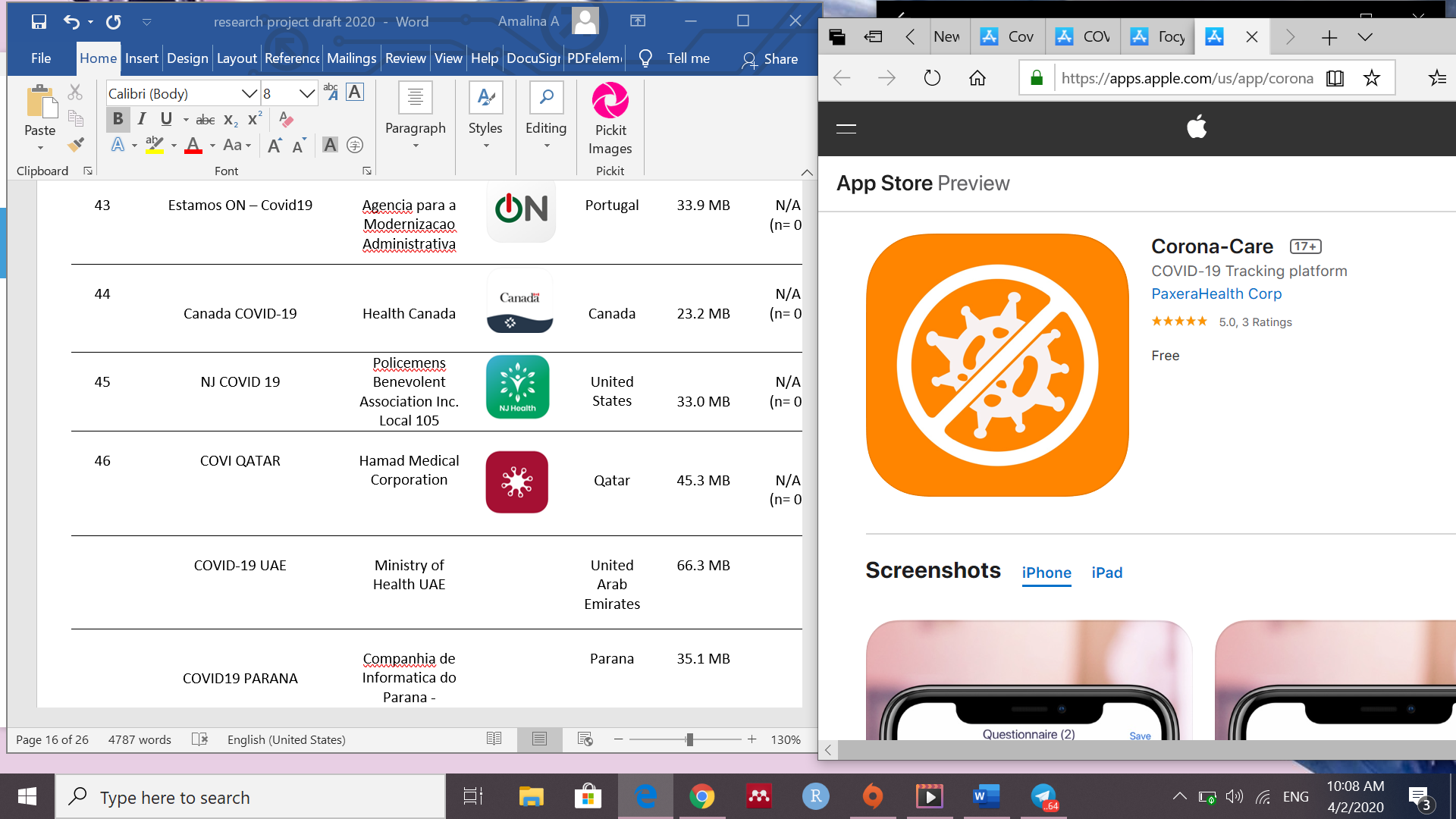 | | United States | | 160.9 MB | 5.0  (n= 3) | 17+ | Medical | |  | | N/A | |  |
| **17** | **COVA Punjab** | | | Government of Punjab | | 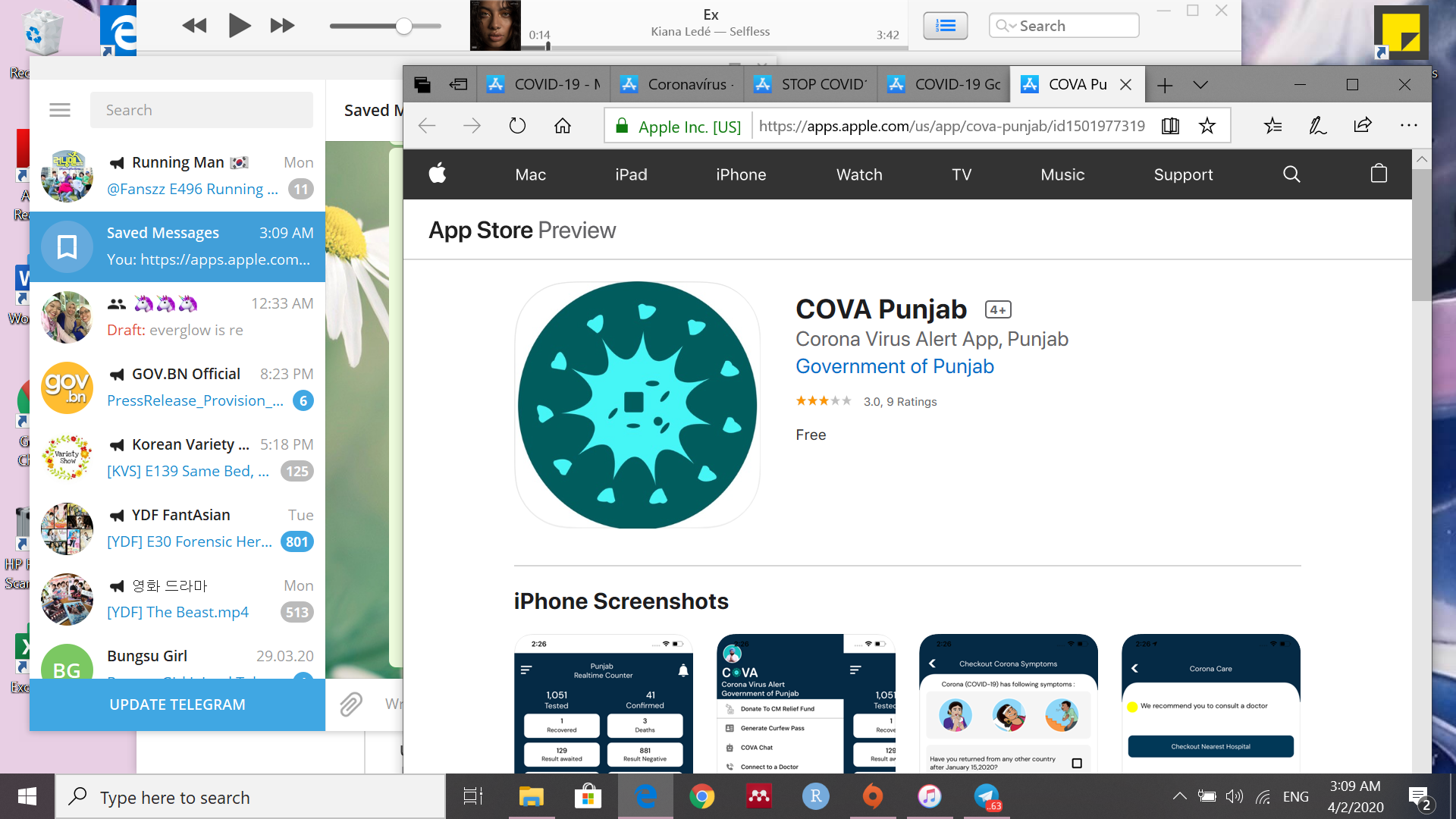 | | India | | 14.8 MB | 3.0  (n= 9) | 4+ | Health & Fitness | |  | | N/A | |  |
| **18** | **COVID-19 Gov PK** | | | National Information Technology Board | | 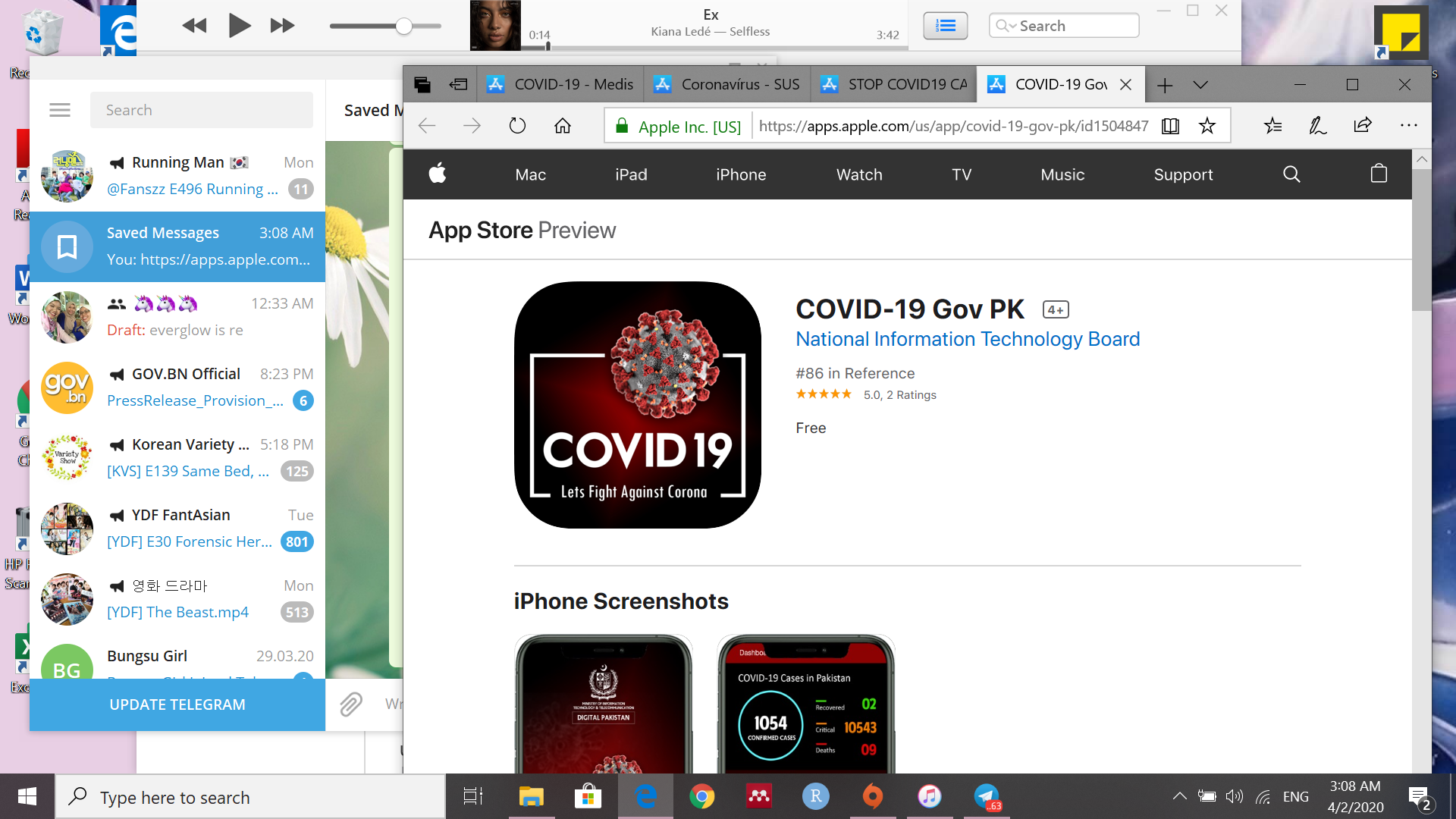 | | Pakistan | | 18.4 MB | 5.0  (n= 1) | 4+ | #131 Reference | |  | | N/A | |  |
| **19** | **Covidom Patient** | | | Assistance Publique-Hopitaux de Paris | | 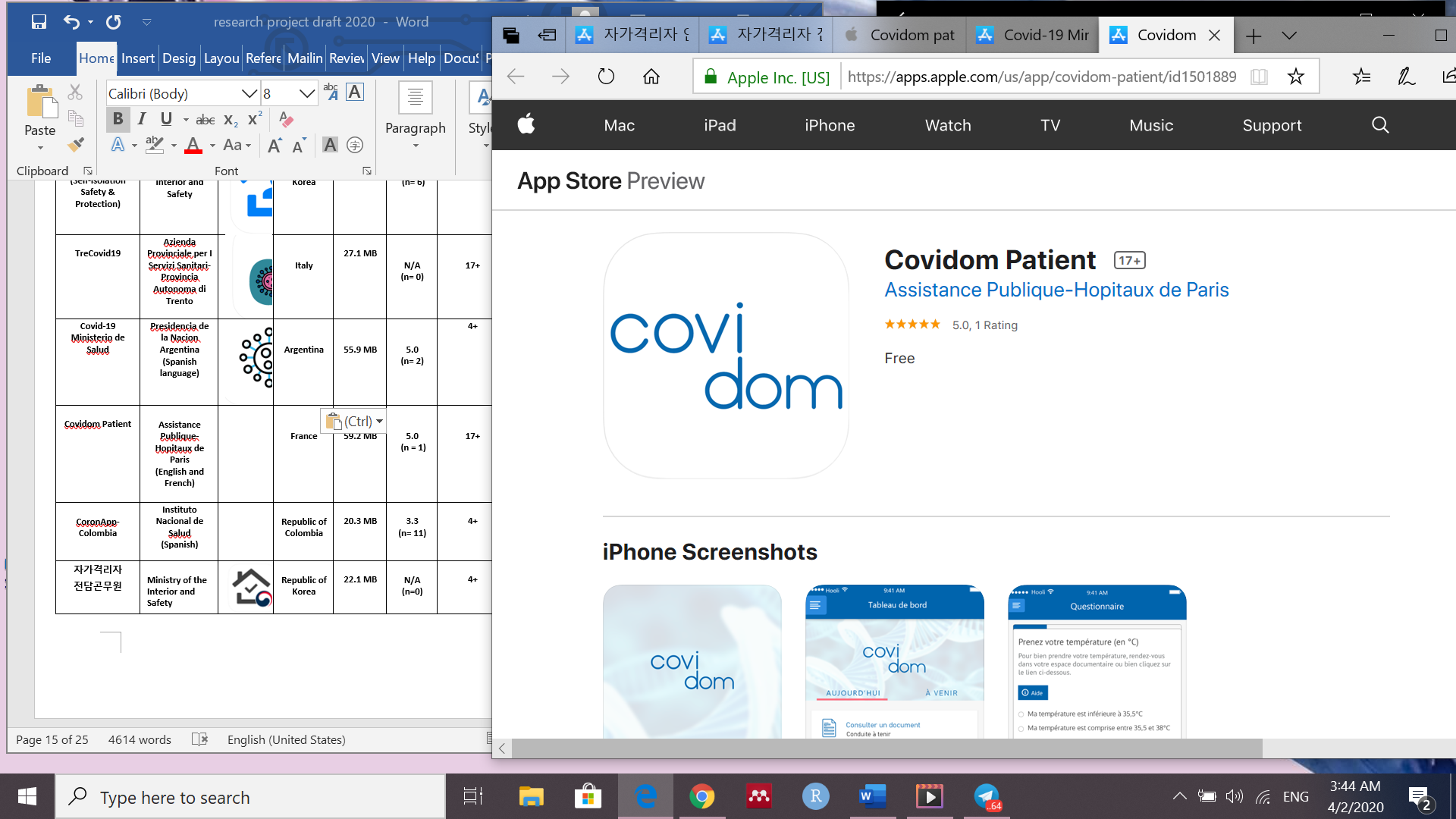 | | France | | 59.2 MB | 5.0  (n = 1) | 4+ | Medical | |  | | N/A | |  |
| **20** | **COVI QATAR** | | | Hamad Medical  Corporation | | 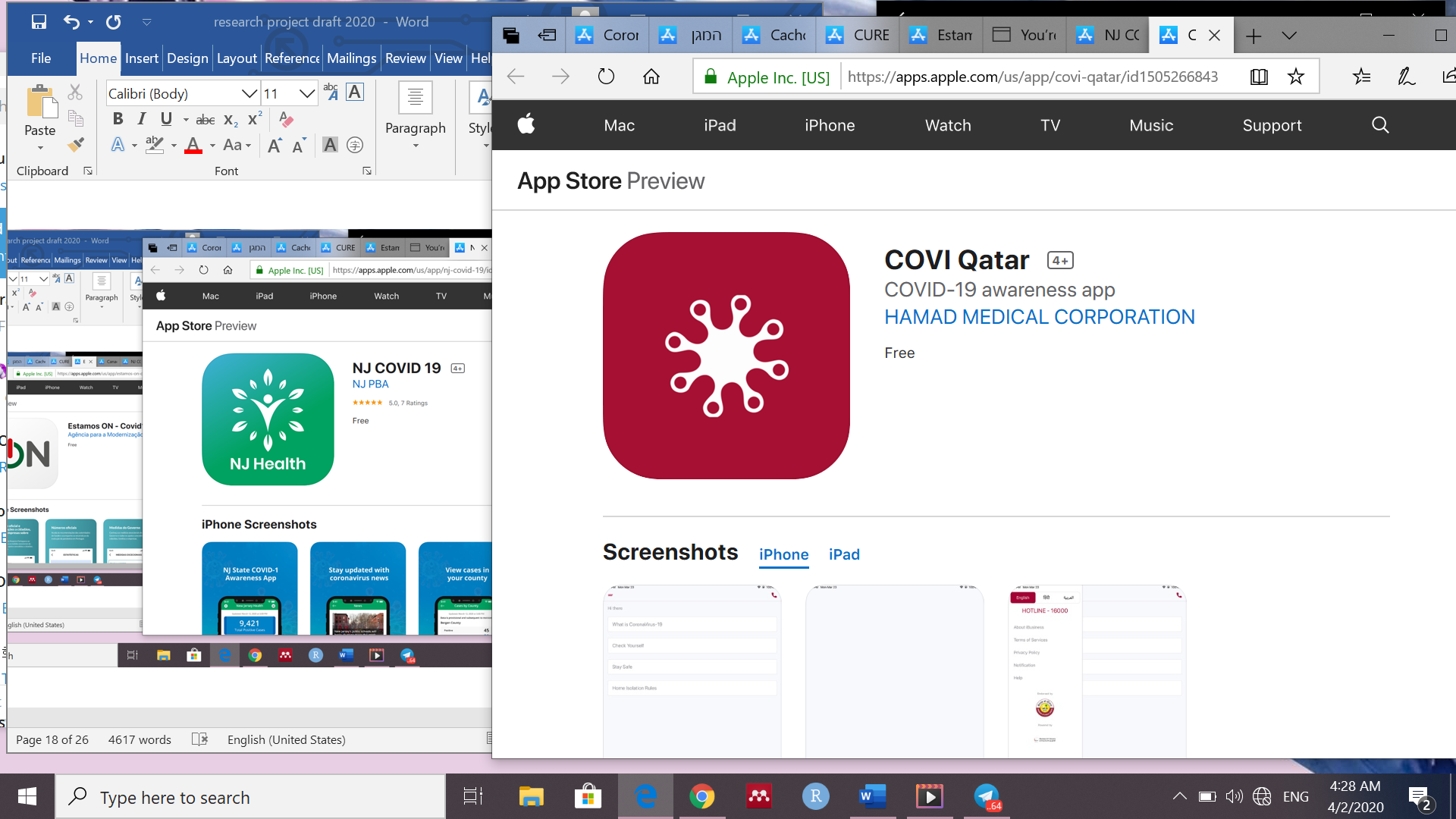 | | Qatar | | 45.3 MB | N/A  (n= 0) | 4+ | Health & Fitness | |  | | N/A | |  |
| **21** | **COVID-19 UAE** | | | Ministry of Health UAE | | 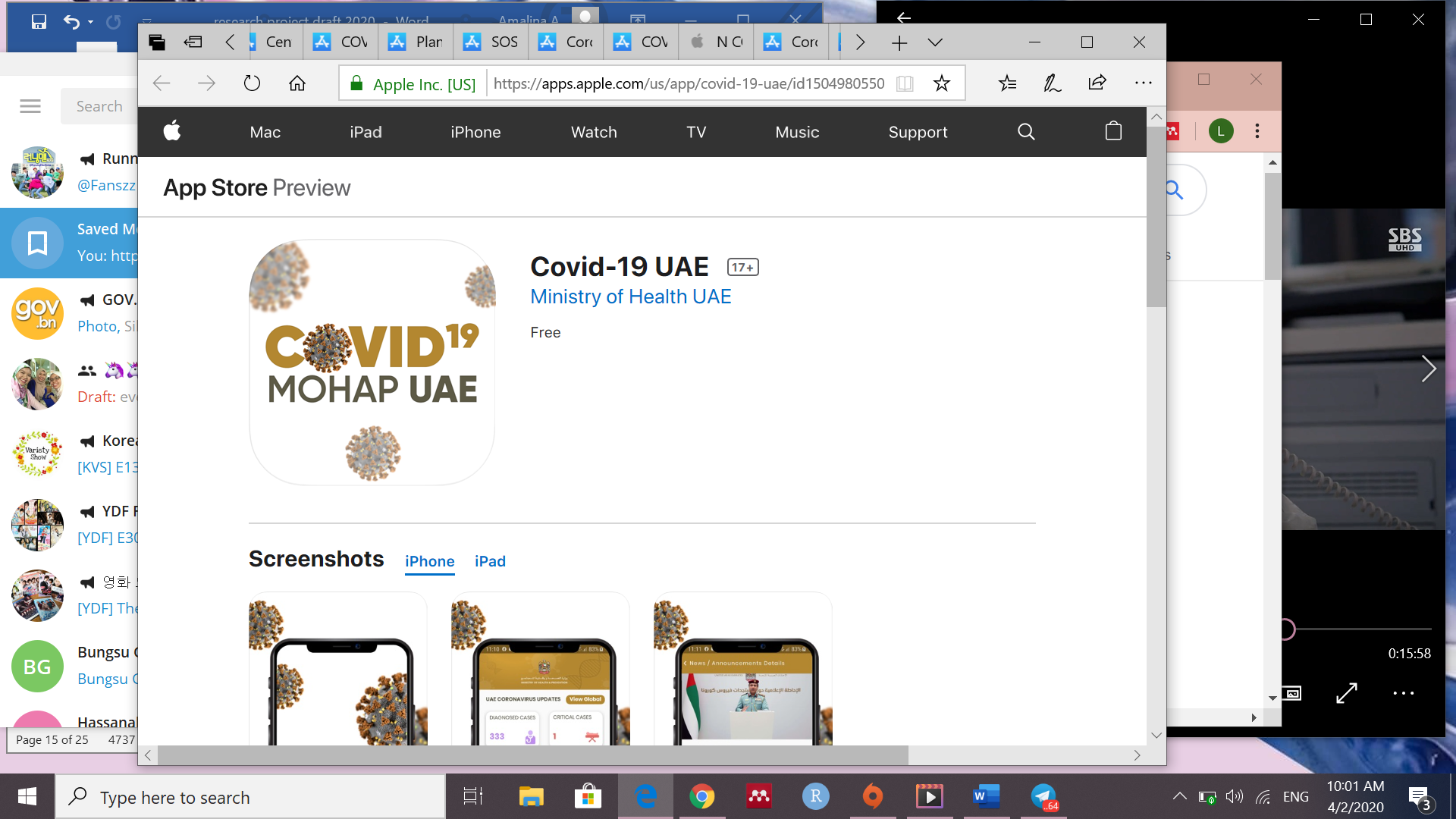 | | United Arab Emirates | | 66.3 MB | N/A  (n= 0) | 17+ | Health & Fitness | |  | | N/A | |  |
| **22** | **CUREiTT** | | | Cureitt Inc | | 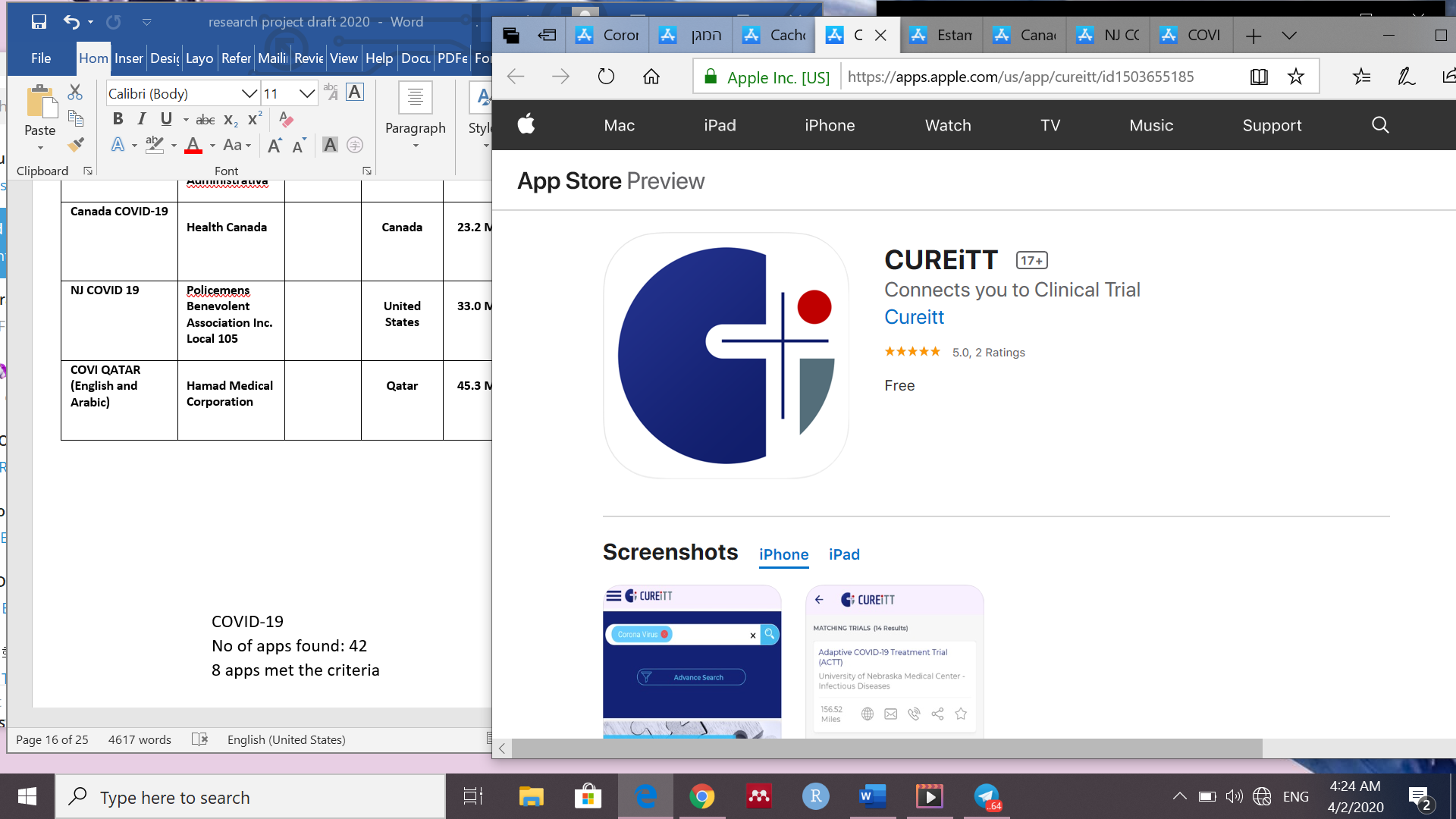 | | United States | | 103.5 MB | 5.0  (n= 2) | 17+ | Medical | |  | | N/A | |  |
| **23** | **HSE COVID-19** | | | patientMpower Ltd | | 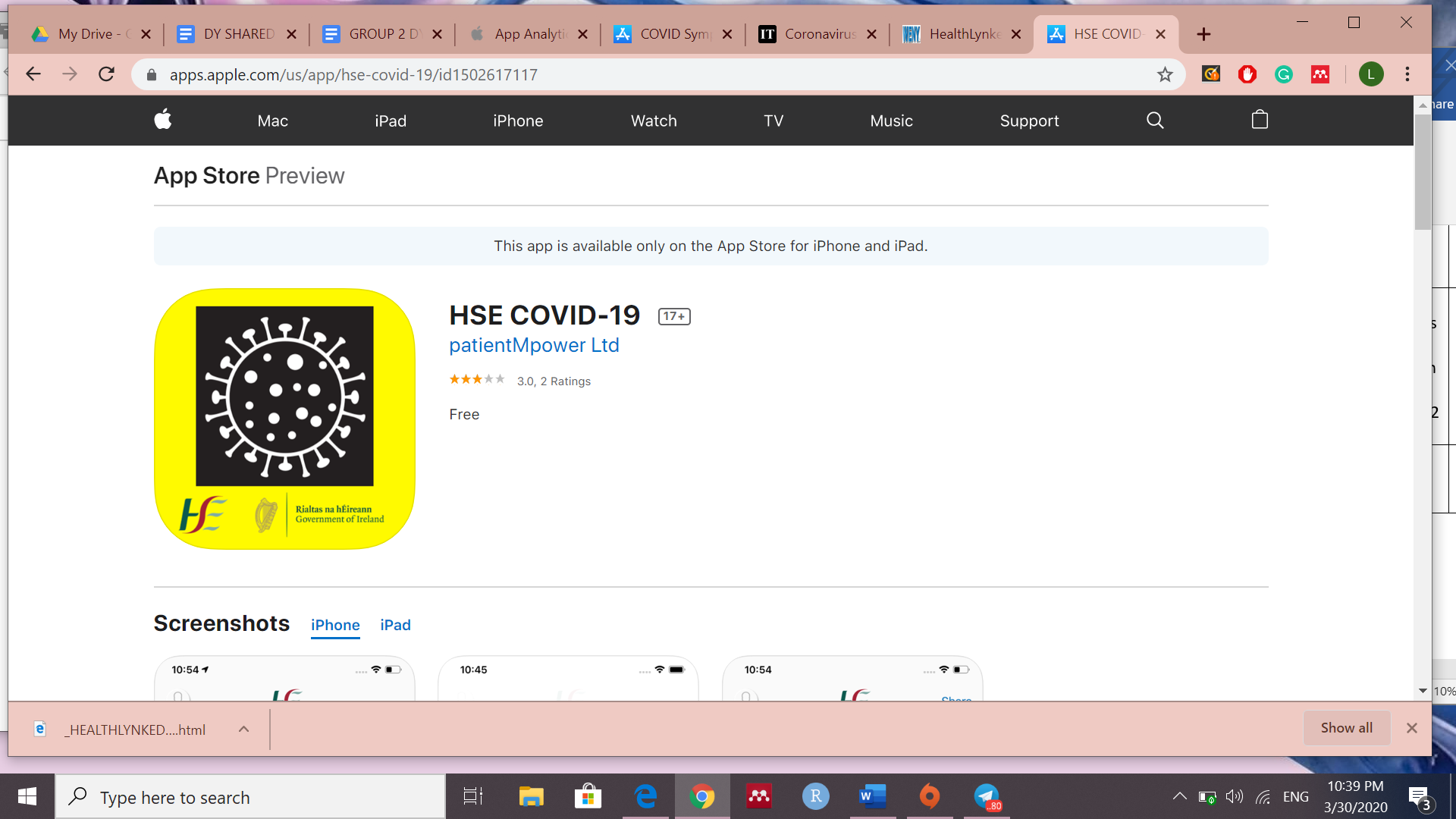 | | Ireland | | 163.9 MB | 3.0  (n= 2) | 17+ | Medical | |  | | N/A | |  |
| **24** | **NCOVI** | | | Authority of Information Technology Application | | 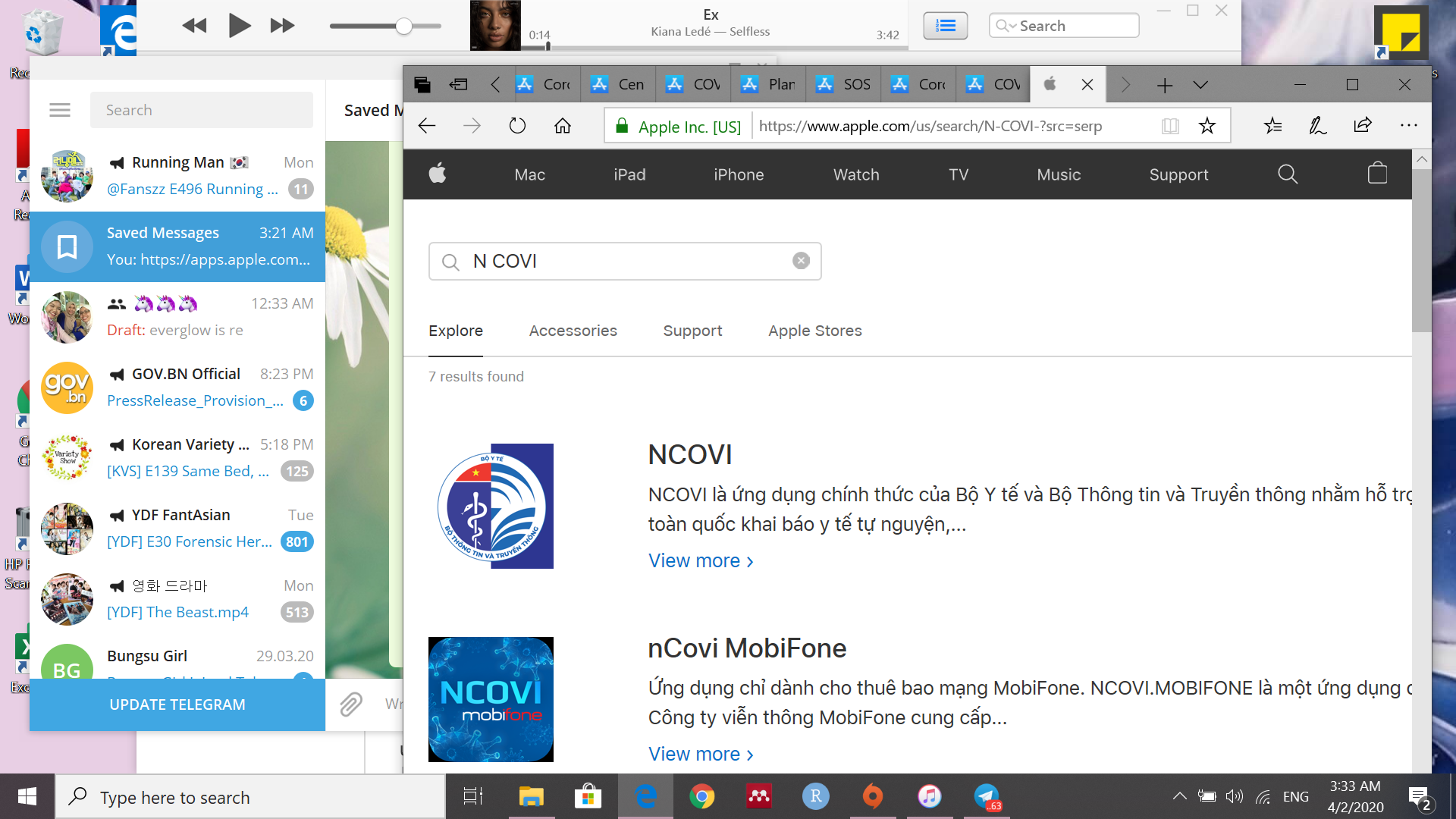 | | Vietnam | | 25.5 MB | 4.4  (n= 56) | 4+ | Health & Fitness | |  | | N/A | |  |
| **25** | **NJ COVID 19** | | | Policemens Benevolent Association Inc. Local 105 | | 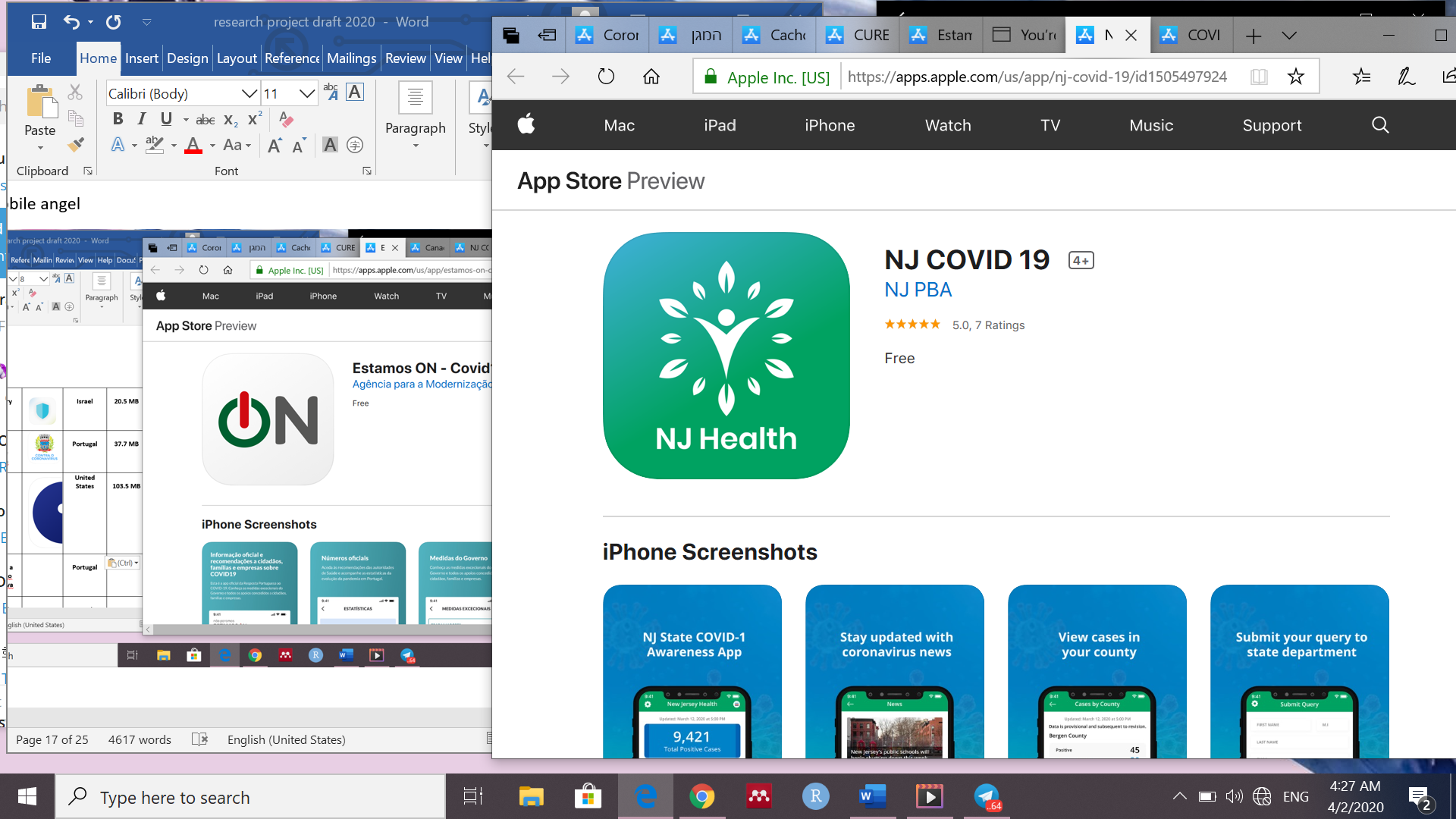 | | United States | | 33.0 MB | N/A  (n= 0) | 4+ | News | |  | | N/A | |  |
| **26** | **STOP COVID19 CAT** | | | Generalitat de  Catalunya | | 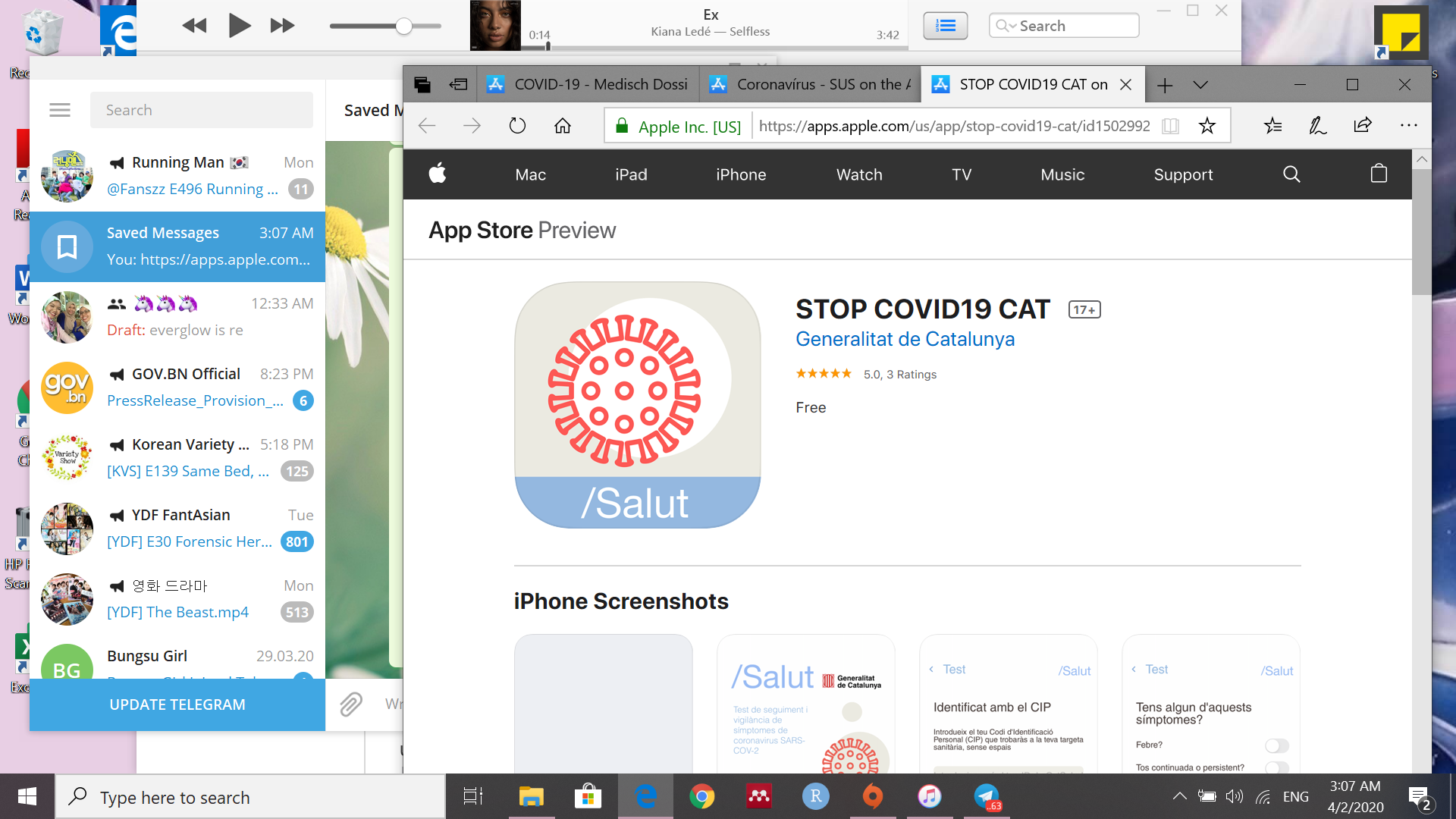 | | Spain | | 17.3 MB | 5.0  (n= 3) | 17+ | Medical | |  | | N/A | |  |
| **27** | **Tarassud** | | | Ministry of Health, Sultanate of Oman | | 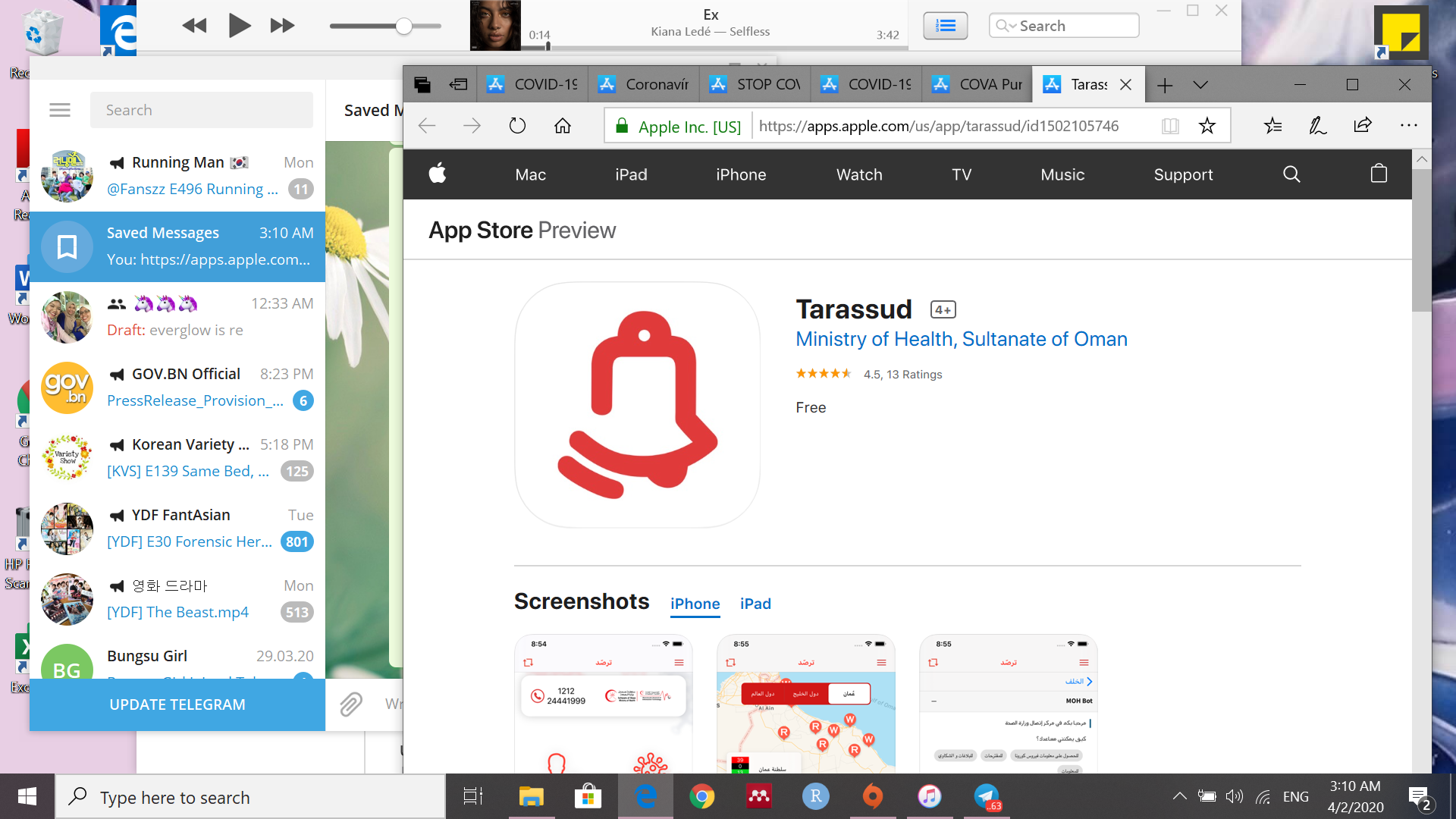 | | Oman | | 42.4 MB | 4.5  (n= 12) | 4+ | Medical | |  | | N/A | |  |
| **28** | **TraceTogether** | | | Government Technology Agency | | 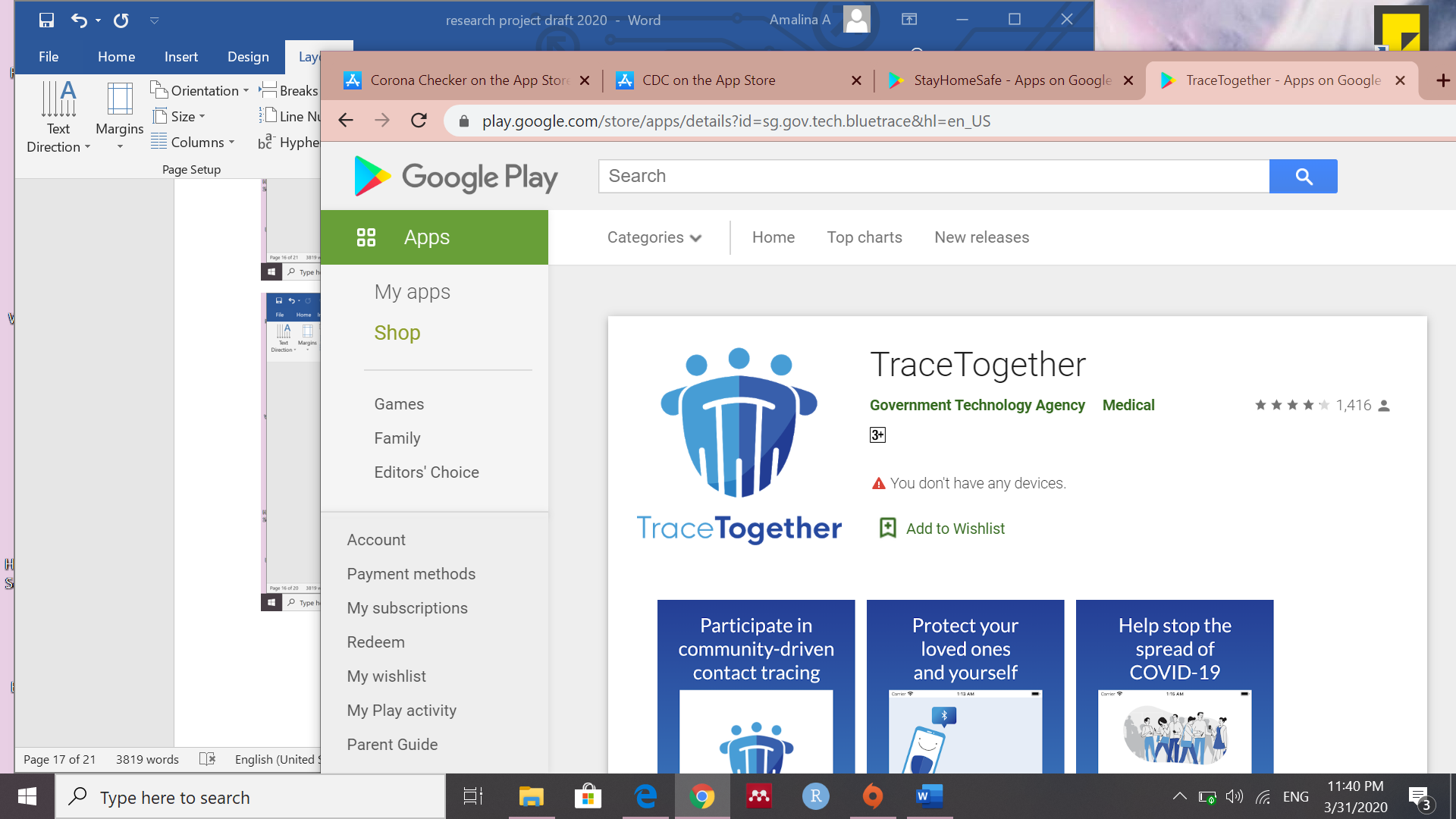 | | Singapore | | 39.4 MB | 5.0  (n= 4) | 12+ | Medical | |  | | N/A | |  |
| **29** | **자가격리자 안전보호**  **(Self-Isolator Safety & Protection)** | | | Ministry of the Interior and Safety | | 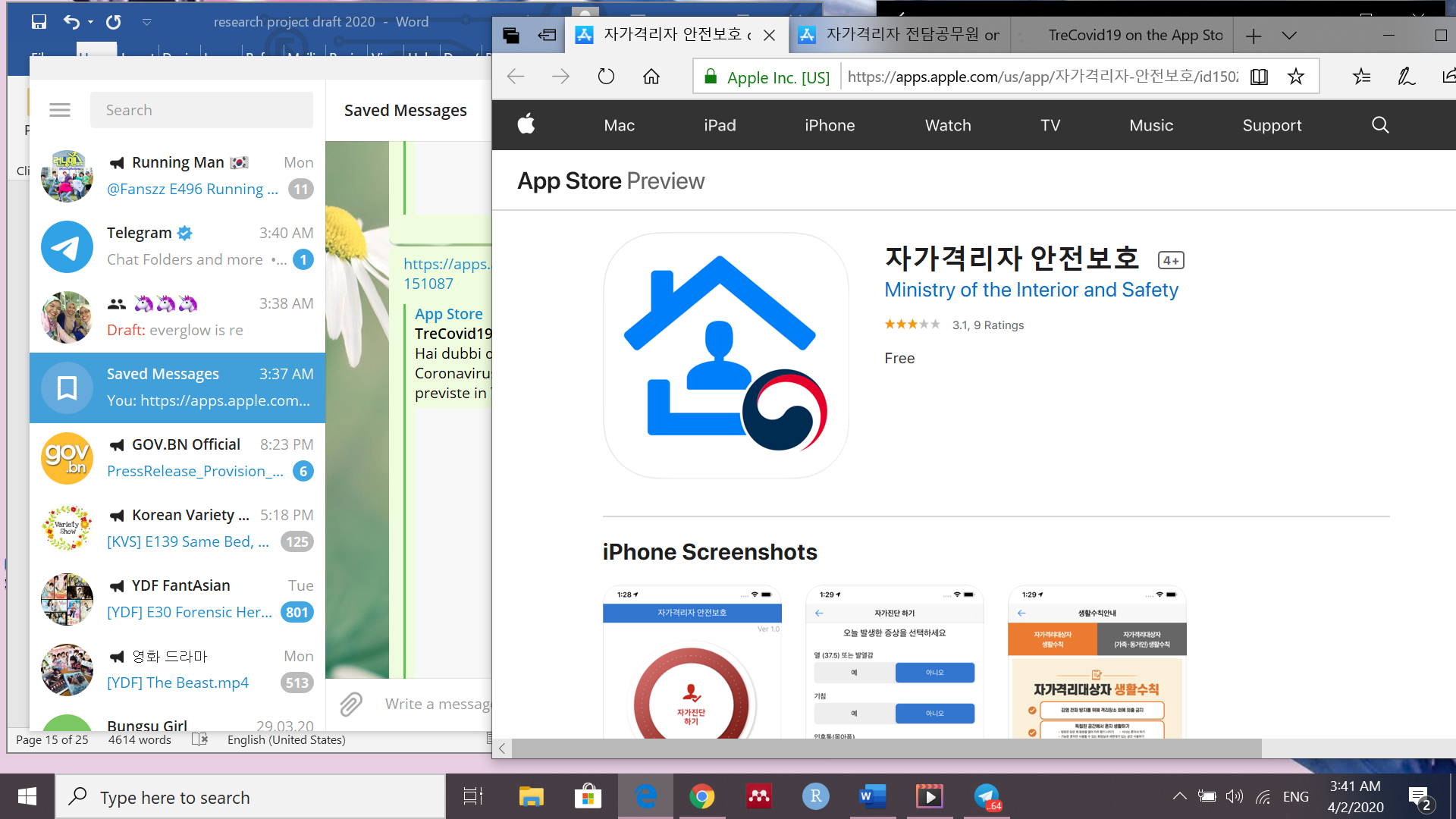 | | Republic of Korea | | 25.4 MB | 4.0  (n= 6) | 4+ | Health & Fitness | |  | | N/A | |  |
| **30** | **자가격리자 전담곤무원**  **(Self-isolating Government Officials)** | | | Ministry of the Interior and Safety | | 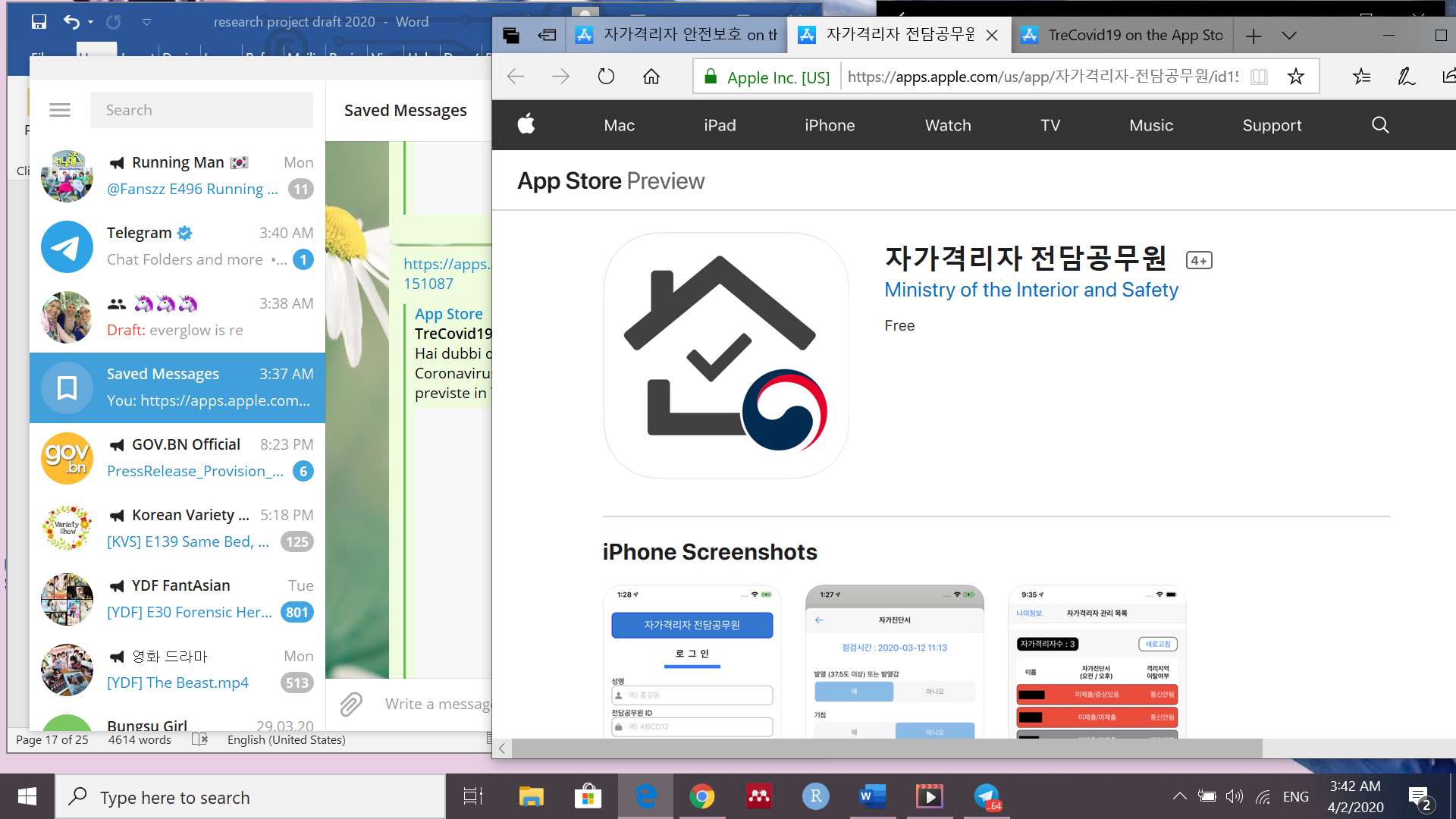 | | Republic of Korea | | 22.1 MB | N/A  (n= 0) | 4+ | Lifestyle | |  | | N/A | |  |

| **Table 1c. Non-English language apps** | | | | | | | | | | |
| --- | --- | --- | --- | --- | --- | --- | --- | --- | --- | --- |
| **No.** | **Name of**  **mobile**  **applications** | **Name of developer (Company/**  **Organisation)** | **Logo** | **Country** | **Size** | **User rating** | **Classification** | **Category** |  | **No. of downloads** |
| **31** | **Asistencia COVID-19 GT** | Asociacion Civil Red Ciudadana | 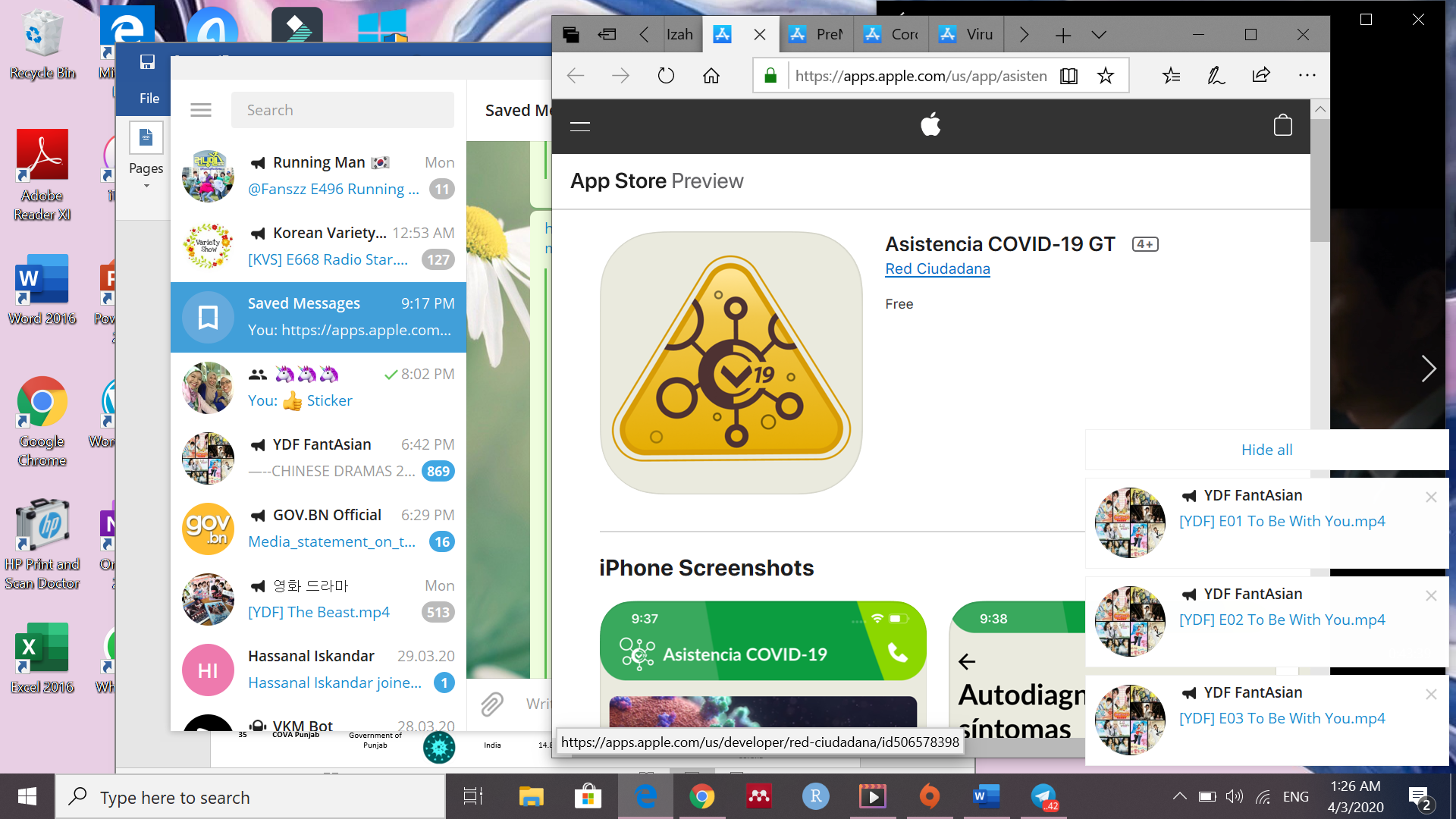 | United States | 58.8 MB | N/A  (n= 0) | 4+ | Reference |  | N/A |
| **32** | **Cachoeirinha ContraCoronavirus** | Prefeitura Municipal de Cachoeirinha | 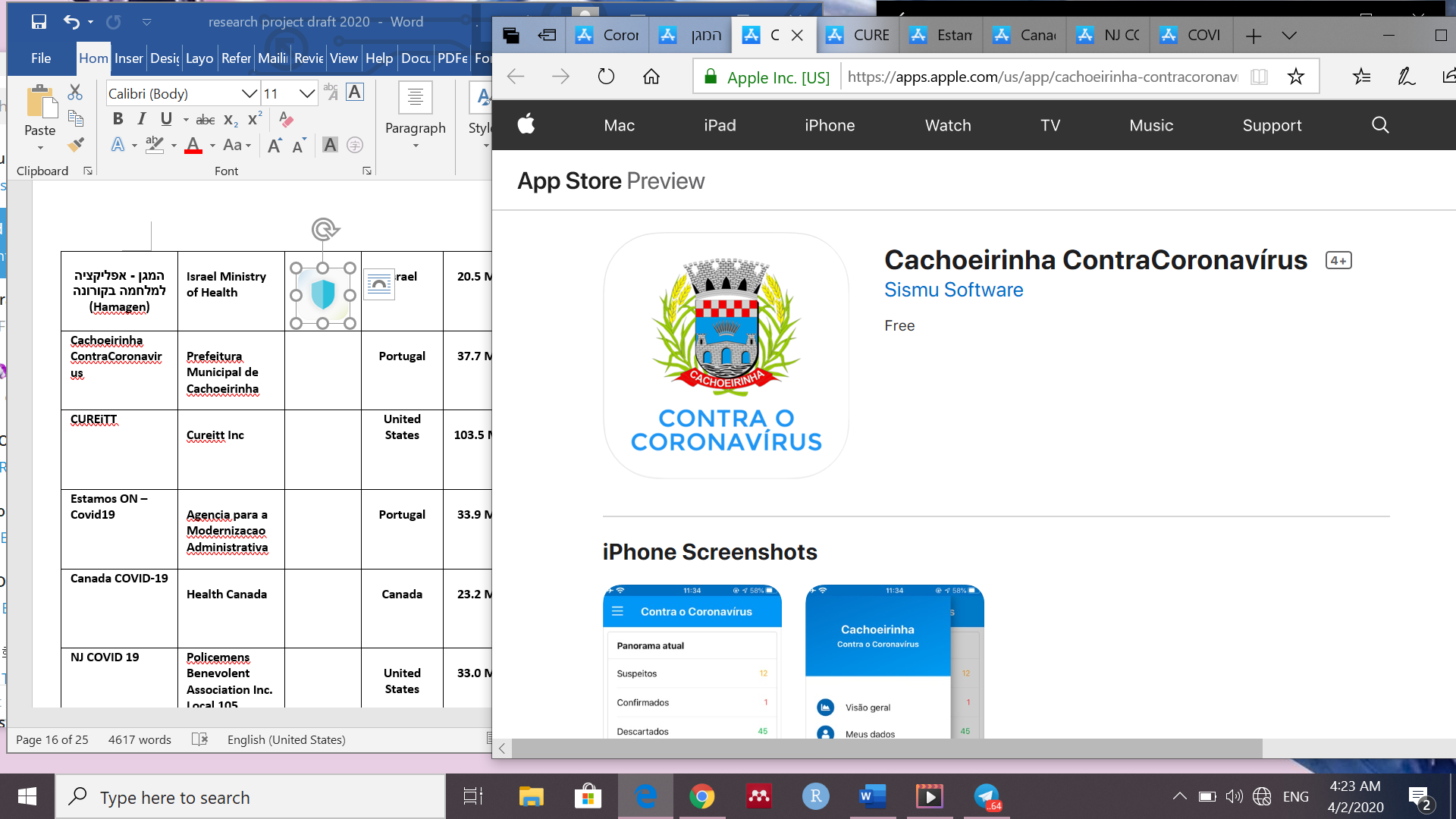 | Portugal | 37.7 MB | N/A  (n= 0) | 4+ | Health & Fitness |  | N/A |
| **33** | **Central Coast COVID-19**  **Info** | Radiology Associates | 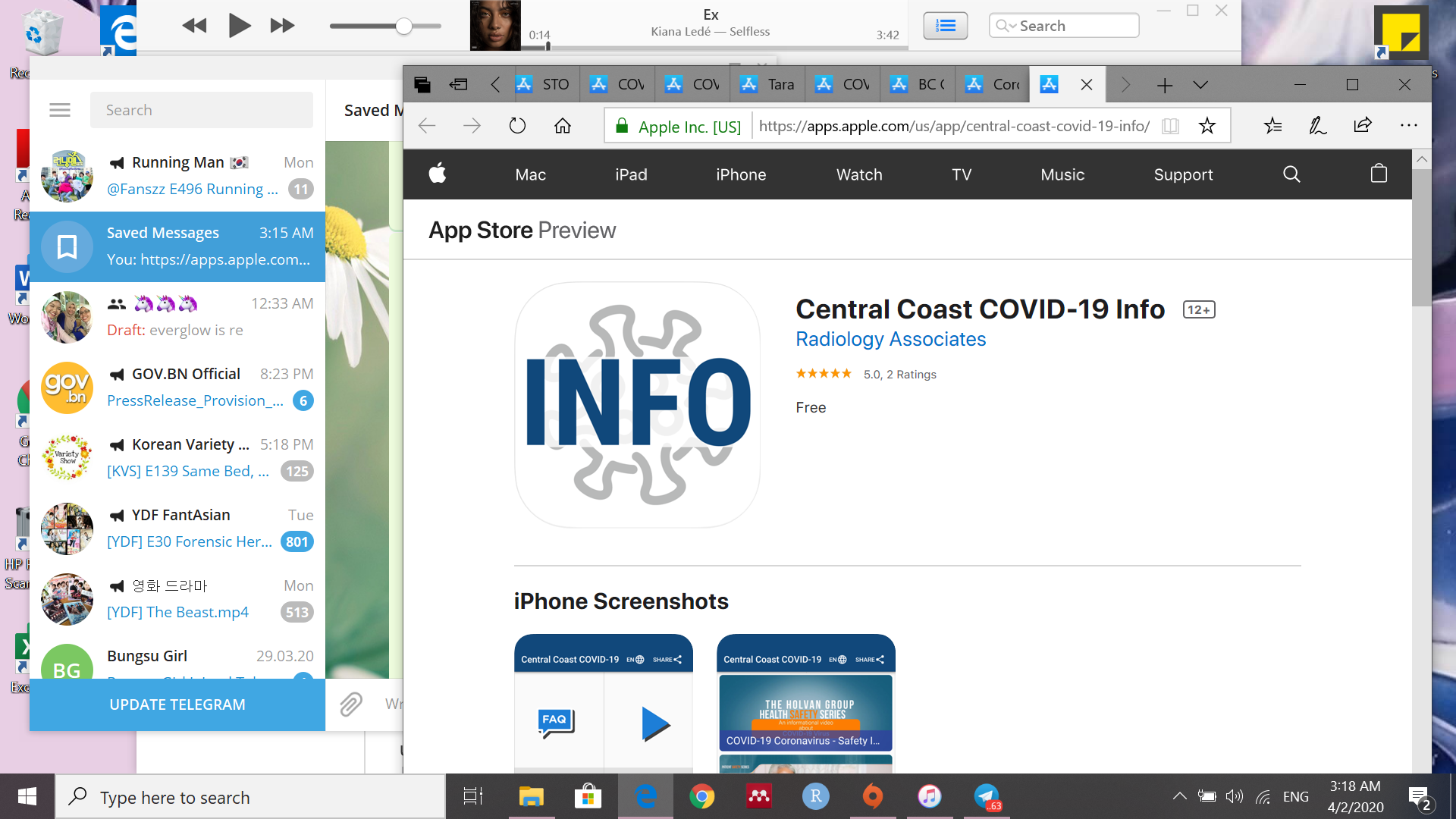 | Central Coast Area | 2.8 MB | 5.0  (n= 2) | 12+ | Health & Fitness |  | N/A |
| **34** | **CoronApp-Colombia** | Instituto Nacional de Salud | 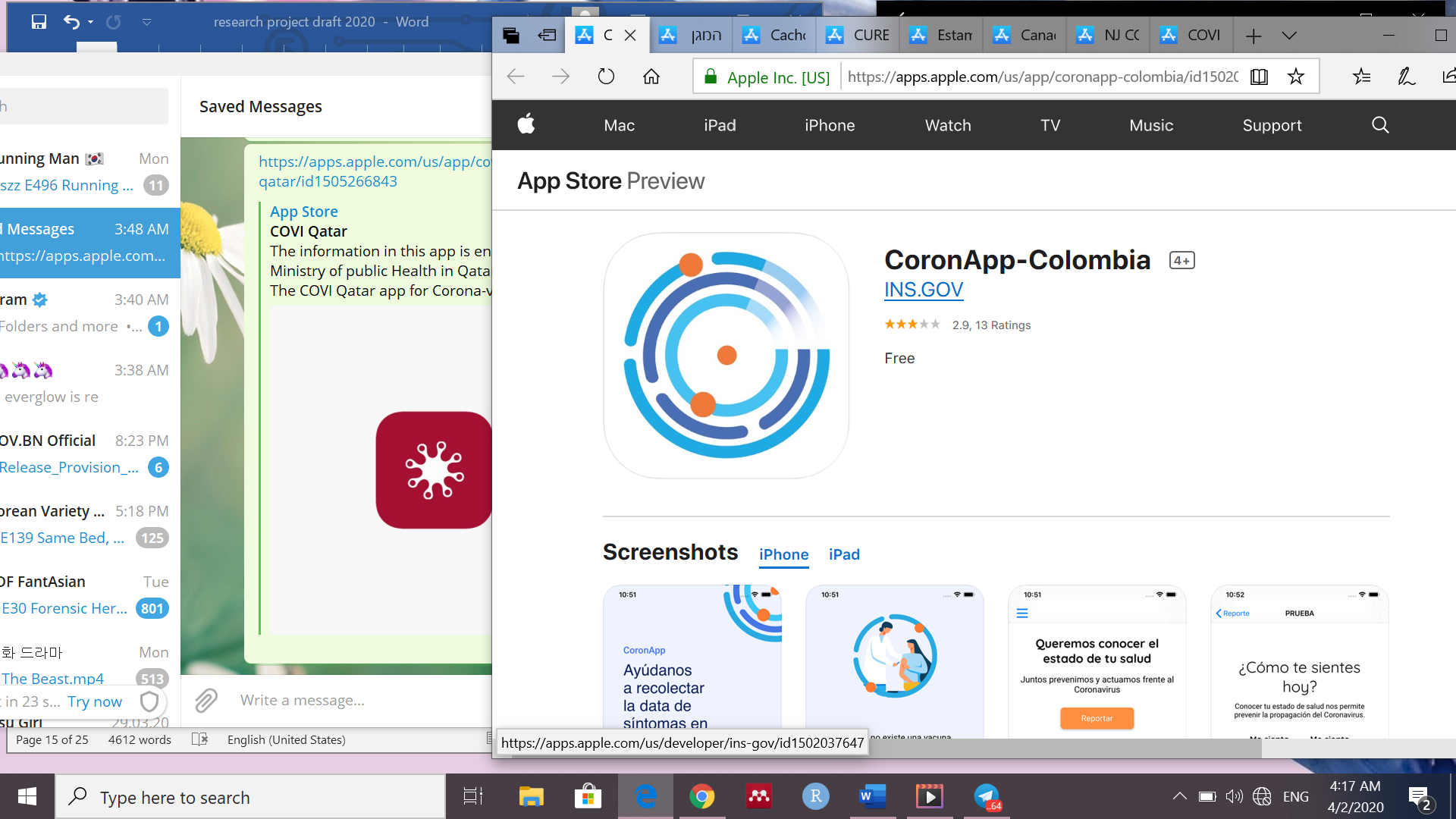 | Republic of Colombia | 20.3 MB | 3.3  (n= 11) | 4+ | Lifestyle |  | N/A |
| **35** | **CoronaMap** | National Health Information Centre, Saudi Health Council | 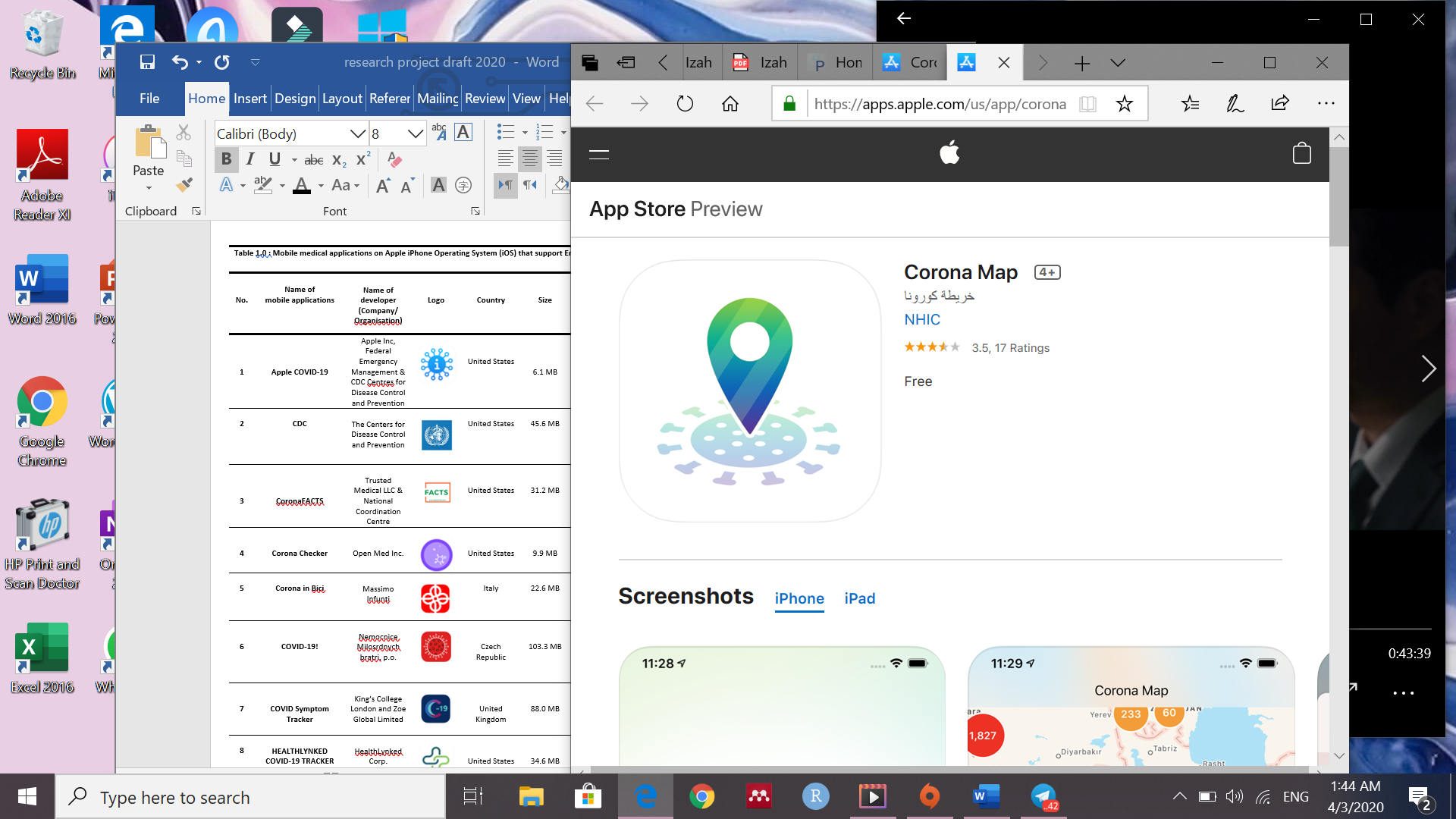 | United Arab Emirates | 16.5 MB | 3.5  (n= 17) | 4+ | Health & Fitness |  | N/A |
| **36** | **Coronavirus Bolivia** | Agencia de Gobierno Electronico y Technologias de Informacion | 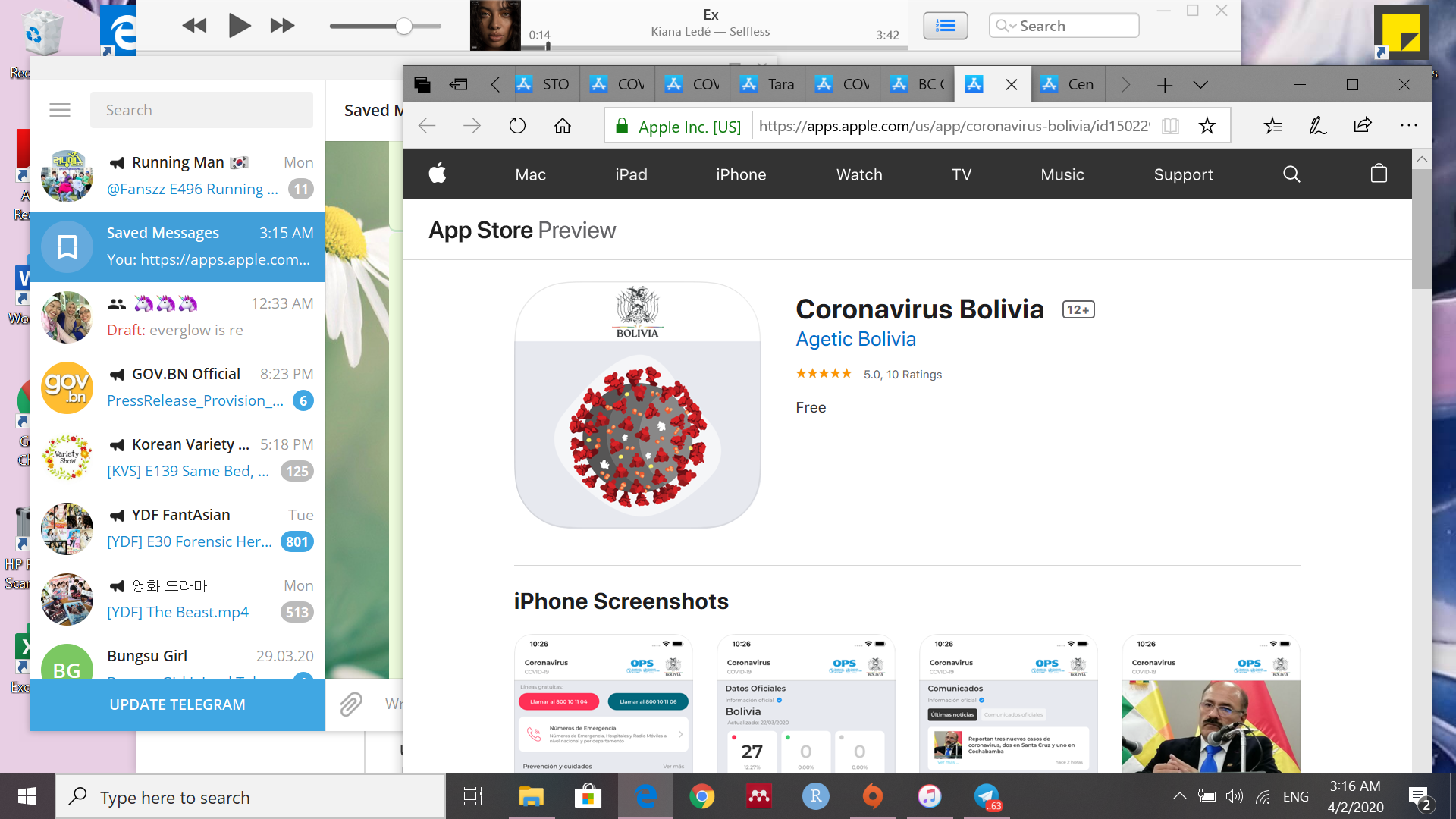 | United States | 34.1 MB | 5.0  (n= 9) | 12+ | Medical |  | N/A |
| **37** | **Coronavirus – SUS** | Governo do Brazil  (Brazilian Government) | 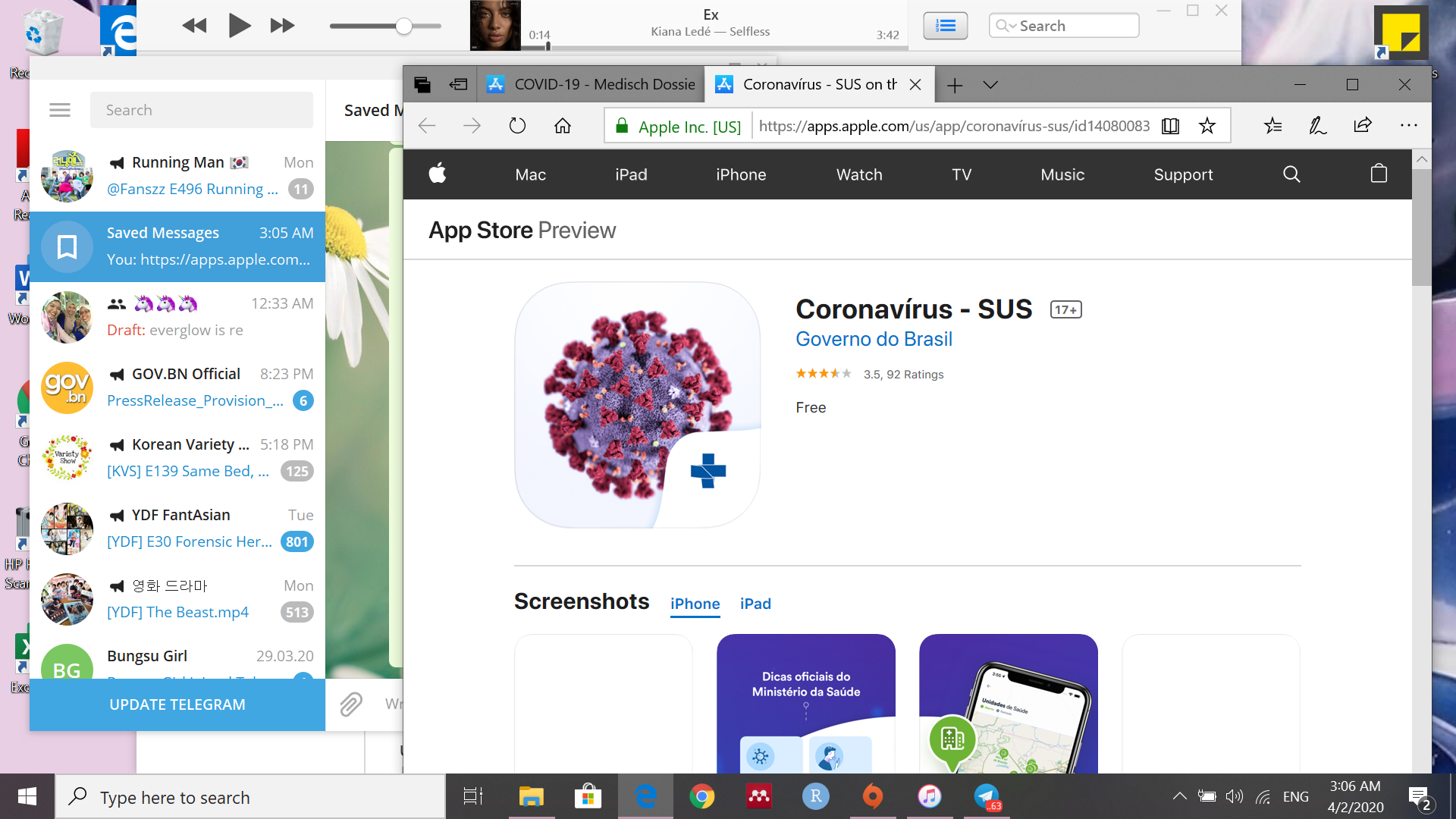 | Brazil | 43.5 MB | 3.5  (n= 90) | 17+ | Health & Fitness |  | N/A |
| **38** | **Coronavirus UY** | Agesic | 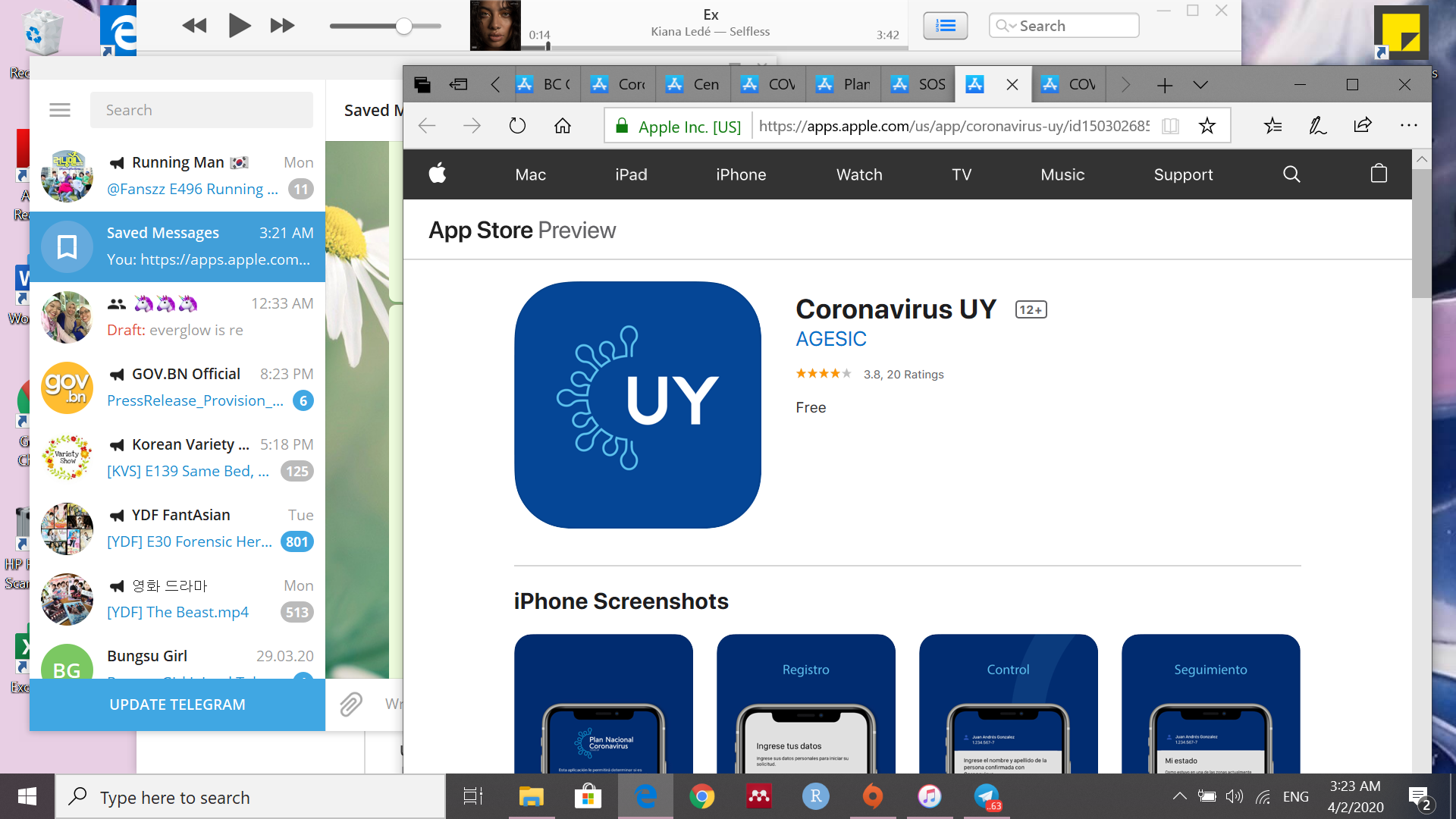 | Uruguay | 37.3 MB | 3.8  (n= 21) | 12+ | Utilities |  | N/A |
| **39** | **COVID-19** | Advanced International Joint Stock Company (AIC Group) Electronic Health Administration, Ministry of Health of Vietnam | 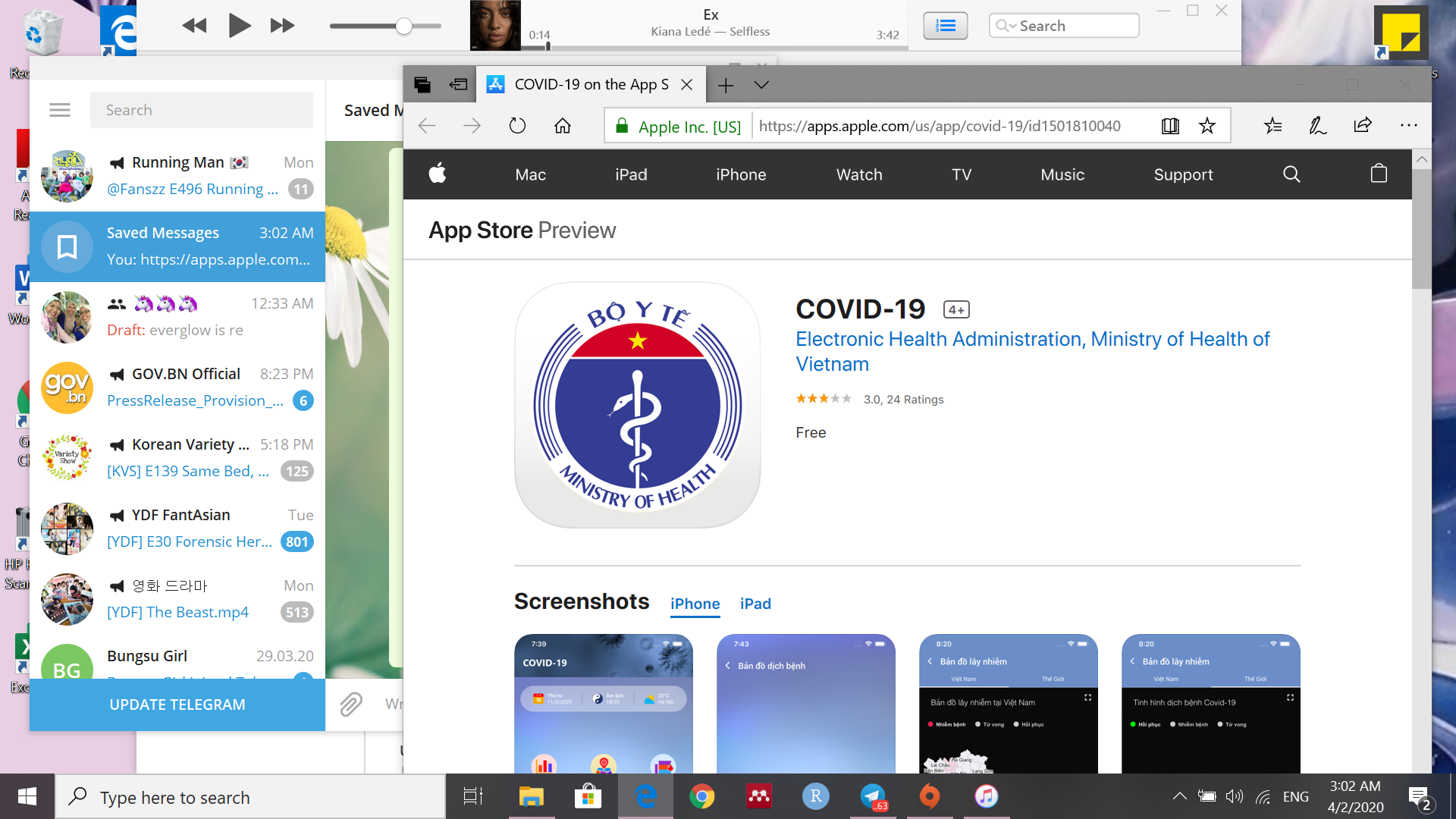 | Vietnam | 211.9 MB | 3.0  (n= 22) | 4+ | #130 Medical |  | N/A |
| **40** | **Covid-19 Armenia** | Government of Armenia | 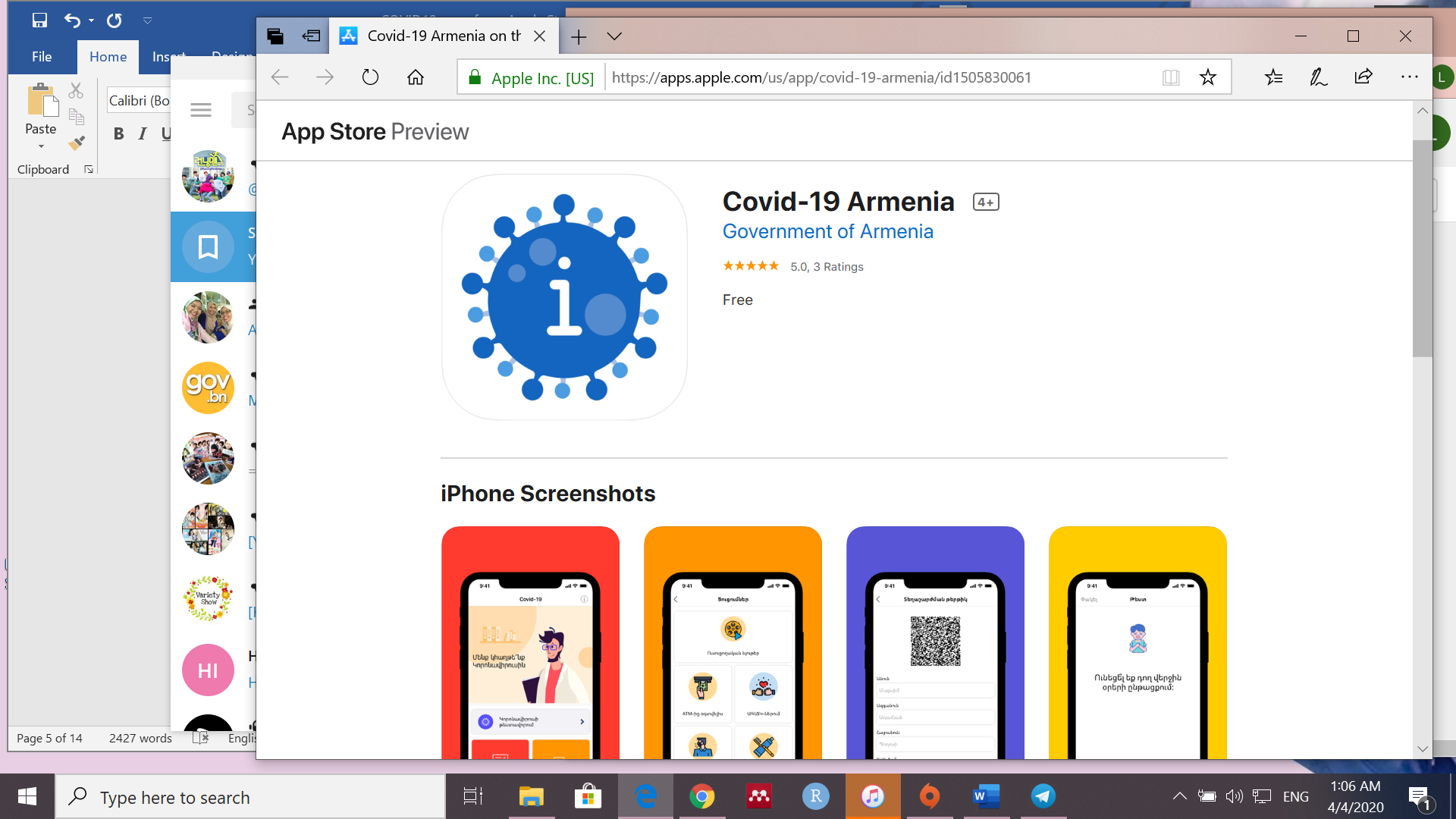 | Armenia | 18 MB | 5.0  (n= 3) | 4+ | Health & Fitness |  | N/A |
| **41** | **COVID-19 Chihuahua** | Servicios de Salud de Chihuahua | 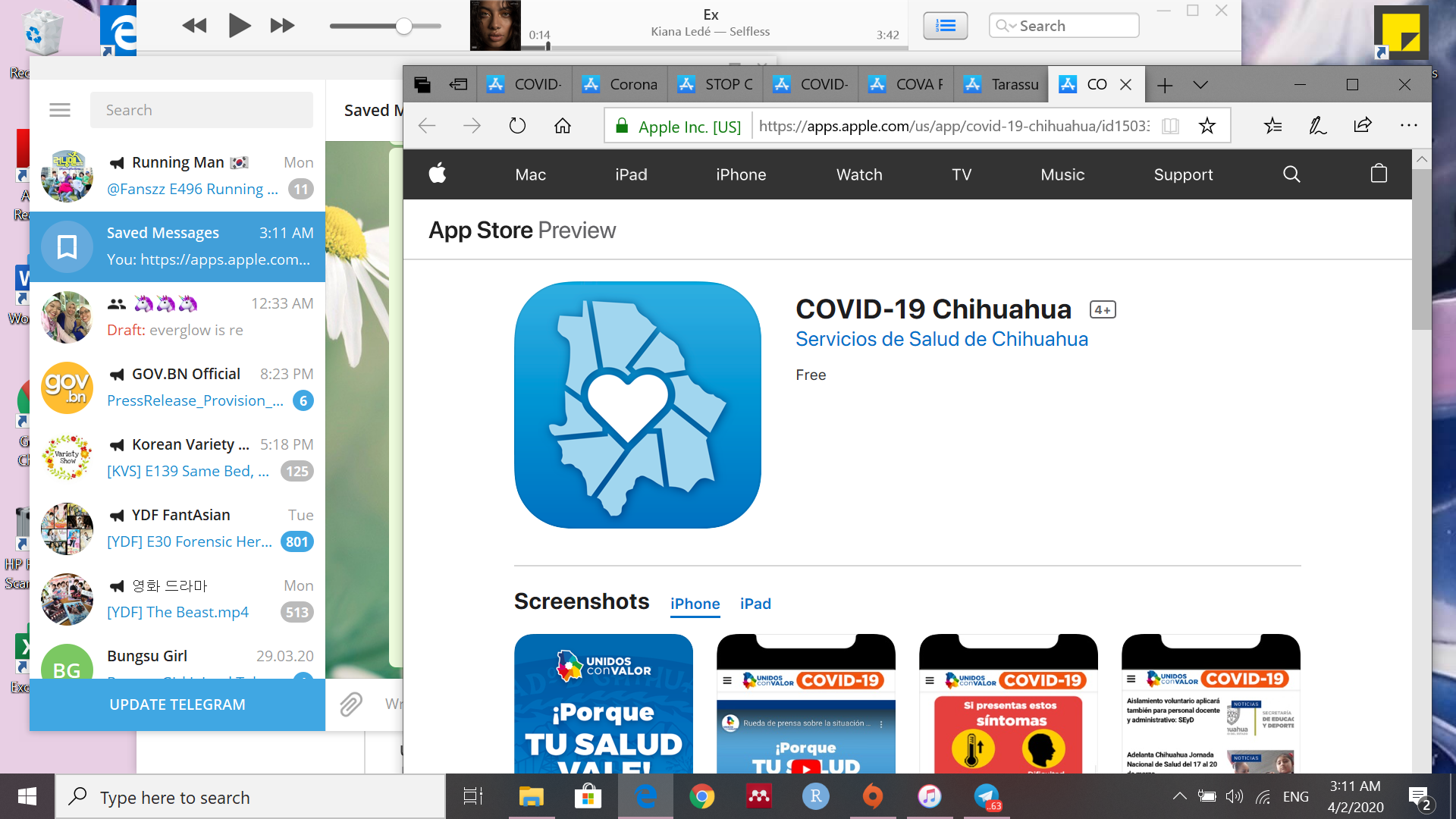 | Mexico | 7.2 MB | N/A  (n= 0) | 4+ | News |  | N/A |
| **42** | **COVID- 19 – Medisch Dossier** | MedischDossier.  org and Uitgeverij The Optimist B.V. | 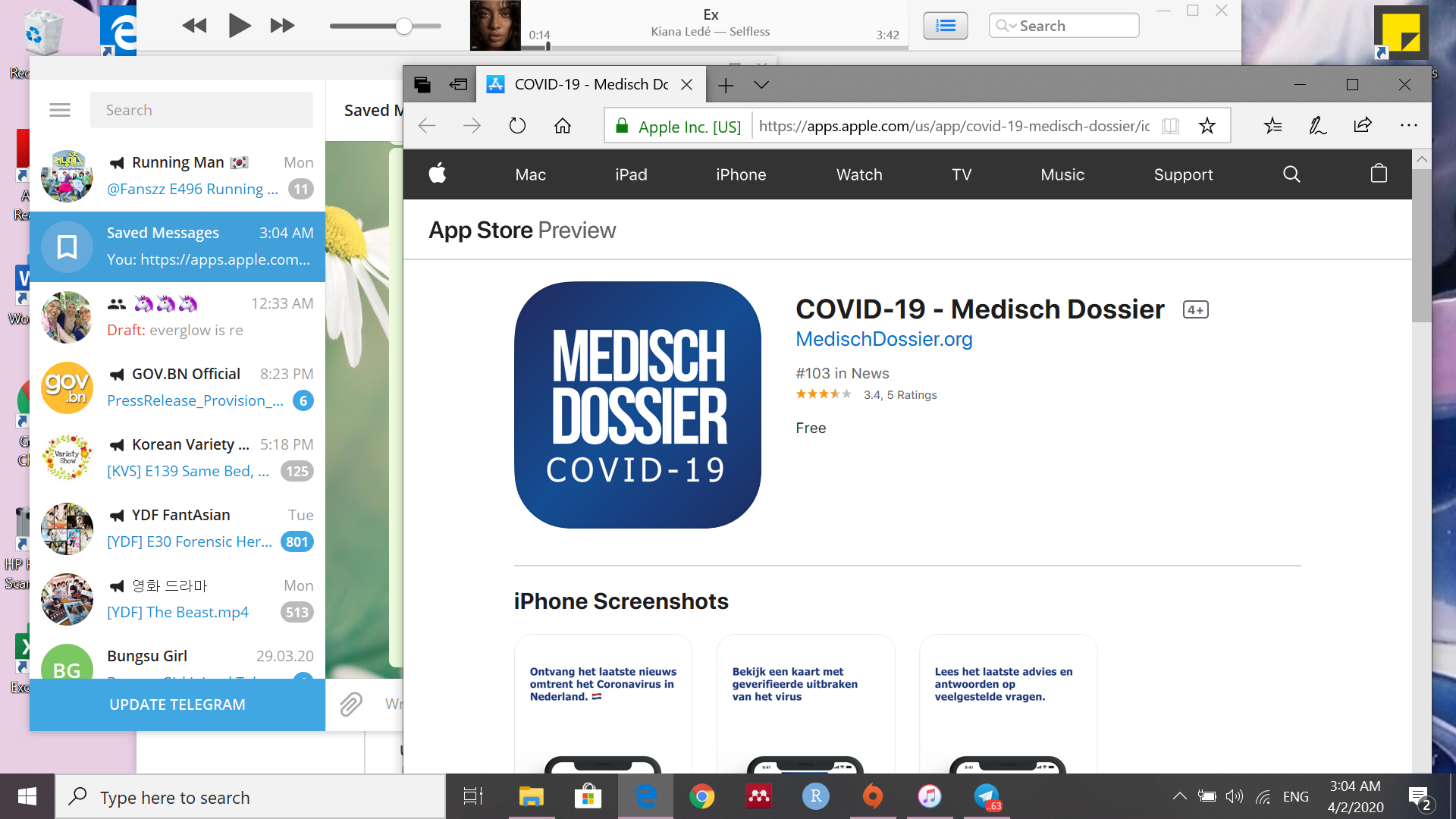 | Netherlands | 51.9 MB | 2.6  (n= 5) | 4+ | #109 News |  | N/A |
| **43** | **Covid-19 Ministerio de Salud** | Presidencia de la Nacion Argentina | 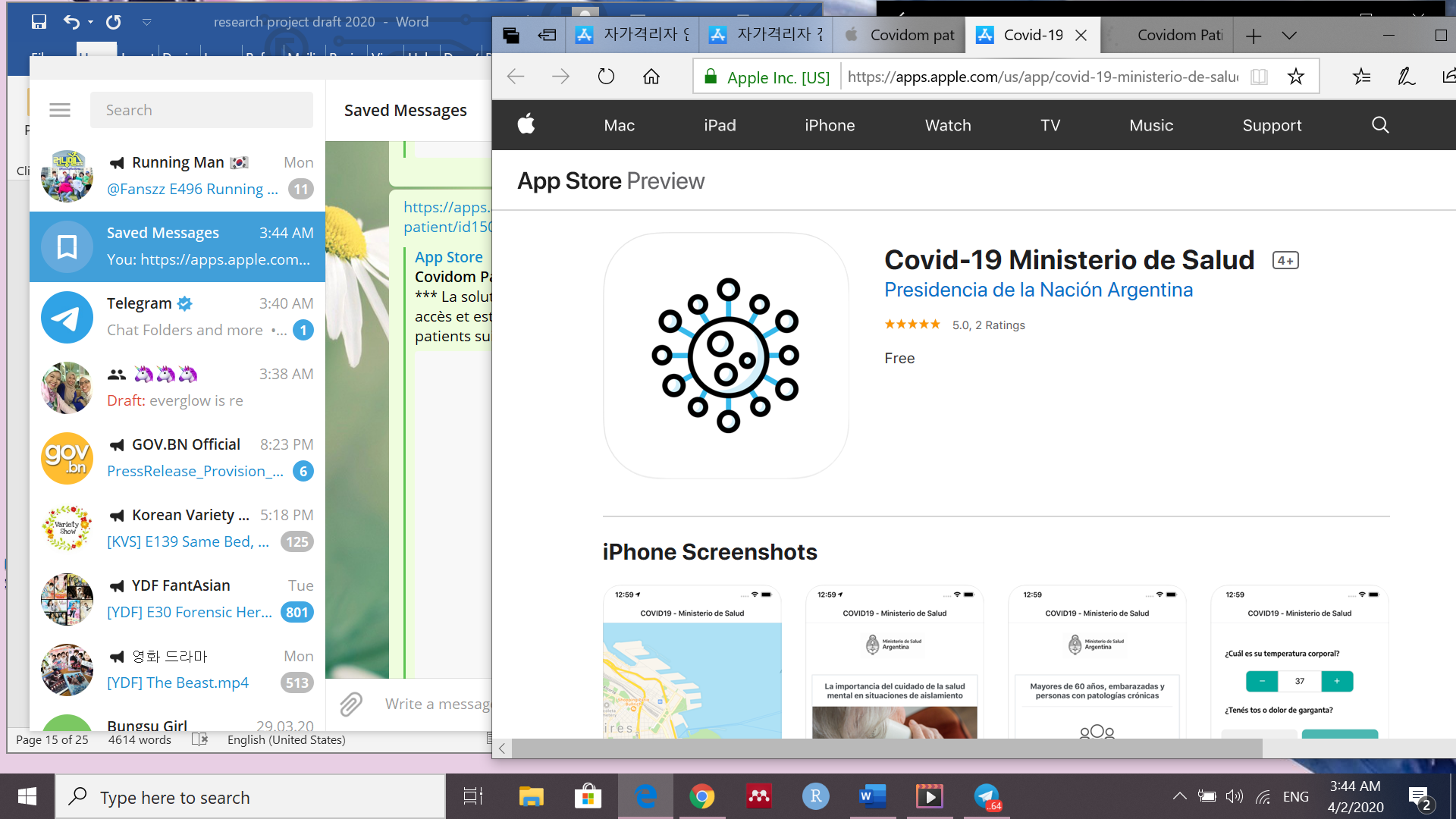 | Argentina | 55.9 MB | 5.0  (n= 2) | 17+ | Health & Fitness |  | N/A |
| **44** | **COVID19 PARANA** | Companhia de Informatica do Parana – CELEPAR | 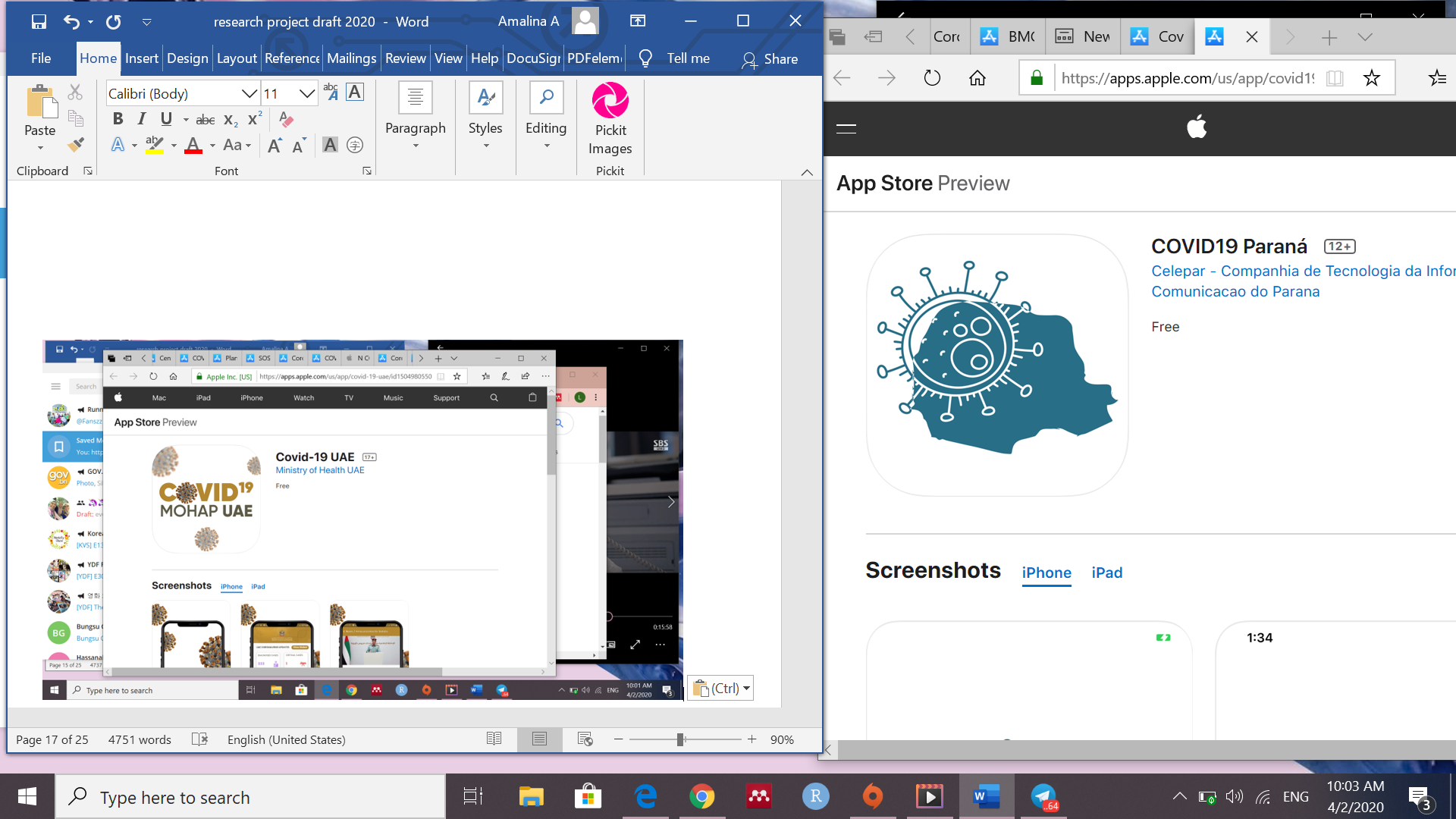 | Parana | 35.1 MB | N/A  (n= 0) | 12+ | Health & Fitness |  | N/A |
| **45** | **COVID-19**  **Regione Sardegna** | Regione Autonoma Della Sardegna | 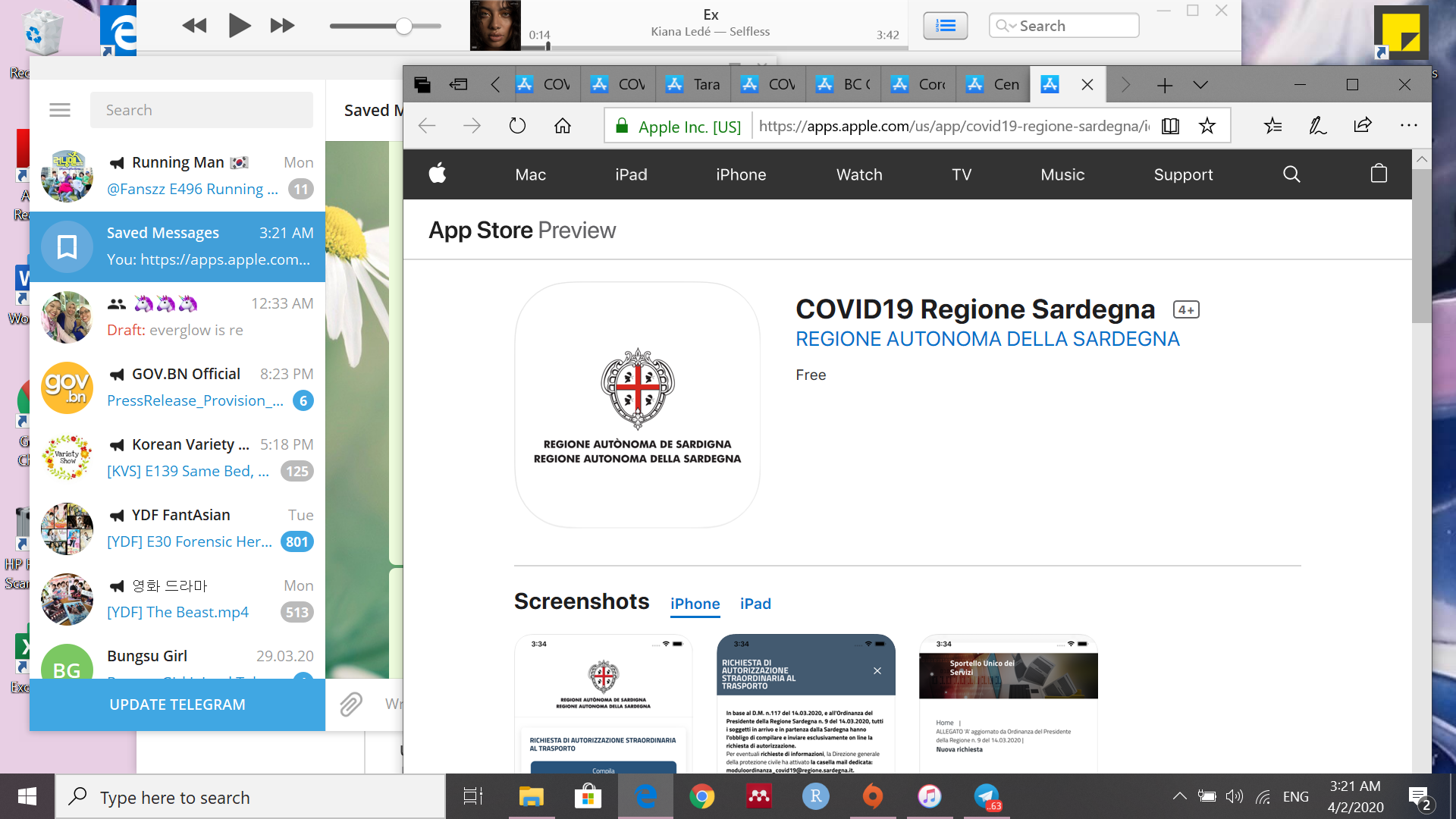 | Italy | 43.7 MB | N/A  (n= 0) | 4+ | Utilities |  | N/A |
| **46** | **Covid-19 Tam** | Gobierno del Estado de Tamaulipas | 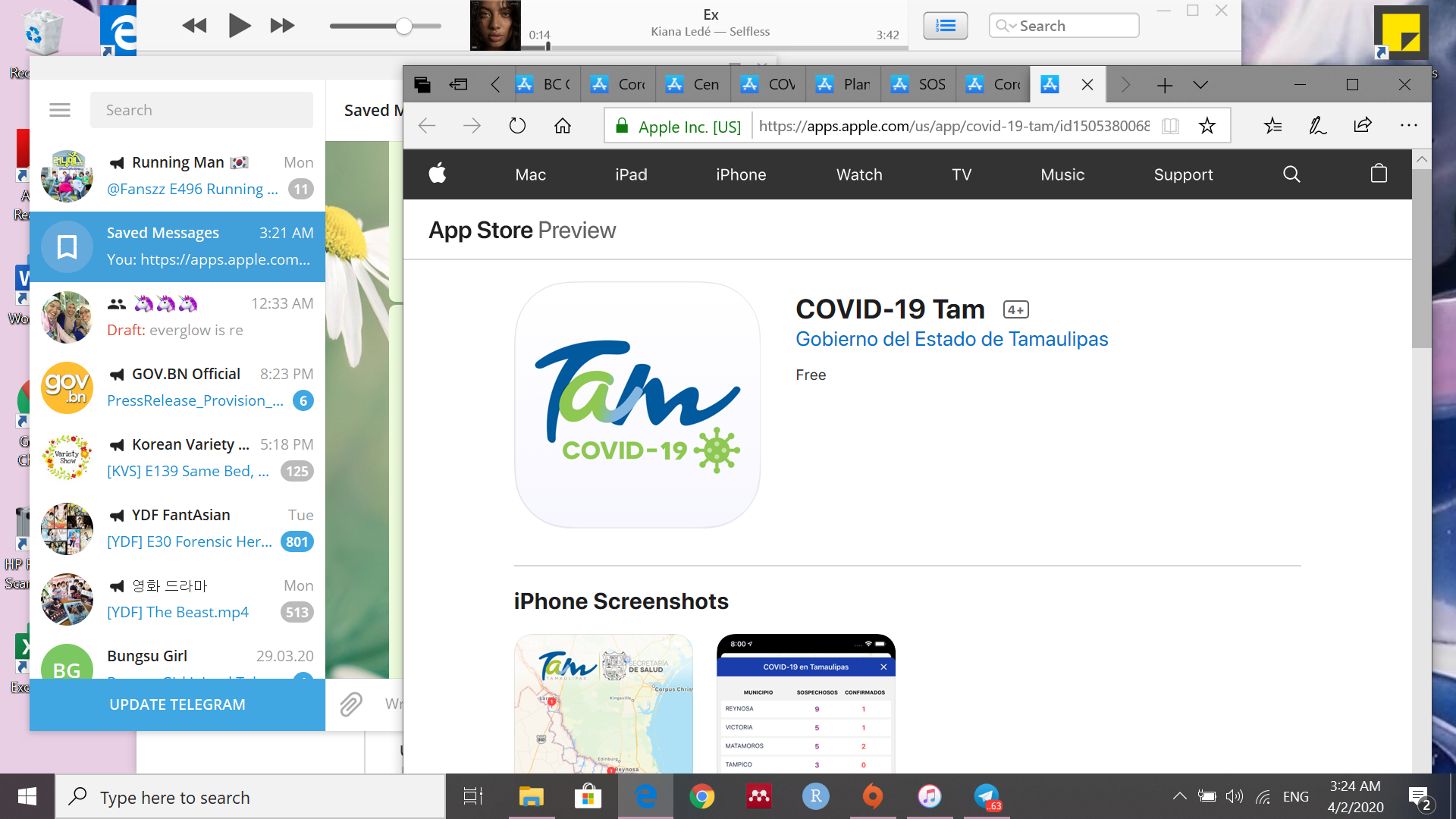 | Mexico | 14.8 MB | N/A  (n= 0) | 4+ | Health & Fitness |  | N/A |
| **47** | **Estamos ON – Covid19** | Agencia para a Modernizacao Administrativa | 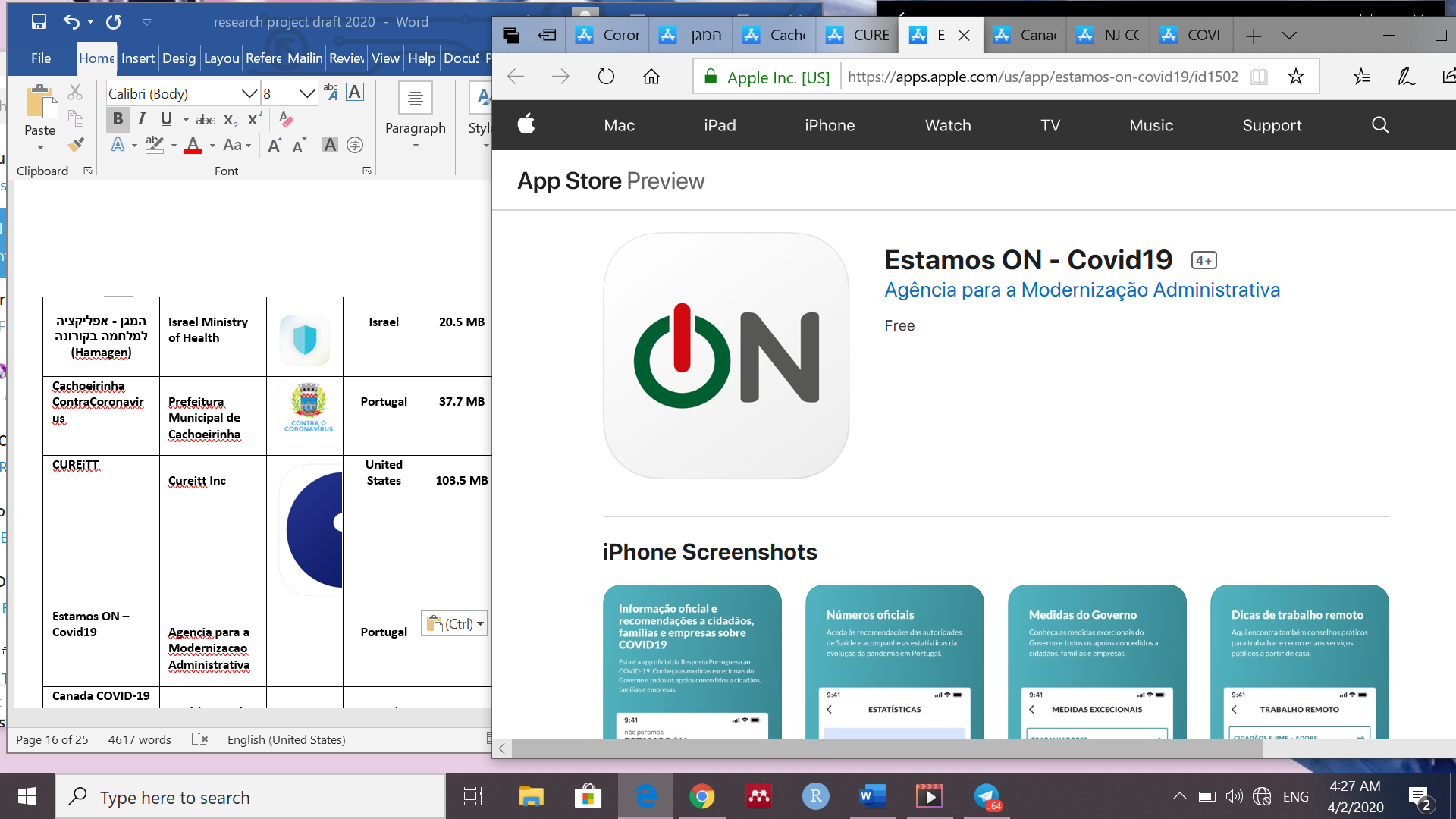 | Portugal | 33.9 MB | N/A  (n= 0) | 4+ | Utilities |  | N/A |
| **48** | **FAMILY – COVID 19** | Family Healthcare Joint Stock Company | 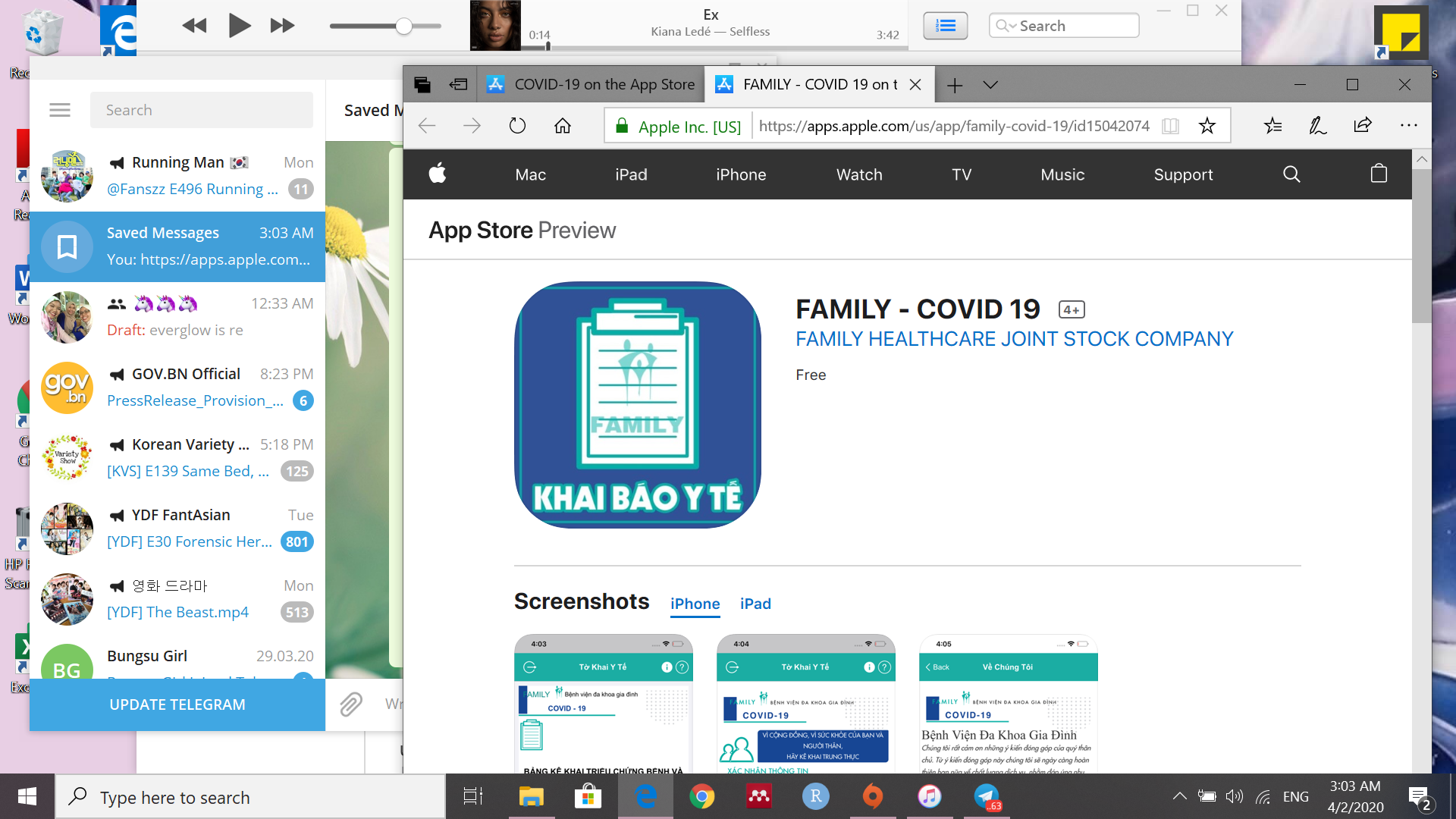 | Vietnam | 444.4 KB | N/A  (n= 0) | 4+ | Health & Fitness |  | N/A |
| **49** | **Lynx-HCF Covid-19** | Qode Health Solutions (PTY) Ltd. | 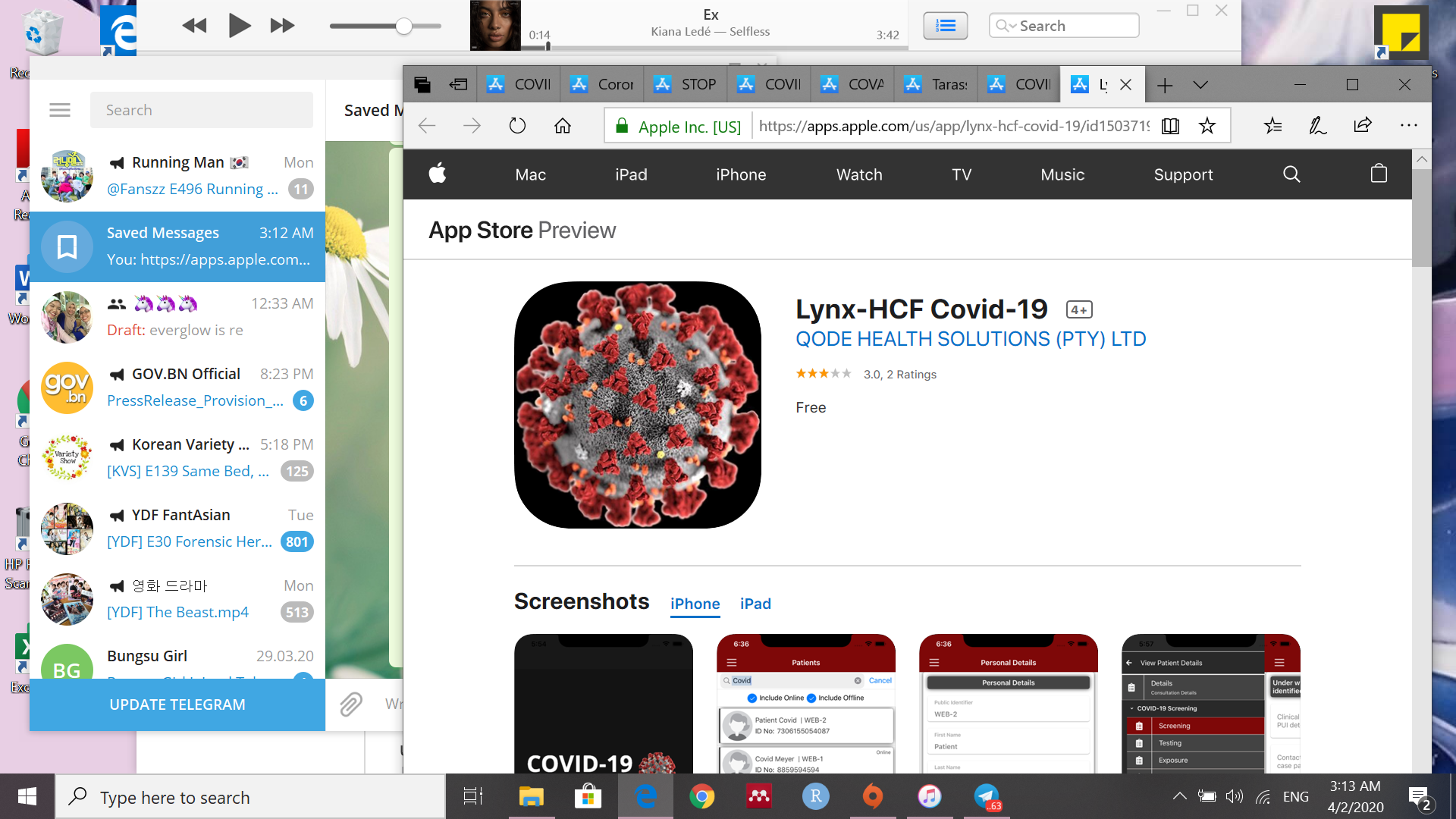 | Africa | 131.6 MB | 3.0  (n= 2) | 4+ | Medical |  | N/A |
| **50** | **Plan Jalisco Covid-19** | Secretaria de Finanzas – Gobierco del Estado de Jalisco | 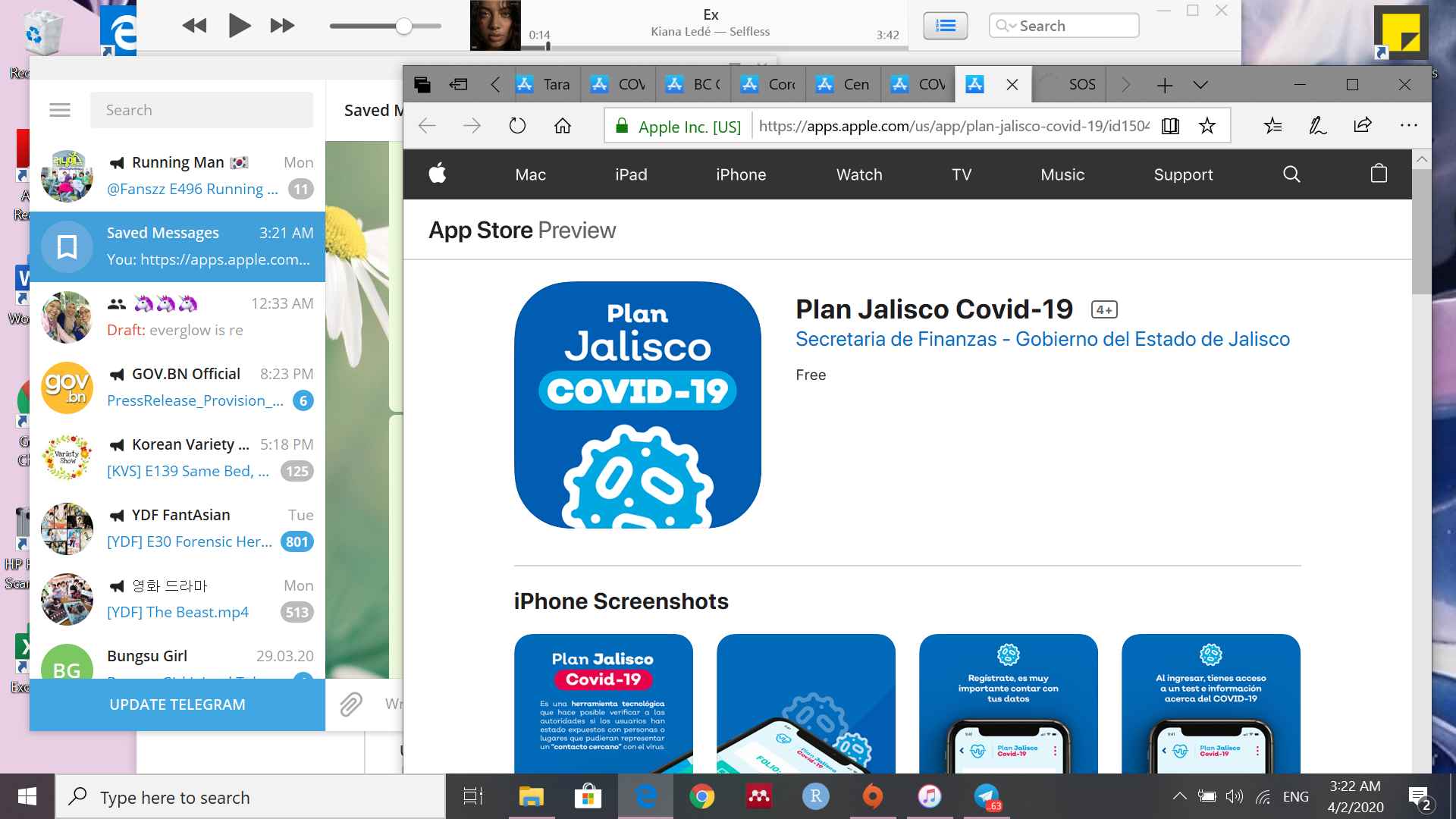 | Spain | 6.6 MB | N/A  (n= 0) | 4+ | Health & Fitness |  | N/A |
| **51** | **SOS CORONAVIRUS** | Agetic Mali | 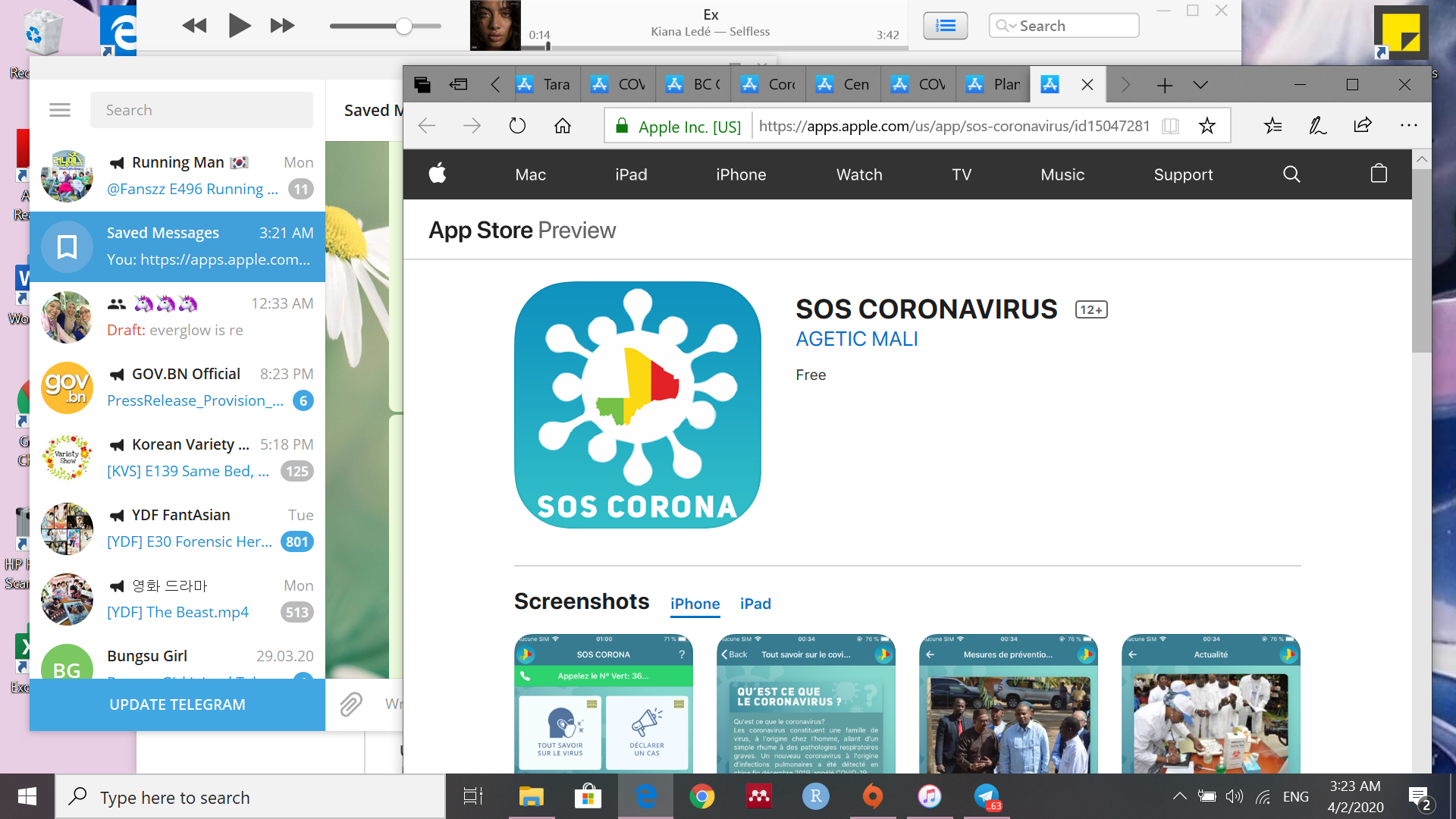 | Africa | 40.4 MB | N/A  (n= 0) | 12+ | Health & Fitness |  | N/A |
| **52** | **TreCovid19** | Azienda Provinciale per I Servizi Sanitari- Provincia Autonoma di Trento | 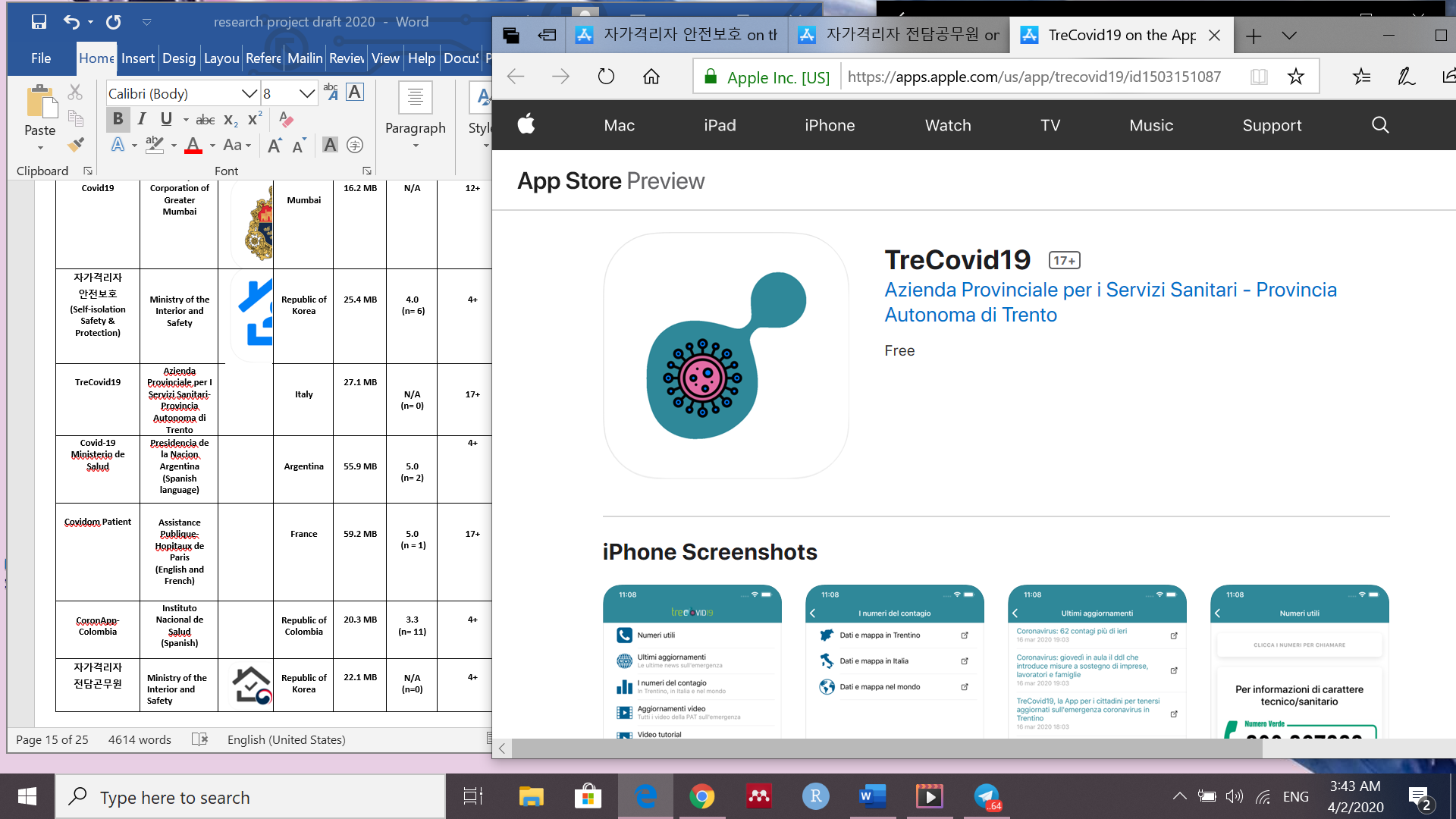 | Italy | 27.1 MB | N/A  (n= 0) | 17+ | Medical |  | N/A |
| **53** | **VirusAssist** | Healthcare X.0 GmbH | 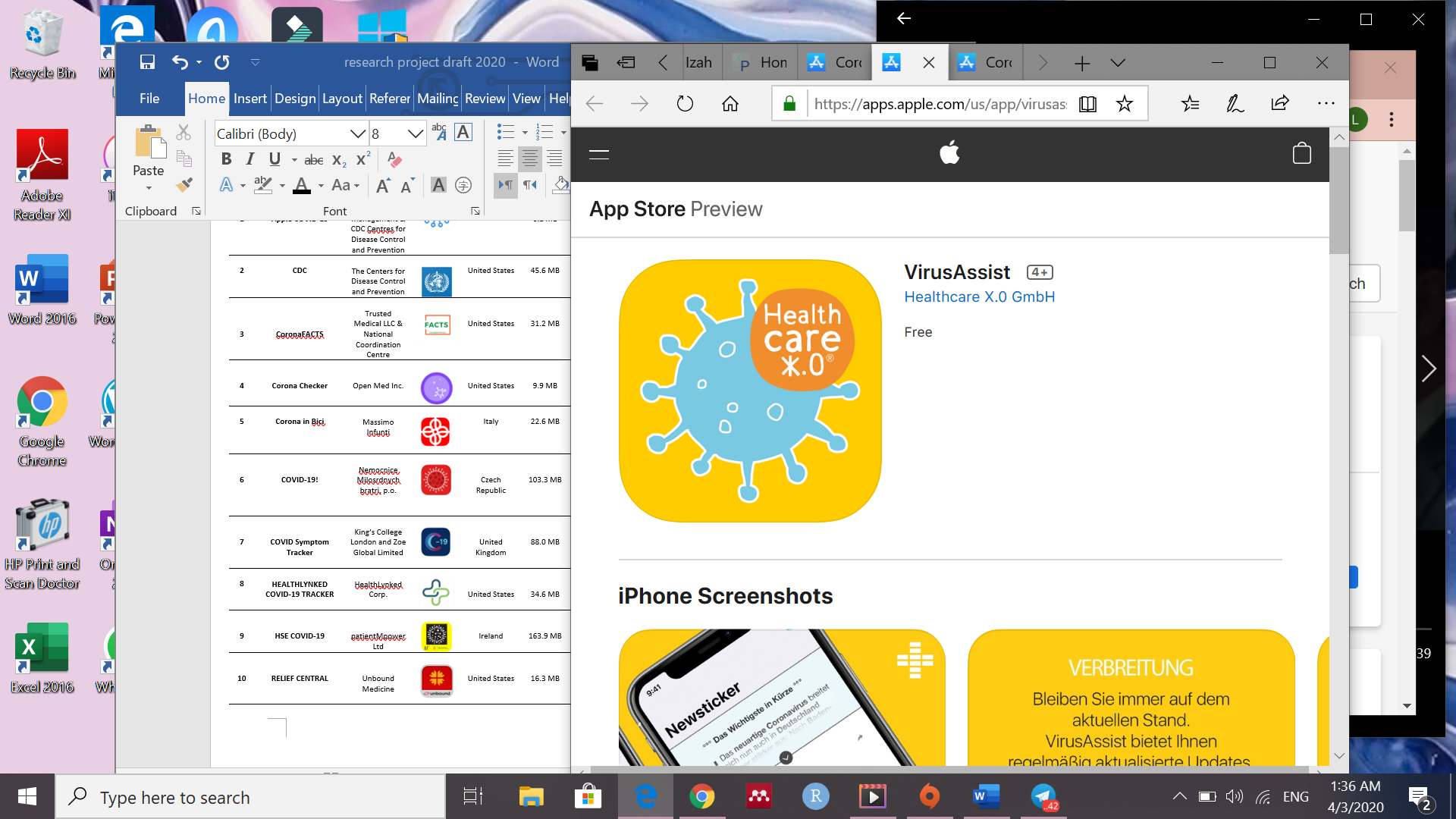 | Germany | 36  MB | N/A  (n= 0) | 4+ | Health & Fitness |  | N/A |
| **54** | **המגן - אפליקציה למלחמה בקורונה (Hamagen)** | Ministry of Health  Israel | 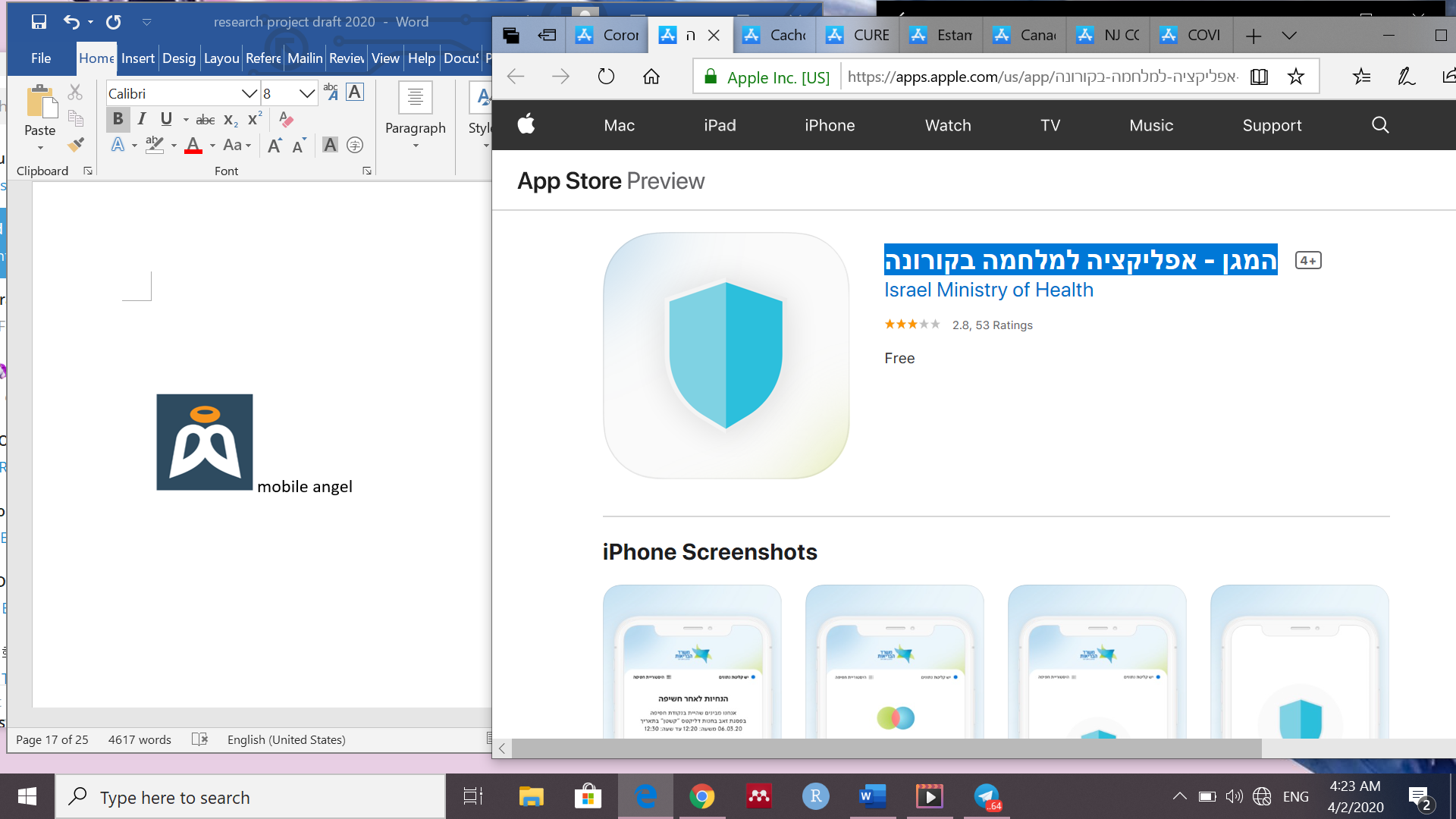 | Israel | 20.5 MB | 2.9  (n= 51) | 4+ | Health & Fitness |  | N/A |
| **55** | **Онлайн консультирование ХМАО** | Meditsinski informatsionno-analiticheskitsentr, GBU | 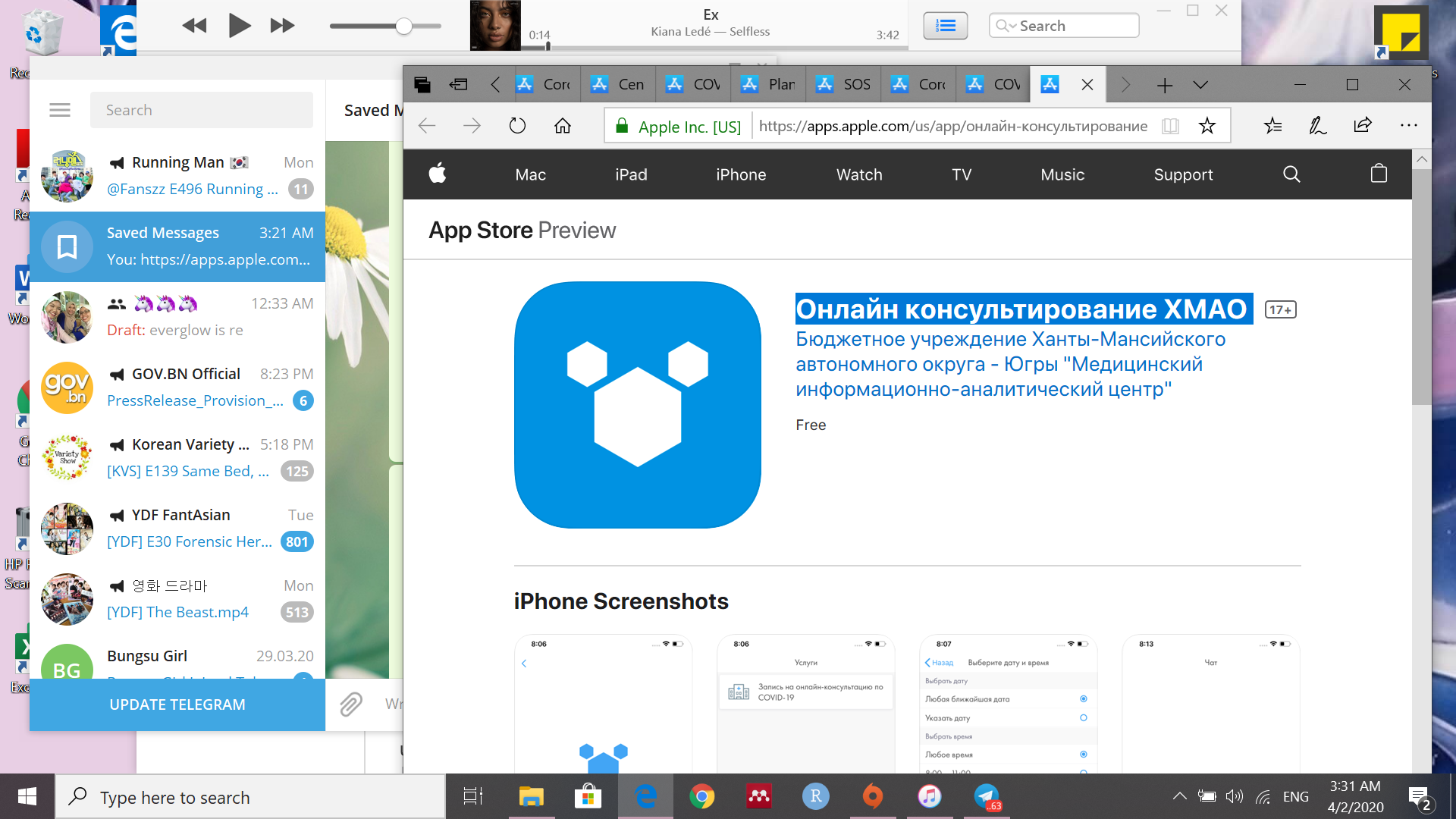 | Russia | 84.5 MB | N/A  (n= 0) | 17+ | Medical |  | N/A |
| **56** | **Госуслуги СТОП Коронавирус**  **(Government Services STOP Coronavirus)** | Minkomsyvyaz Rossil, FKU | 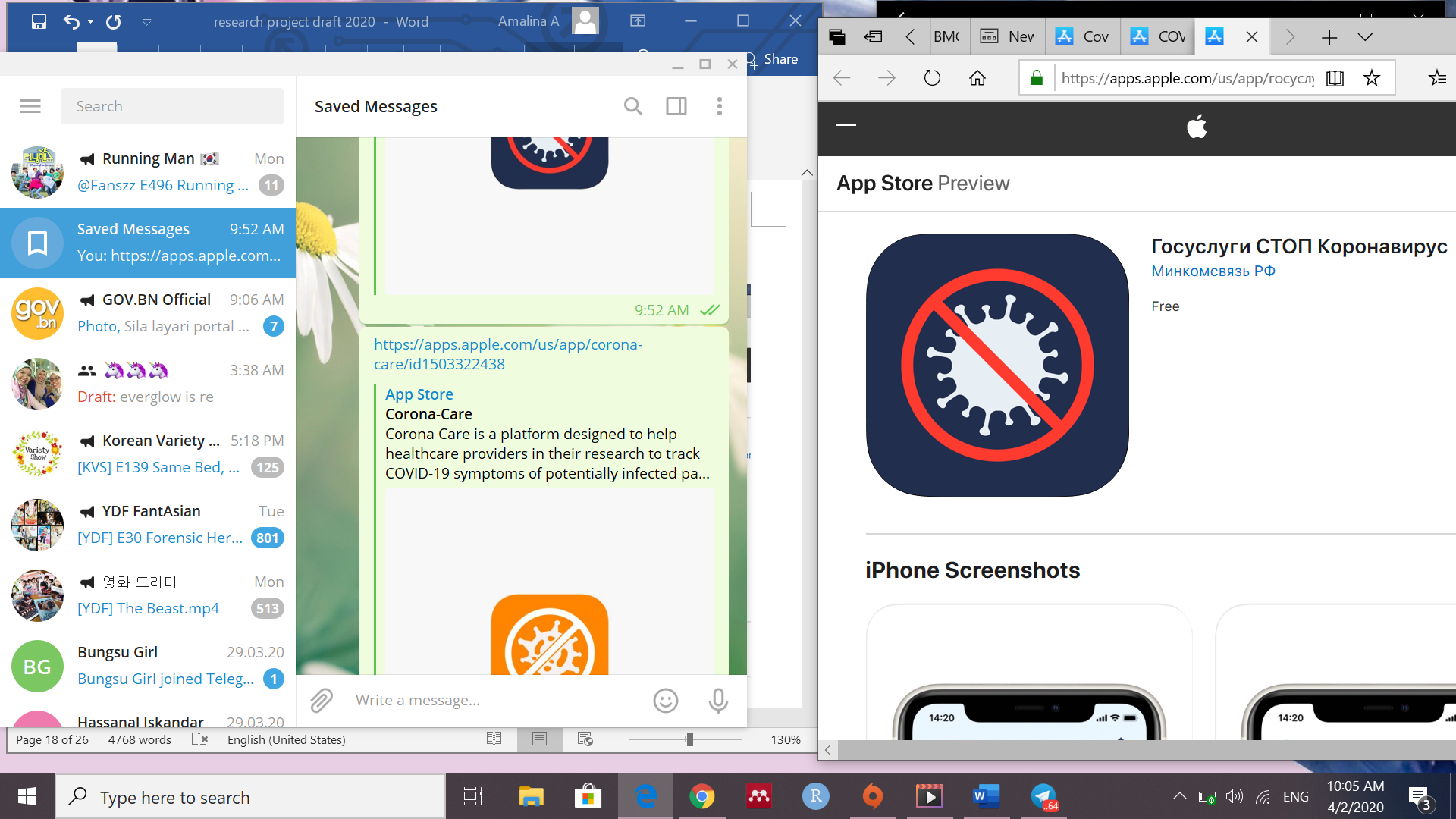 | Russia | 17.4 MB | N/A  (n= 0) | 4+ | Travel |  | N/A |
