# Supplementary material for: Mobile Health Apps on COVID-19 Launched in the Early Days of the Pandemic: Content Analysis and Review
Source: JMIR Mhealth Uhealth. 2020 Sep 16;8(9):e19796. doi: 10.2196/19796 (PMC7505686; doi:10.2196/19796)
Supplement: Multimedia Appendix 2 [file mhealth_v8i9e19796_app2.pdf]

Research paper

Content Analysis and Review of Mobile Health Applications on COVID-19

Long Chiau Ming*§1, Noorazrina Untong§1, Nur Amalina Aliudin§1, Norliza Osili§1, Nurolaini Kifli[1], Ching Siang Tan[2], Khang Wen Goh[3], Pit Wei Ng[4], Yaser Mohammed Al-Worafi[5,6], Kah Seng Lee[7], Poh Hui Goh*1

[1]PAPRSB Institute of Health Sciences, Universiti Brunei Darussalam, Gadong, Brunei Darussalam

[2]School of Pharmacy, KPJ Healthcare University College, Nilai, Negeri Sembilan, Malaysia

[3]Faculty of Science and Technology, Quest International University Perak, Ipoh, Perak, Malaysia

[4]Department of Pharmacy, National University Health System, City of Singapore, Singapore

[5]College of Pharmacy, University of Science and Technology, Sana'a, Yemen

[6]College of Pharmacy, University of Science and Technology of Fujairah, Fujairah, UAE

[7]Faculty of Pharmacy, University of Cyberjaya, Cyberjaya, Selangor, Malaysia

§equal contribution

*Corresponding authors

Long Chiau Ming, PhD; Poh Hui Goh, PhD

PAPRSB Institute of Health Sciences, Universiti Brunei Darussalam, Jalan Tungku Link Gadong BE1410, Gadong, Brunei Darussalam

long.ming@ubd.edu.bn; pohhui.goh@ubd.edu.bn

Table 2. Characteristic of mobile medical apps (android-based)

Table 2a. Universal COVID-19 apps

No.	Name of Mobile applications	Name of developer (Company/ Organisation)	Logo	Country	Size	User rating	Classification	Category	No. of downloads
1	**COVID Symptom Tracker**	King's College London and Zoe Global Ltd		United Kingdom	17 MB	2.7 (n= 2,355)	3+	Health & Fitness	500,000+
2	**Test Yourself Goa**	Innovaccer Inc.		United States	2.1 MB	4.5 (n= 64)	3+	Health & Fitness	50,000+

Table 2b. Country-specific apps

No.	Name of mobile applications	Name of developer (Company/ Organisation)	Logo	Country	Size	User rating	Classification	Category	No. of downloads
3	**Aarogya Setu**	NIC eGov Mobile Apps		India	3.6 MB	4.6 (n= 11,670)	3+	Health & Fitness	1,000,000+
4	**BC COVID-19 Support**	Province of British Columbia		Canada	6.2 MB	4.2 (n= 73)	3+	Medical	10,000+
5	**Canada COVID-19**	Health Canada		Canada	6.4 MB	4.5 (n= 69)	3+	Medical	10,000+
6	**CoBuddy – Covid19 Tool**	FaceTagR		India	38 MB	4.0 (n= 14)	3+	Tools	1,000+
7	**Corona Watch**	KSRSAC KGIS		India	4.4 MB	4.0 (n= 631)	3+	Maps & Navigation	100,000+
8	**Coronavirus Australia**	Department of Health Australia		Australia	7.1 MB	3.2 (n= 662)	3+	Health & Fitness	500,000+
9	**COVA Punjab**	Government of Punjab		India	6.0 MB	4.3 (n= 5,188)	3+	Health & Fitness	100,000+
10	**COVI**	Doobi Health Technology		Qatar	9.8 MB	4.5 (n= 31)	3+	Education	10,000+
11	**Covid-19**	ADiLife Srl		Italy	4.3 MB	2.9 (n= 66)	3+	Health & Fitness	10,000+
12	**COVID-19 NI**	Health & Social Care Northern Ireland		Ireland	46 MB	4.1 (n= 173)	3+	Medical	10,000+
13	**COVID19 Feedback**	MeitY, Government of India		India	Varies with device	3.8 (n= 146)	3+	Health & Fitness	50,000+

14	**COVID-19 Quarantine Monitor Tamil Nadu (official)**	Pixxon Al Solutions Private Limited	India	3.1 MB	3.8 (n= 92)	3+	Communication	50,000+
15	**COVID-19 West Bengal Government**	Tourism Department, Government of West Bengal	India	3.6 MB	N/A (n= 0)	3+	Social	500+
16	**GCC – Corona Monitoring**	Greater Chennai Corporation	India	6.9 MB	3.3 (n= 57)	12+	Social	5,000+
17	**GoK – Direct Kerala**	Qkopy	India	4.1 MB	4.7 (n= 13,323)	3+	Communication	100,000+
18	**Home Quarantine (Kwarantanna domowa)**	Ministerstwo Cyfryzacji	Poland	77 MB	1.4 (n= 1,739)	3+	Medical	50,000+
19	**HSE COVID-19**	patientMpower	Ireland	36 MB	N/A (n= 0)	3+	Health & Fitness	500+
20	**Mahakavasch**	Maharashtra State Innovation Society	India	4.5 MB	2.8 (n= 54)	3+	Medical	10,000+
21	**MP COVID RESPONSE APP**	National Health Mission	India	3.2 MB	4.8 (n= 26)	3+	Health & Fitness	1,000+
22	**NCOVI**	Ministry of Health and Ministry of Information and Communication	Vietnam	12 MB	4.5 (n= 24,017)	3+	Medical	1,000,000+
23	**Quarantine Watch**	Revenue Department, Government of Karnataka	India	5.3 MB	1.6 (n= 252)	3+	Health & Fitness	10,000+
24	**StayHomeSafe**	GohHK,OGCIO, HKSARG	Hong Kong	11 MB	1.5 (n= 174)	3+	Health & Fitness	10,000+

25	**Test Yourself PuduCherry**	Innovaccer Inc		United States	2.6 MB	3.9 (n= 8)	3+	Health & Fitness	5000+
26	**TraceTogether**	Ministry of Health and Government Technology Agency		Singapore	4.1 MB	3.9 (n= 1,536)	3+	Medical	500,000+
27	**자가격리자 안전보호 (Self-Isolator Safety & Protection)**	Ministry of the Interior and Safety		Republic of Korea	14 MB	2.9 (n= 210)	3+	Lifestyle	50,000+
28	**자가격리자 전담곤무원 (Self-isolating Government Officials)**	Ministry of the Interior and Safety		Republic of Korea	14 MB	N/A (n= 0)	3+	Lifestyle	10,000+

Table 2c. Non-English language apps

No.	Name of mobile applications	Name of developer (Company/ Organisation)	Logo	Country	Size	User rating	Classification	Category	No. of downloads
29	**10 Rumah Aman**	Direktorat Pengendalian Kominfo		Indonesia	10 MB	4.1 (n= 95)	3+	News & Magazines	10,000+
30	**Colab**	Colab S.A.		Brazil	16 MB	4.4 (n= 3,479)	12+	Social	100,000+
31	**CoronaMadrid**	Comunidad de Madrid		Spain	22 MB	3.4 (n= 185)	3+	Medical	50,000+
32	**Coronavirus Bolivia**	Agetic Bolivia		Bolivia	15 MB	3.7 (n= 206)	3+	Health & Fitness	10,000+
33	**CoronApp - Columbia**	INS.GOV		Columbia	10 MB	3.9 (n= 3,044)	3+	Health & Fitness	100,000+
34	**Coronavirus - SUS**	Governo do Brasil		Brazil	12 MB	4.0 (n= 9,980)	3+	Health & Fitness	1,000,000+

35	**Coronavirus Ceara**	Secretariat of Public Security of the state of Ceara		Brazil	7.4 MB	3.7 (n= 7)	3+	Medical	1,000+
36	**Coronavirus SP**	PRODESP – Cia de Proc. De Dados do Estado de SP		Brazil	28 MB	N/A (n= 0)	3+	Health & Fitness	1,000+
37	**Coronavirus UY**	AGESIC		United States	8.6 MB	4.4 (n= 1,280)	3+	Health & Fitness	100,000+
38	**COVID-19**	Electronic Health Administration		Vietnam	75 MB	4.2 (n= 327)	3+	Medical	100,000+
39	**COVID-19**	BS Software Development GmbH&Co. KG		Germany	21 MB	3.0 (n= 115)	3+	Medical	10,000+
40	**COVID-19.eus**	Osakidetza		Spain	7.7 MB	3.4 (n= 275)	3+	Medical	10,000+
41	**Covid-19 Ministerio de Salud**	Presidencia de la Nacion Argentina		Argentina	50 MB	3.4 (n= 1,231)	3+	Medical	500,000+
42	**COVID-19 Parana**	Celepar		Brazil	7.1 MB	N/A (n= 0)	3+	Health & Fitness	500+
43	**COVID19 Regione Sardegna**	Regione Autonoma Della Sardegna		Italy	7.1 MB	N/A (n= 0)	3+	Tools	10+
44	**COVID AP-HM**	RADHIUS		France	4.1 MB	N/A (n= 0)	3+	Medical	5,000+
45	**Covidom Patient**	Assistance Publique Hopitaux de paris		France	3.9 MB	2.5 (n= 50)	3+	Medical	10,000+
46	**GVA responde**	Generalitat Valenciana		Spain	1.9 MB	N/A	3+	Communicatio n	0
47	**Korina Onlem**	T.C. Saglik Bakanligi		Turkey	50 MB	4.1 (n= 137)	3+	Health & Fitness	50,000+

**48**	**LAZIOdrCOVID**	LAZIOcrea s p A		Italy	3.8 MB	3.0 (n= 428)	3+	Medical	50,000+
**49**	**PeduliLindungi**	P.T. Telekomunikasi Indonesia, Tbk.		Indonesia	3.5 MB	4.1 (n=457)	3+	Medical	100,000+
**50**	**PiKOBAR West Java**	West Java Provincial Government		Indonesia	15 MB	4.3 (n= 3,349)	3+	News & Magazines	500,000+
**51**	**Plan Jalisco Covid-19**	Gobierno del Estado de Jalisco		Mexico	23 MB	N/A (n= 0)	3+	Health & Fitness	100+
**52**	**SESA Monitoramento**	Celepar		Brazil	6.1 MB	2.3 (n= 12)	3+	Health & Fitness	5,000+
**53**	**STOP COVID19 CAT**	Generalitat de Catalunya		Spain	3.1 MB	3.3 (n= 1,248)	3+	Medical	500,000+
**54**	**המגן - אפליקציה למלחמה בקורונה (Hamagen)**	Israel Ministry of Health		Israel	22 MB	3.0 (n= 2,654)	3+	Health & Fitness	500,000+
